# Combined In Vitro Studies and in Silico Target Fishing for the Evaluation of the Biological Activities of *Diphylleia cymosa* and *Podophyllum hexandrum*

**DOI:** 10.3390/molecules23123303

**Published:** 2018-12-13

**Authors:** Marina Pereira Rocha, Priscilla Rodrigues Valadares Campana, Denise de Oliveira Scoaris, Vera Lucia de Almeida, Julio Cesar Dias Lopes, Julian Mark Hugh Shaw, Claudia Gontijo Silva

**Affiliations:** 1 Servico de Biotecnologia Vegetal, Fundacao Ezequiel Dias (FUNED), Belo Horizonte 30510-010, MG, Brazil; procha.marina@gmail.com; 2 Departamento de Produtos Farmaceuticos FAFAR-UFMG, Belo Horizonte 31270-901, MG, Brazil; prvcampana@gmail.com; 3 Servico de Fitoquimica e Prospeccao Farmaceutica, Fundacao Ezequiel Dias, Belo Horizonte 30510-010, MG, Brazil; deniscoaris@gmail.com (D.d.O.S.); veluca2002@gmail.com (V.L.d.A.); 4 Chemoinformatics Group (NEQUIM), Departamento de Quimica, Universidade Federal de Minas Gerais, Belo Horizonte 31270-901, MG, Brazil; jlopes.ufmg@gmail.com; 5 Science and Collections, Royal Horticultural Society, Wisley, Working, Surrey GU23 6QB, UK; julianshaw@rhs.org.uk

**Keywords:** lignans, in silico studies, podophyllotoxin, antibacterial activity, acetylcholinesterase inhibitors, antioxidant activity, cytotoxicity

## Abstract

This paper reports the in silico prediction of biological activities of lignans from *Diphylleia cymosa* and *Podophyllum hexandrum* combined with an in vitro bioassays. The extracts from the leaves, roots and rhizomes of both species were evaluated for their antibacterial, anticholinesterasic, antioxidant and cytotoxic activities. A group of 27 lignans was selected for biological activities prediction using the Active-IT system with 1987 ligand-based bioactivity models. The in silico approach was properly validated and several ethnopharmacological uses and known biological activities were confirmed, whilst others should be investigated for new drugs with potential clinical use. The extracts from roots of *D. cymosa* and from rhizomes and roots of *P. hexandrum* were very effective against *Bacillus cereus* and *Staphylococcus aureus*, while podophyllotoxin inhibited the growth of *Staphylococcus aureus* and *Escherichia coli*. *D. cymosa* leaves and roots showed anticholinesterasic and antioxidant activities, respectively. The evaluated extracts showed to be moderately toxic to THP-1 cells. The chromatographic characterization indicated that podophyllotoxin was the major constituent of *P. hexandrum* extract while kaempferol and its hexoside were the main constituents of *D. cymosa* leaves and roots, respectively. These results suggest that the podophyllotoxin could be the major antibacterial lignan, while flavonoids could be responsible for the antioxidant activity.

## 1. Introduction

**Figure 1:**
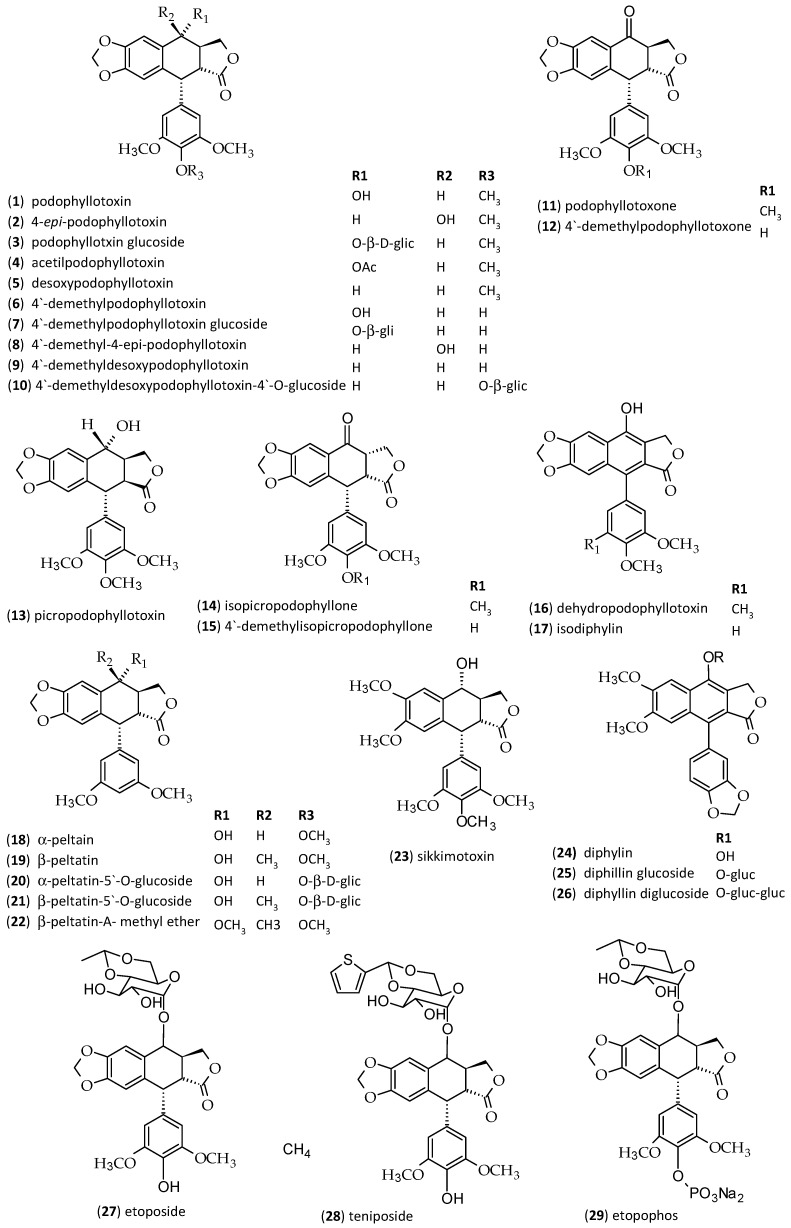
Chemical structures of lignans and podophyllotoxin derivatives [13,25].

Lignans are a large group of phenylpropanoid dimers with a different degree of oxidation in the side-chain and a different substitution in the aromatic group [1,2]. They are classified in groups according to their oxygenation and cyclization patterns. The most prominent member of this group of natural products is podophyllotoxin (PTOX, **1**). Its antitumour activity prompted several studies, and resulted in the introduction of successful clinical drugs. This aryltetralin lignan is a lead compound for the semi-synthetic derivatives etoposide (**27**), teniposide (**28**), and etopophos (**29**) (Figure 1), which have an important role in cancer therapy [3,4]. In addition, analogues of podophyllotoxin (**1**) were evaluated for the treatment of rheumatoid arthritis, psoriasis, and malaria with good results [3,5]. Furthermore, there are reviews published referring to the semisynthesis of PTOX derivatives, applications, mode of action and structure-activity relationships [6,7,8].

Arylnaphatalene lignans such as diphyllin (**24**) and its glycosides **25** and **26** (Figure 1) were isolated from some traditional medicinal plants and have been reported to possess a wide range of pharmacological activities, including antitumour, anti-leshmania, antifungal, antiviral and antibacterial [9,10].

Currently, there are few plant sources of podophyllotoxin (**1**) and its related lignans occur in a particular taxonomic group, but in low amounts. Podophyllotoxin (**1**) is still obtained from wild *Podophyllum* populations, and this is a major constraint in supplying the lignan to the pharmaceutical industry that is under pressure to meet demand. To overcome this situation, several studies focussing on its production by biotechnological strategies and synthetic approaches have been reported [11,12].

Podophyllotoxin (**1**) is found in the rhizomes, roots and leaves of both *Podophyllum hexandrum* Royle and *Diphylleia cymosa* Michaux (Berberidaceae), while the occurrence of diphyllin (**24**) is reported in the latter species but not in *Podophyllums* [13]. *P. hexandrum* and *D. cymosa* are herbaceous perennials found growing in moist shady conditions [14], and are known for their medicinal use in American and Asian cultures. Both genera are taxonomically closely related, and some common features are their habitat, morphology, karyotype and chemical profile, whilst the differences are related to floral biology [13,15,16]. A study based on four molecular markers and morphology confirms the close relationship between *Diphylleia* and *Podophyllum* [17]. *P. hexandrum* is sometimes treated as a monotypic genus *Sinopodophyllum* [18].

*P. hexandrum* is commonly named as the Himalayan Mayapple or Indian Mayapple and Indian *Podophyllum*. There are ethnobotanical records based on its healing properties in Asian culture for the treatment of skin cancers as well as due to its purgative, emetic, cytotoxicity, antitumour and antileukaemic properties [11,19]. Overall, the parts used for medicinal purposes are mainly the rhizomes, roots and fruits. *D. cymosa* has been called the Southern Mayapple and Umbrella Leaf [20]. There is an account that describes the American Cherokee Indians using an infusion of the plant as a diuretic, antiseptic, diaphoretic and for the treatment of smallpox [21]. According to an earlier clinical study, the resin demonstrated none of the biological properties associated with *Podophyllum* [22]. Considerable interest has been centered on *P. hexandrum* due to the PTOX content and its related lignans. With regard to *D. cymosa*, the species has been investigated less than the *Podophyllums* and only a few studies have been reported [13,23,24].

**Figure 2:**
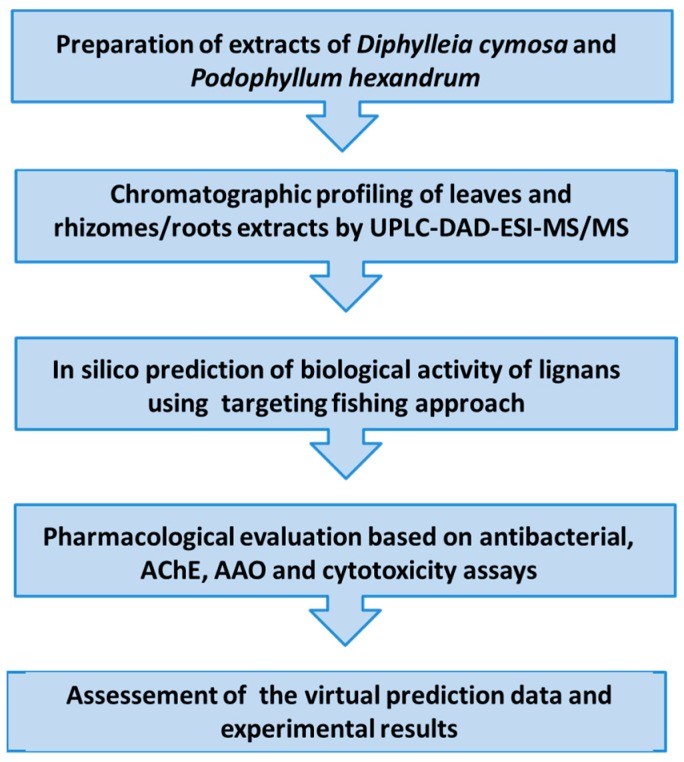
Workflow of the methodology performed in this study.

This paper reports on the evaluation of ethanolic extracts from the leaves and roots of *D. cymosa*, and from the rhizomes and roots of *P. hexandrum* for the antibacterial activity, inhibition of AChE, as well as for the antioxidant activity and cytotoxicity combined with an in silico target fishing approach. The latter was used to predict new activities for known lignans from both species. The lignans profiling was based on the chromatographic analyses (UPLC-DAD-ESI-MS/MS) which were included with the aim of identifying the major phenolic components in the extracts (Figure 2).

## 2. Results

A growing amount of work has been applied to investigating *P. hexandrum* due to its content of PTOX (**1**) and related lignans. However, it is surprising to note that studies into *D. cymosa* are extremely limited when compared with the *Podophyllum* species, even though this species is endemic in the Southern Appalachian Mountains of the Eastern North America [22,26].

There has been a decrease in the wild populations of *P. hexandrum* in India due to the over collection of rhizomes and roots of this species [27]. In addition, the species shows a short season of availability, and thus plants are limited in the field. A number of studies have been undertaken to achieve mass propagation [28] as well as to establish plant derived-cultures for the production of podophyllotoxin [29,30], whereas their low yields were far from meeting commercial needs. Enhancement of the lignan was attempted by other systems, including transgenic cultures, addition of a precursor feeding to the culture medium, the use of an elicitor such as methyl jasmonate, and the production by endophytes [11]. With regard to the latter, there are reports on the production of PTOX (**1**) by the endophytic fungi *Fusarium solani* [31] and *Trametes hirsuta* [32] isolated from *P. hexandrum*. These species of endophytes could be a promising source for large-scale production of PTOX, whereas the yields must be improved.

### 2.1. Chromatographic Profiling by UPLC-DAD-ESI-MS/MS

Several lignans have been already reported for *D. cyomsa* and *P. hexandrum*. From the leaves and roots of *D. cymosa*, Broomhead and Dewick [13] isolated the lignans PTOX (**1**), 4′-demethyl-podophyllotoxin (**6**), 4′-demethyldesoxypodophyllotoxin (**9**), diphyllin (**24**), diphyllin glucoside (**25**), diphyllin diglucoside (**26**) and 4′-demethyldesoxypodophyllotoxin 4-*O*-glucoside (**10**) (Figure 1), this being the only study reporting the isolation and characterization of compounds from *D. cymosa*.

A phytochemical study of *P. hexandrum* led to the isolation of the lignans PTOX (**1**), 4′-demethylpodophyllotoxin (**6**), 4′-demethyldesoxypodophyllotoxin (**9**), PTOX glucoside (**3**), desoxy-podophyllotoxin (deoxypodophyllotoxin, **5**), 4′-demethylpodophyllotoxin glucoside (**7**), 4′-demethylisopicropodophyllone (**15**), podophyllotoxone (**11**), 4′-demethylpodophyllotoxone (**12**), picropodophyllotoxin (**13**), isopicropodophyllone (**14**), 4′-demethyldeoxypodophyllotoxin (4′-demethyldesoxypodophyllotoxin, **9**), α-peltatin (**18**) and β-peltatin (**19**) (Figure 1) [13]. 

**Figure 3:**
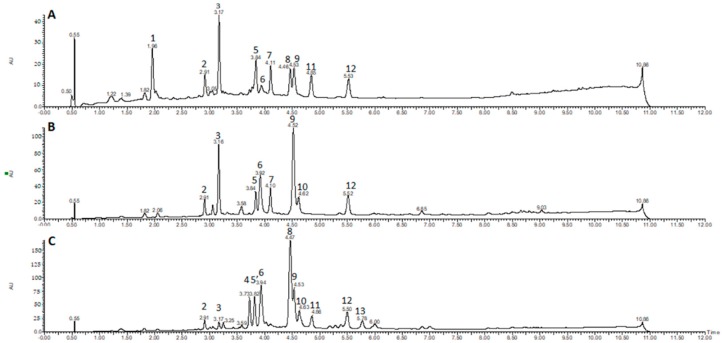
Chromatographic profiles obtained by UPLC-DAD for the EtOH extracts of *D. cymosa* leaves (**A**) and roots (**B**) and *P. hexandrum* rhizomes and roots (**C**).

**Figure 4:**
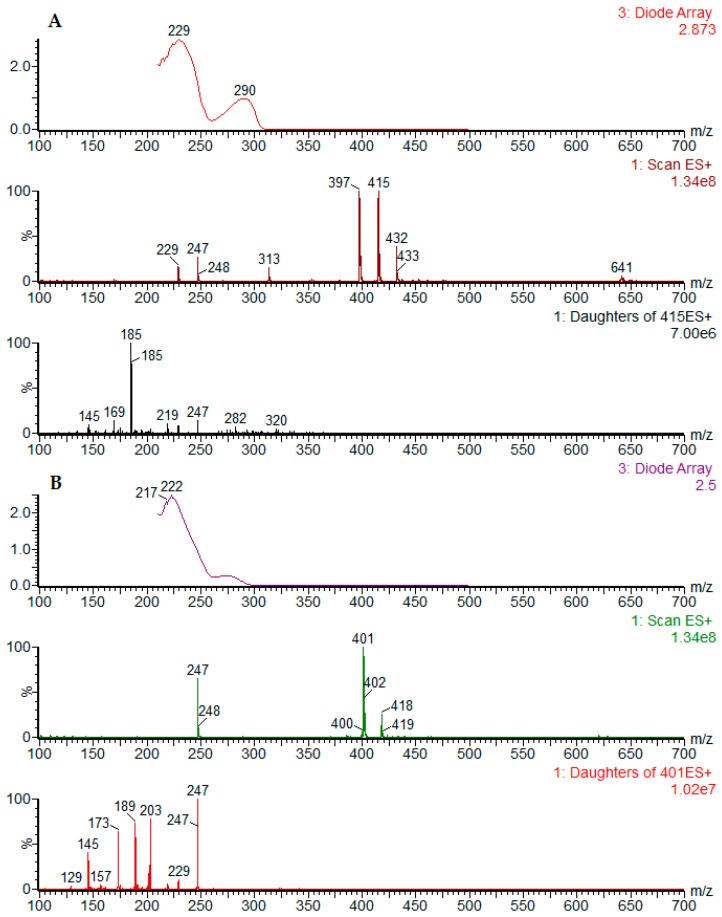
UV, ESI/MS and ESI-MS/MS in the positive ionization mode spectra obtained for podophyllotoxin (**A**) and α-peltatin (**B**).

In this study, the chemical characterization of *D. cymosa* and *P. hexandrum* was performed using UPLC-DAD-ESI-MS. The obtained chromatographic profiles indicated the presence of compounds of different polarities in the EtOH extracts of *D. cymosa* and *P. hexandrum* (Figure 3). The crude extracts as well as lignans previously isolated from *P. hexandrum* such as PTOX (**1**), deoxypodophyllotoxin (**5**), 4′-demethylpodophyllotoxin (**6**), podophyllotoxone (**11**), α-peltatin (**18**) and β-peltatin (**19**) were evaluated in the same chromatographic conditions. The UV and ESI^+^-MS spectra for the reference compounds podophyllotoxin (**1**) and α-peltatin (**18**) are presented in Figure 4 (Figure 4A,B). The UV and MS spectra for all reference compounds and identified chromatographic peaks are available in the Supplementary Material.

The chromatographic profile obtained by UPLC-DAD for the EtOH extracts of *D. cymosa* leaves (Figure 3A), *D. cymosa* roots (Figure 3B) and *P. hexandrum* rhizomes and roots (Figure 3C) showed peaks with UV absorption spectra with λ_max_ around 260–290 nm which is compatible with the chemical structure of lignans due to conjugation of the aromatic rings. It was possible to identify peaks with UV absorption pattern characteristic of aryltetralin lignans related to podophyllotoxin (peaks 4, 8, 11 and 13, λ_max_ at 290 nm) and peltatins (peaks 6 and 10, λ_max_ at 275 nm), and arylnaphtalene lignans, such as diphyllin (peaks 5, 7 and 12; λ_max_ at ca. 260 nm). Peaks with UV spectra characteristic of other phenolic compounds were also identified, such as phenolic acids (peak 1, λ_max_ at 246, 295 and 326 nm) and flavonoids (peaks 2, 3 and 9, λ_max_ at 265 and ca. 350 nm), which are compounds with more conjugated chromophores. 

UPLC-ESI-MS/MS analyses were carried out in order to identify the major constituents of the extracts (Table 1). Chromatographic peak 1 (RT = 1.96 min) showed *m*/*z* at 353 and 355 in the negative and positive ionization modes, respectively. The UV absorption profile of compound **1** was indicative of phenolic acids (λ_max_ ~ 295 and 326 nm). The fragmentation of the parent ion at *m*/*z* 353 in a MS2 experiment in the negative mode afforded daughter ions at *m*/*z* 191 [M − H − caffeoyl]^−^, 179 [M − H − quinic]^−^, and 173 [quinic acid − H − H_2_O]^−^ suggesting the identity of compound **1** as one of the regioisomers of caffeoylquinic acid. The fragmentation pattern of caffeoylquinic acids have been extensively described [33].

The chromatographic peaks 2, 3 and 9 had a UV absorption profile indicative of flavonols, with λ_max_ ~ 255–265 and 350 nm. MS spectra associated to peak 2 (RT = 2.91 min) showed a peak of [M − H]^−^ at *m*/*z* 463 and [M + H]^+^ at *m*/*z* 465. The MS2 fragmentation of the parent ion at at *m*/*z* 465 in the positive mode afforded a daughter ion at *m*/*z* 303 [M + H − hexose]^+^, indicating that peak 2 could correspond to a hexoside of the flavonol quercetin. The observed fragmentation pattern of the ion at *m*/*z* 303 generated a peak at *m*/*z* 165, which is indicative of the presence of a hydroxyl group at C_3_ of flavonols. A similar UV absorption profile was observed for peak 3 (RT = 3.17 min), which showed a MS spectra with a peak of [M − H]^−^ at *m*/*z* 447 and [M + H]^+^ at *m*/*z* 449. The MS2 fragmentation of the parent ions at *m*/*z* 447 and *m*/*z* 449 afforded daughter ions at *m*/*z* 285 [M − H − hexose] and 287 [M + H − hexose] in the negative and positive ionization modes, respectively. The 16 a.m.u. difference observed between peaks 2 and 3, along with the similarity in the UV spectra and in the fragmentation pattern obtained in MS2 experiments, suggest that peak 2 is a hexoside of the flavonol kaempferol. This is the major peak observed in the chromatogram of *D. cymosa* leaves (Figure 3A). The compound corresponding to peak 9 (RT = 4.53 min) showed a UV and MS profiles similar to those observed for peak 3. The MS spectra registered for this compound presented a peak at *m*/*z* 285 corresponding to the deprotonated molecule [M − H]^−^. The fragmentation pattern of the parent ion at *m*/*z* 285 was similar to that observed for peak 3, suggesting that peak 9 corresponds to the aglicone kaempferol. This flavonol is the major constituent of the EtOH extract of *D. cymosa* roots.

Analysis of the UV and MS spectra associated with peaks 4 (RT = 3.73 min) and 8 (RT = 4.46 min) showed λ_max_ at 290 nm, indicative of aryltetralin lignans. MS spectra associated with peaks 4 and 8 showed signals of [M + H]^+^ at *m*/*z* 577 and 415, respectively. The same pattern was observed in the negative ionization mode, with signals of [M − H]^−^ at *m*/*z* 575 and 459 [M − H + formiate]^−^ for peaks 4 and 8, respectively. The difference of 162 Da between the two compounds indicates the presence of a hexose residue. The fragmentation of the parent ion at *m*/*z* 415 in the positive ionization mode generated the daughter ion at *m*/*z* 247 which is compatible with the neutral loss of a trimethoxybenzyl group (C_9_H_12_O_3_). These results, along with the analysis of the isolated lignan podophyllotoxin in the same conditions, allowed the identification of compound **8** as podophyllotoxin (**1**) and compound **4** as an *O*-hexosyl derivative of podophyllotoxin. PTOX (**1**) is the major component of the EtOH extract of *P. hexandrum* rhizomes and roots. On the other hand, this lignan was not found in the extract of *D. cymosa* roots.

Chromatographic peaks 5 (RT = 3.84 min), 7 (RT = 4.11 min) and 12 (RT = 5.52 min) showed UV spectra similar to those observed for arylnaphtalene lignans (λ_max_ at 275 nm). The MS spectra registered for these compounds showed peaks at *m*/*z* 543, for compounds **5** and **7**, and *m*/*z* 379 for compound **12**, corresponding to the deprotonated molecules [M − H]^−^ in the negative mode. The difference of 162 Da between the compound **12** and compounds **5** and **7** indicates the presence of a hexose residue in the latter. The fragmentation of ion at *m*/*z* 379 in the negative mode originated the daughter ions at *m*/*z* 319 and 391. These fragments were reported for the lignan diphyllin [34], which suggest that compound **12** is diphyllin (**24**) while compounds **5** and **7** are *O*-hexosyl derivatives of diphyllin.

In the UPLC profile of the EtOH extract of rhizomes and roots of *P. hexandrum*, the peak eluted at 3.84 min (peak 5′) showed a different UV absorption profile, with λ_max_ at 287 nm, characteristic of aryltetralin lignans. The MS spectra associated with this compound showed a peak at *m*/*z* 399 [M − H]^−^ and 401 [M + H]^+^ in the negative and positive ionization modes, respectively. Analysis of the lignan 4′-demethylpodophyllotoxin (**6**) in the same conditions allowed us to assign this lignan as the compound responsible for peak 5′.

The compound corresponding to peaks 6 (RT = 3.92 min) and 10 (RT = 4.62 min) showed UV absorption pattern similar to what is described for peltatins. The MS spectra associated with chromatographic peak 6 peak showed peaks at *m*/*z* 399 and 401 for the deprotonated and protonated molecules, respectively. The MS2 fragmentation of the parent ion at *m*/*z* 401, in the positive ionization mode, generated the daughter ion at *m*/*z* 247 which is compatible with the neutral loss of a trimethoxybenzyl group (C_9_H_12_O_3_). These results, along with the analysis of the lignan α-peltatin (**18**) in the same conditions, allowed the identification of compound **6** as α-peltatin (**18**). The MS spectra associated with peak 10 showed peaks at *m*/*z* 413 and 415 for the deprotonated and protonated molecules, respectively. The analysis of the isolated lignan β-peltatin (**19**) in the same conditions, allowed the identification of compound **10** as β-peltatin (**14**).

The UV spectra observed for the compound of peak 11 was very similar to that observed for peaks 4 and 8, suggesting that compound **11** (RT = 4.86) is an aryltetralin lignan. The MS spectrum registered for this compound presented peaks at *m*/*z* 411 and 413 in the negative and positive modes, respectively. The MS2 fragmentation of the parent ion at *m*/*z* 413 was very similar to that observed for the lignan podophyllotoxone (**11**) (RT = 5.28 min), suggesting that the compound corresponding to peak 11 could be its isomer isopicropodophyllone (**14**). 

The peak 13 (RT = 5.78), which is only present in the EtOH extract of *P. hexandrum* rhizomes and roots, showed UV spectra compatible with aryltetralin lignans and the MS spectra presented peaks at *m*/*z* 444 [M − H + formiate]^−^ and 399 [M + H]^+^. These results, along with the results obtained for the purified compound allowed the identification of compound **13** as desoxypodophyllotoxin (**5**).

### 2.2. In Silico Prediction of Biological Activity of Lignans 

The Active-IT system was composed, at the time the calculations were run, of 1987 biological activity datasets modeled with SVM and Naïve Bayes machine learning methods. About 1815 datasets were obtained directly from the PubChem Bioassay database and the remaining 172 datasets were obtained from different sources, including the combination of several PubChem datasets. Some of these modeled datasets were reported before, such as AMES [35], AChE [36] and antifungal/antibacterial activities [37], however most remain unpublished. The complete description of all modeled datasets is far beyond the scope of this paper and will not be discussed in detail.

**Figure 5:**
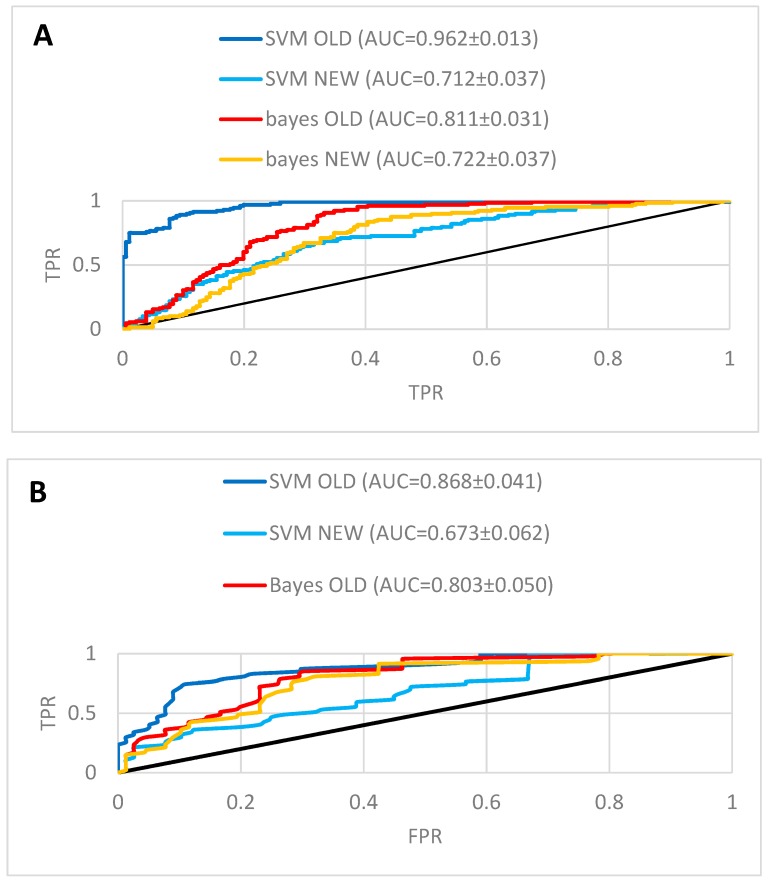
Validation of biological activities prediction of lignans. (**A**) ROC curves obtained in the prediction using all 309 experimental data available for lignans. The values of Pa-Pi scores were used to rank each data point. (**B**) ROC curves obtained for prediction after grouping the experimental data in 125 activity classes (12 classes with an equal number of active and inactive were not considered). The rank was built with an activity class score calculated with the number of lignans with Pa-Pi scores above the threshold, divided by the square root of the number of models belonging to the same class.

The target fishing approach was performed using the Active-IT programme [36]. Before being able to perform predictions of biological activities of the lignans with the Active-IT system, we first made an ultimate validation using some known activities of these compounds. About 12 of the 27 lignans (compounds from **1** to **27**) used in this study appear in one or more datasets from the PubChem Bioassay. Among the 1815 PubChem PubChem Bioassay datasets within the Active-IT system about 243 have one or more lignans of the series, with a total of 309 activity points, with 128 classified as active and 181 classified as inactive. For example, the podophyllotoxin (**1**) appears in 195 different datasets. As expected, the activity predictions of lignans using these models produced excellent results with an AUC of 0.96 for SVM and 0.82 for Naïve Bayes (Figure 5A and Table 2).

Therefore, we decided the best approach for validation was to re-build all of these 243 models, excluding the lignans that appear in each dataset and repeat the prediction. The details of all these models, as well as their internal validation are included in the Supplementary Material (Table S3). The calculated values Pa-Pi of lignans that were excluded from the models are shown in Table S4 of the Supplementary Material. As expected the prediction was a little worse, but the results are still very good, considering most datasets have a cell-based format, with an AUC of 0.71 for SVM and 0.73 for Naïve Bayes (Figure 5A and Table 2). It is worth pointing out that while the SVM method experienced a large decrease in AUC (0.25 AUC units) the Naïve Bayes had a far smaller decrease (0.09 AUC units). This is an evidence that the Naïve Bayes method has a smaller dependence from the input and is less unresponsive to small variations of the dataset composition. The SVM has a lower resilience as it is much more dependent on the input dataset.

However, the validation with AUC only tells us whether the global prediction was accurate or not. In the chemoinformatics it is more important to define whether the most probable active compounds appear in the highest positions in the ranking.

Several metrics can be used to decide the better cutoff to be applied in a classification schema. We used the three metrics based on a contingency matrix to decide the best cutoff to be used:

(1) F-score is a measure of the accuracy of the test, calculated by the harmonic mean of recall or sensitivity [TP/(TP + FN)] and precision [TP/(TP + FP)];
(2) Matthews Correlation Coefficient (MCC) is a balanced measure of the quality of binary classification and is the most informative single score to establish the quality of a binary classifier prediction in a confusion matrix context [38];
(3) Enrichment Factor (EF) is a measure of how many more active compounds we find relative to a random distribution, it is calculated from the proportion of true active compounds selected in relation to the proportion of true active compounds in the entire dataset [39].

The complete results of Pa-Pi values calculated for all lignans, as well the predictions made, are included in the Supplementary material (Table S4). The best cutoff for the classification of lignan compounds as active or inactive was Pa-Pi ≥ 0.15 for both SVM and Naïve Bayes methods, using a positive lower limit of Pa-Pi as an additional filter. In Table 3 are presented a summary of the results when this threshold value is applied. In this table it is possible to note that SVM outperforms Naïve Bayes method for both datasets in the prediction of active compounds, and both methods present a higher specificity as few inactive compounds are misclassified as active (low false positive rate). This draws attention to the fact that the misclassified inactive compounds are almost the same for datasets with (OLD) or without the lignans (NEW) and, despite the fact that there are several active missed, both methods present a good precision as most of the compounds predicted as active are really active. Both methods lost performance when the lignans are deleted from the modeling dataset, but the effect is more pronounced in SVM, as discussed before for AUC results.

The main approach used in this work to make predictions about the biological activities of lignans was the grouping of several models in activity classes and this procedure must be validated as well. The 243 datasets used in the validation were classified in 137 activity classes, with the more populated being the models associated with leukemia cancer with 16 different datasets. From these 137 activity classes only 125 showed a predominance of active (47 cases) or inactive compounds (78 cases), and 12 showed an equal number of active and inactive. Thus, the predominance of active or inactive lead us to classify 47 classes as “probable active” as the lignans are prone to be active and 78 classes as “probable inactive” as the lignans are prone to be inactive.

To make the prediction, all 27 lignans were submitted to 243 models and the activity class score was calculated as described in the Material and Methods section. To build this score, the individual Pa-Pi values were not taken in account, only the number of cases where the value of Pa-Pi is above the threshold and with a positive minimum value.

The best threshold was determined by analyzing the value of MCC for each threshold (Table 3), being Pa-Pi ≥ 0.3 for Naïve Bayes and Pa-Pi ≥ 0.25 for SVM. The score of each activity class, in these cases, was calculated by the number of lignans classified as active among all the models belonging to the same class divided by the number of lignans used in the calculations (27) and the square root of the number of models of this class. The division by the square root of the number of models works like a normalization process because as the number of models in the same class increases more compounds are prone to be predicted as active for this class. From our experience, if we divided by the number of models, those classes with a large number of models are penalized, and if we take only the number of compounds the classes with a large number of models are privileged. The division by square root brings some balance to the prediction.

Using this approach we reconstructed the ROC curves using the scores of activity classes and the results are show in the Figure 5B and Table 3. As we can see the values of AUC metric although a little smaller than those obtained when we use the Pa-Pi scores of each individual compound are high enough to say that this approach sounds viable and allow us to use it to classify the whole set of lignans instead of analyzing a much larger number of results when we consider each compound independently. It is worth noting that all lignans were used in this activity class validation and not only those for which experimental results are available. Another interesting result was that for Naïve Bayes the AUC increases when the data points are grouped into activity classes in relation to the analysis of all experimental data.

A partial list of predicted biological activities as well as the targets associates with the diseases are described in the supplementary material (Table S1 for SVM, Table S2 for Naïve Bayes and Table S3 for results when both methods are taken together). In Table 4 is presented a summary about the activities predicted until the 50 position in the ranking of 753 activity classes using both machine-learning methods (Table S3 of the Supplementary Material).

### 2.3. Antibacterial Activity

Infectious diseases, especially those caused by bacteria, are a major concern in several countries mostly due to antimicrobial resistance, which is a global public health issue that could hold back the control of many bacterial diseases [40]. Therefore, the search for new antimicrobial compounds from natural sources is immensely valuable. The in silico target fishing showed a probable antibacterial activity for aryltetralin lignans, as presented previously.

In our study, the ethanolic extracts from roots of *D. cymosa* and from rhizomes and roots of *P. hexandrum* were the most effective extracts against Gram-positive bacteria, reaching total inhibition of the microbial growth against *B. cereus* and proeminent inhibition rates (58.43 ± 2.7% and 67.56 ± 0.8%, respectively) against *S. aureus* (Table 5). PTOX also presented antibacterial activity against *S. aureus* (53.34 ± 8.8%) and *E. coli* (51.57 ± 9.08%). No antibacterial activity of the extracts and PTOX was observed against the Gram-negative pathogens EHEC, *P. aeruginosa* and *Salmonella* Typhi. 

### 2.4. Anticholinesterasic Activity

We evaluated the AChE inhibitory activity of *D. cymosa* and *P. hexandrum* extracts as well as of PTOX. All extracts showed promising inhibition of AChE activity in the quantitative assay. The EtOH extract of leaves from *D. cymosa* showed the higher percentage of inhibition at 400 μg/mL (Table 6). In the bioautographic assay, the extract of *P. hexandrum* showed a higher intensity of white spots, and this result did not correspond to the quantitative assay. This may have happened due to the possibility of false-positive results when the evaluated extract presents some classes of secondary metabolites, such as tannins and phenolics, which do not directly inhibit the enzyme but can induce enzymatic denaturation [57,58]. On the other hand, the in silico studies did not confirm the AChE inhibition activity (position 577 in 753 models) by the group of 27 lignans investigated in the present study. 

### 2.5. Antioxidant Activity

Although, inhibition of AChE is still considered as the main therapeutic strategy to treat Alzheimer’s disease, other events are implicated in the physiopathology of this disease. The role of oxygen reactive species (ROS) have been extensively investigated. Therefore, we evaluated the antioxidant potential of the EtOH extracts of *D. cymosa* leaves, roots and *P. hexandrum* rhizomes and roots. 

The extract of leaves from *D. cymosa* showed antioxidant activity only in the β-carotene/linoleic acid co-oxidation assay, while the extract of roots showed promising antioxidant activity in all evaluated models. The extract of *P. hexandrum* roots and rhizomes presented significant antioxidant potential in all evaluated models. However, the isolated lignan PTOX (**1**) showed no antioxidant activity (Table 6).

### 2.6. Cytotoxicity

The results of the cytotoxicity assay, performed in THP-1 cells using the SRB method, indicated a moderate toxicity for the extracts of leaves from *D. cymosa* and of roots and rhizomes from *P. hexandrum*, which showed cell viability lower than 80% at 200 and 400 μg/mL, with CC_50_ of 368.0 ± 13.8 and 338.9 ± 15.1 µg/mL (Table 6). On the other hand, the extract of roots from *D. cymosa* was cytotoxic at all tested concentrations, with a CC_50_ value of 100.0 ± 5.3 µg/mL. The lignan PTOX (**1**) showed a CC50 value of 400.0 ± 10.3 µg/mL (Table 6). In silico prediction indicated a small probability of lignans to present THP-1 cytotoxicity (position 316 in 753 activity classes).

## 3. Discussion

A broad range of biological activities have been associated with lignans, thus making them an interesting class of secondary metabolites. Even though lignans are known for their toxicity, other biological activities of lignan-rich plant extracts are worth investigating.

The chromatographic characterization of the phenolic content of the EtOH extracts of *D. cymosa* leaves, *D. cymosa* roots and *P. hexandrum* rhizomes and roots indicated a similar chemical composition for the three extracts. The caffeoylquinic acid was only identified in *D. cymosa* leaves, while PTOX hexoside, deoxypodohyllotoxin (**5**) and 4′-demethylpodohyllotoxin (**6**) were only found in *P. hexandrum*. PTOX was found in the EtOH extracts of *D. cymosa* leaves and *P. hexandrum* rhizomes and roots, being the major constituent of the latter. The EtOH extract of roots from *D. cymosa* showed the best antioxidant potential among the evaluated extracts. These results can be explained by the presence of kaempferol as the major constituent of this extract. Numerous studies have shown that flavonoids, such as quercetin and kaempferol, and their heterosides have a wide range of biological activities, including antioxidant, anti-inflammatory and antimicrobial activities [59,60]. Recently, Wang and co-workers [61] observed a DPPH and ABTS radical scavenging activity for kaempferol as well as an inhibition of concanavalin A (Con A)-induced NO or ROS production in LPS-induced RAW 264.7 macrophage cells [61]. In another study, kaempferol was able to scavenge the superoxide anion, hypochlorous acid, chloramine and nitric oxide [62] as well as showed scavenging ability on superoxide anion produced by electrochemical reduction of oxygen [63].

The lignan content of *Podophyllum* and *Diphylleia* species varies both qualitatively and quantitatively, according to the data previously published. UPLC-DAD-MS methods are largely employed for the identification and quantification of lignans in the aforementioned species [64,65,66,67]. Sharma and Arora identified four aryltetralin lignans in the MeOH extract of rhizomes from *P. hexandrum* [64]. In another study, Sharma and Kumar evaluated the extracts of leaves and roots of *P. hexandrum* obtained from different locations by HPLC-ESI-MS, and found that the podophyllotoxin content was twice as high in the roots in comparison with the content found in the leaves of *P. hexandrum* found in high altitudes [65]. Avula e coworkers evaluated the content of podophyllotoxin (**1**), 4′-demethylpodophyllotoxin (**6**), α-peltatin (**18**) and β-peltatin (**19**) in samples from *P. peltatum.* 4′-demethylpodophyllotoxin (**6**) and α-peltatin (**18**) were the main lignans observed for this species, while the content of PTOX varied from 0.004–0.77% when plants colletcted from various colonies within the same site were evaluated [66]. UPLC-ESI-MS methods can also be employed for the pharmacokinetic studies. The lignans podophyllotoxin (**1**), 4-*epi*-podophyllotoxin (**2**), and 4′-demethylpodophyllotoxin (**6**) were simultaneously evaluated in rat plasma using a UPLC-ESI-MS method after oral administration of the EtOH extract of *Diphylleia sinensis*, 367 mg/kg, to Wistar rats [67]. It is noteworthy to mention that this is the first report of the characterization of flavonoids and caffeoylquinic acid in *D. cymosa*.

Some predictions of biological activities observed in this study were in accordance with the ethnopharmacological uses for both plant species, as well as for the other isolated *podophyllum* lignans. However, some new predict activities such as angiogenesis, osteoporosis, myotonic dystrophy and autoimmune diseases were also observed (Table 4). A reasonable agreement could be noted between predictions made with SVM and Naïve Bayes modeling methods, although the use of both methods can produce results that are more reliable, as indicated in the validation step. Thus, all predictions discussed below will be based in a unique rank of 753 activity classes, where the best rank between both methods and the averaged rank were used to produce the final ordered list. 

The in silico approach showed a high probability of lignans have an anti-inflammatory activity (position 1 in 753 activity classes). This activity has been described before for lignans [11]. The most probable target related with this activity is NF-κB activation. The nuclear factor NF-κB pathway has been considered a classical proinflammatory signaling pathway [68].

The cytotoxic activity of lignans has been much explored, as well as their mechanism of action. The results from in silico prediction showed a low cytotoxicity against THP-1 cells (316/753), which is in accordance with our experimental results. Podophyllotoxin have been evaluated in several models using THP-1 cells, with moderate to low toxicity reported. The effect of podophyllotoxin on IL-1β and TNF expression was evaluated using THP-1 cells at 10 µM and no cytotoxicity was observed [69]. However, according with our calculations, lignans could present cytotoxicity against human lymphoblastic (position 2 in 753 activity classes), isogenic chicken DT 40 (position 19 in 753 activity classes), HEK293 (position 32 in 753 activity classes) and MAGI-CCR5 (position 50 in 753 activity classes) cells. Derivatives have been synthetized [11] to obtain new antitumour compounds. Many targets involved in cancer therapy have also been predicted as potential targets for lignans, such as AP1 endonuclease (position 6 in 753 activity classes), tumor antigen p53 (position 7 in 753 activity classes), GLI family zinc (position 11 in 753 activity classes), RecQ-like DNA helicase 1 (position 14 in 753 activity classes). Other targets can be found in Table 4 and Table S3 of Supplementary Material.

The lignans also showed high probability to be active against *Salmonella typhimurum* (position 3 in 753 activity classes), *Mycobacterium tuberculosis* (position 4 in 753 activity classes), *Staphylococcus aureus* (position 23 in 753 activity classes), *Pseudomonas aeruginosa* (position 18 in 753 activity classes) and *Escherichia coli* (position 52 in 753 activity classes). In this study, all these microorganisms were evaluated, with the exception of *M. tuberculosis*. These predictions are in agreement with ours in vitro results from the antibacterial assay. The activity against *M. tuberculosis* is consistent with the ethnopharmacological use in the Eastern world folk medicine [42].

Regarding the in vitro antibacterial activity, the ethanolic extracts from rhizomes and roots of *P. hexandrum* and from roots of *D. cymosa* were the most effective samples against Gram-positive bacteria. PTOX (1) showed significative activity against *S. aureus* and *E. coli*, while no antibacterial activity was observed against the other Gram-negative pathogens. The antibacterial activity of lignans have already been reported. Nanjundaswamy and coleagues [70] reported a relevant antibacterial activity of two synthetic precursors of PTOX against *E. coli*, *P. aeruginosa* and *Salmonella* Typhi. Other authors have also indicated antibacterial activity of extracts from the rhizomes of *P. hexandrum* [71] and analogues of PTOX against *P. aeruginosa* [72], contrasting the results of the present study. This finding supports the ethnopharmacologial uses of both plant species. No mention of antimicrobial activity from *D. cymosa* was reported so far.

Interestingly, PTOX did not elicit a higher inhibition of the growth of most of the evaluated microorganisms, in comparison with the crude extracts. This is probably due to the synergistic effect of secondary metabolites in the crude extracts.

Considering *S. typhimurum*, one target related to the predicted activity is the *PhoP* regulation. This target is composed by two genes *PhoP* and *PhoQ*, associated with virulence, survival inside the macrophages and defensing resistance of *S. typhimurum* [73]. Against *M. tuberculosis*, the possible target is a transaminase BioA, an enzyme involved in biotin biosynthesis, representing a potential target to develop new antitubercular agents [74]. The putative target involved in the activity against *S. aureus* is the Quorum sensing (QS), defined by Reuter and co-workers [75] as the exchange of chemical signals in bacterial populations, that depends on the bacterial density. QS is responsible for virulence in the clinically relevant bacteria. It has been suggested as a promising target for developing new anti-infective compounds. It was not found a specific target related to *E. coli*.

The extracts of roots from *D. cymosa* and rhizomes and roots of *P. hexandrum* showed the best antioxidant potential, while the extract of leaves from *D. cymosa* showed an anticholinesterasic activity. All the extracts showed moderate toxicity to the THP-1 cells, and the cytotoxic activity observed for the extracts of leaves of *D. cymosa* and rhizomes and roots of *P. hexandrum* were similar to that observed for PTOX (**1**), which did not exhibit antioxidant and anticholinesterase activity. These results indicate that PTOX (**1**) could be the major cytotoxic lignan in these extracts, while other phenolic constituents could be responsible for the antioxidant and anticholinesterase activities observed. However, in the extract of roots of *D. cymosa*, which showed the lowest CC_50_ value, the lignan PTOX (**1**) was not found, indicating that other unidentified minor compound is the responsible for the observed cytotoxicity.

According to the in silico prediction (position 49 in 753 models), the lignans can act DNA damage-inducible transcript 3 protein (C/EBP homologous protein, CHOP) which has been proposed as a target of treatments for some neurodegenerative diseases as Alzheimer’s diseases [52]. Furthermore, according to Naïve Bayes prediction (Table S2 in the Supplementary Material) the lignans can possibly act over the protein Tau indicating a potential application to treat the Alzheimer’s disease and other diseases [76].

Another important effect of lignans is the immunosuppressive activity what can be associated to their uses as therapeutic agents against psoriasis and rheumatoid arthritis, as well to prevent the acute rejection of transplanted organs [77,78]. One putative target that could explain this activity is the sphingosine 1-phosphate receptor 1, predict as potential target for lignans (position 20 in 753 activity class), due to its involvement in immune system modulation [79].

The anti-viral activity of lignans is well known since it was first cited in 1942 as a treatment for veneral wart (*Condyloma acuminatum*), an ailment caused by a papilloma virus [80]. There have been reported effects against HIV, herpes simplex, influenza, vaccinia viruses, and measles [42]. These results also confirm our in silico predictions as the activities against HIV-1 and herpes appear in position 9 and 42, respectively, among 753 activity classes.

The hypolipidemic properties of lignans, as reported by Iwasaki and co-workers [81] are in consonance with our prediction as potential target for these compounds the 1-acylglycerol-3-phosphate *O*-acyltransferase, a protein activator of the lipase Atgl [82], predict in position 41 among 753 activity classes. 

There are no reports in literature of the evaluation of the anticholinesterase activity of Berberidaceae plants or of the lignan PTOX (1), but other lignans have already demonstrated in vitro AChE inhibitory activity [83]. In the study by Hung and co-workers [84], sixteen lignans were isolated from *Schizandra chinensis* and were evaluated for the inhibition of AChE in vitro. Among the evaluated compounds, only five were active with IC_50_ lower than 15 μM. Schisandrin was evaluated in vivo by Itoh et al. [85] and was active at 3 mg/kg. El-Hassan et al. [86] demonstrated that Syringaresinol inhibited AChE in vitro with an IC_50_ value of 200 μg/mL. Still exploring the investigation of AChE inhibition activity by lignans, Salleh et al. [87], isolated five lignans from the stem extract of *Beilschmiedia pulverulenta*, which were evaluated in vitro in the microplate inhibition assay for the AChE, showing have IC_50_ values in the range of 179.8 to 504 μM.

Regarding the antioxidant capacity of these compounds, studies by Wang et al. [88] evaluated the ability of the extracts of *S. chinensis* and *S. sphenanthera* to scavenge the DPPH radical. The authors suggested that variations in lignan content between the extracts lead to different antioxidant activities. The *S. chinensis* species showed higher activity due to the higher content of the lignans Schisandrol A and B and Schinsandrin B.

In a study by Dar et al. [63], the antioxidant capacity of PTOX (**1**) was evaluated in the DPPH sequestration and the TBARS lipid peroxidation assays. In both experiments, PTOX presented IC_50_ value higher than 250 μg/mL. 

These results, along with the data found in literature, indicates that PTOX (**1**) is probably not involved in the antioxidant activity observed for *P. hexandrum* and *D. cymosa* extracts, but other lignans or other phenolic constituents may account for the observed activity.

## 4. Materials and Methods 

### 4.1. Plant Material

The leaves and roots of *D. cymosa* plants were collected at the University of Nottingham (Nottingham, UK) in June, July and August 1995. The authentication of plants was confirmed by Julian MH Shaw (Senior Registrar, Horticultural Taxonomy, Royal Horticultural Society, Wisley, Working, UK). Dried rhizomes and roots of *P. hexandrum* were purchased from United Chemical and Allied Products, Calcutta, India. Both plants were kindly provided by Dr Paul M. Dewick (University of Nottingham, Nottingham, UK). The purified lignans used in this study were isolated and identified in a previous work [24]. 

### 4.2. Preparation of Extracts 

Powdered material (4.8 g) of *D. cymosa* (leaves and roots) and *P. hexandrum* (rhizomes and roots) was extracted with 150 mL ethanol 92.8° by sonication for 10 min at room temperature. The solutions were filtered and the solvent was removed under reduced pressure in a rotary evaporator. The process was repeated three times, and the combined extracts were dried in a water bath (40 °C) to yield the ethanolic crude extracts. The latter were 15.83% yield for leaves and 14.03% for roots of *D. cymosa* respectively, as well as 8.33% for rhizomes and roots of *P. hexandrum*. All samples were stored at 4 °C in amber flasks until required.

### 4.3. Chromatographic Characterization of D. cymosa and P. hexandrum Extracts by UPLC-DAD-ESI-MS/MS

The UPLC-ESI-MS profiles were obtained in a UPLC system coupled with DAD and ESI-TQ-MS detectors. The samples were prepared at 1.0 mg/mL using MeOH, centrifuged (10,000 rpm, 10 min) and then filtered through 0.22 µm PTFE filters. A portion of 3 µL of each sample was injected into the chromatographic system. 

#### 4.3.1. Chromatographic Conditions

The elution was carried out using a gradient elution of 0.1% formic acid in deionized water with (A) and 0.1% formic acid in acetonitrile (B), in a gradient elution from 5 to 36.5% of B in 3.5 min, 36.5–54.5% from 3.5 to 7.5 min, 54.5 to 95% from 7.5 to 9.0 min, with a final isocratic period at 95% B from 9.0 to 10.0 min. The analyses were performed in an Acquity-HSS-ODS (150 × 4.0 mm, 1.8 μm) C-18 column at 40 °C.

#### 4.3.2. Mass Spectrometric Conditions

The mass spectrometer (Waters TQ-XS, Milford, DE, USA) was operated in negative and positive electrospray ionization modes and spectra were recorded by scanning the mass range from *m*/*z* 100 to 1000 in both MS and MS/MS modes. Nitrogen was used as drying, nebulising and collision gas. Drying gas flow rate was 12 L/min. The heated capillary temperature was set at 350 °C and nebulizer pressure at 45 psi. The source parameters such as capillary voltage (VCap), fragmentor, skimmer and octapole voltages were set at 3500 V, 175 V, 65 V and 750 V, respectively. For the MS/MS analysis, a ramp of collision energies, from 15 to 70 eV, was used. The obtained data were processed using the MassLynx (version B 04.00) software (Waters, Milford, DE, USA).

### 4.4. In Silico Studies

#### 4.4.1. In Silico Prediction of Biological Activity of Lignans 

The molecular descriptors used to build the models were the multi-conformational 3-point pharmacophore fingerprints produced by in-house software 3D-Pharma [89]. Each conformation of each compound was treated separately, and its heavy atoms were converted to potential pharmacophore points (PPP) which could be one or more of the following six types: hydrogen bond donor, hydrogen bond acceptor, positively charged, negatively charged, aromatic and lipophilic. For each conformation all combinations of three pharmacophore points in the 3D space (triplets) were calculated to compose a pharmacophore fingerprint. The union of uni-conformational fingerprints produce a unique modal fingerprint for each compound [90] which was used for all subsequent calculations. In the cases where the datasets were obtained from PubChem Bioassay the conformations were downloaded from PubChem Compound [91], in all other cases the conformations were produced with OMEGA software from OpenEye with standard options limited to a maximum of 10 conformations [92,93].

The multi-conformation (modal) pharmacophore fingerprint of active and inactive compounds of each dataset were submitted to the in-house software ExCVBA [94] to build and validate machine learning models using support vector machine (SVM) and Naïve Bayes approaches. Each dataset was used to produce SVM and Naïve Bayes ensemble of models through recurrent stratified random partition of the original dataset to produce a training set composed of 70% of the original dataset and a validation set composed of 30% of the original dataset. This process was repeated at least 30 times and the average scores of each compound over the models in which it appears in the validation set was used to assess the modeling performance with the area under the Receiver Operating Characteristic curve (AUC-ROC), as well for activity prediction of new compounds, as described below.

The calculation of AUC-ROC was performed as defined in Equation (1) with the rank sum of active compounds, which is also called Mann-Whitney U test:

(1) $$AUC=1-\frac{1}{N_{a}}\overset{N_{a}}{\underset{j=1}{\sum }}\frac{\left(r_{j}-j\right)}{N_{i}}$$

where *r_i_* is the rank of the *j*th active, *N_a_*, and *N_i_* are the number of active and inactive compounds, respectively. When ties occur between active and inactive the rank of the active were scaled by interpolation to avoid any bias. The expected standard error of AUC in this paper follows the proposition of Nicholls [95] (Equation (2)), based on Hanley [96] approximation for an ‘typical’ ROC curve:

(2) $$AUC=w\pm t_{95\%}\sqrt{\frac{w^{2}\left(1-w\right)/\left(1+w\right)}{N_{a}}+\frac{w{\left(1-w\right)}^{2}/{\left(2-w\right)}^{2}}{N_{i}}}$$

where *w* is the observed AUC. In the estimation of *t*-statistic at 95% (*t*_95%_) the number of degrees of freedom, ν, follows the proposition of Nicholls [97] (Equation (3)), from the variances of actives and inactives and using the Welch–Satterthwaite formula:

(3) $$\nu_{eff}^{AUC}=\frac{{\left(\frac{AUC}{1+AUC}N_{i}+\frac{1-AUC}{2-AUC}N_{a}\right)}^{2}}{\frac{{\left(\frac{AUC}{1+AUC}N_{i}\right)}^{2}}{N_{a}-1}+\frac{{\left(\frac{1-AUC}{2-AUC}N_{a}\right)}^{2}}{N_{i}-1}}$$

The SVM models were built with LibSVM [98] software with linear kernel option. The cost C, which is a penalty parameter applied to misclassified compounds on the training data, was selected with exponentially growing sequences from 2^−12^ to 2^+6^, by means of a 5-fold cross-validation (CV) using the Power Metric [39,99] at χ = TPR + FPR = 0.5 as an optimization objective metric to assure early recovery of active compounds. The Naïve Bayes model was produced using Perl module from CPAN repository [100] which was incorporated into the ExCVBA software (NEQUIM, Belo Horizonte, Brazil).

In the prediction phase the modal multi-conformational pharmacophore fingerprints of the new compounds were submitted to SVM or Naïve Bayes model ensemble and the average raw scores obtained were converted into probabilities through comparison with the score distribution of active and inactive compounds used to build the models (validation sets only), producing a measure of belonging to these two subsets [101]. 

Considering the SVM or Naïve Bayes score of the new compound as the threshold, the probability of it being active (Pa) is equal to the fraction of active compounds with a worse score (FNR) than the compound under prediction (Equation (4)) and the probability of being inactive (Pi) is equal to the fraction of inactive compounds with a better score (FPR) than the lignan under prediction (Equation (5)), as described elsewhere [36]:

(4) $$Pa=\frac{\mathrm{FN}}{N_{a}}=FNR$$

(5) $$Pi=\frac{\mathrm{FP}}{N_{i}}=FPR$$

where *N_a_* and *N_i_* are the number of active compounds and the number of inactive compounds; FN is the number of active compounds with worse scores than the threshold; and FP is the number of inactive compounds with better scores than the threshold. For each model ensemble, the difference between the Pa and Pi (Pa-Pi) was used to evaluate the potential activity of the modeled compounds. Although the variance of Pa-Pi, as well its limits, can be analytically estimated from the variances of Pa and Pi, as described before [36], in this work we used a new approach, as described below.

The variance of the SVM or Naïve Bayes scores of each compound when it appears in the validation sets and the standard error of the mean (SEM) (Equation (6)) were used to build a better estimation of the limits of the prediction:

(6) $$SEM=\frac{SD}{\sqrt{N}}$$

where SD is the standard deviation, defined as the root mean squared of the variance. Accordingly, the limits of scores, computed at 95% of confidence interval, were estimated by Equation (7):

(7) $$score_{limits}=score_{mean}\pm t_{95\%}*SEM$$

**Figure 6:**
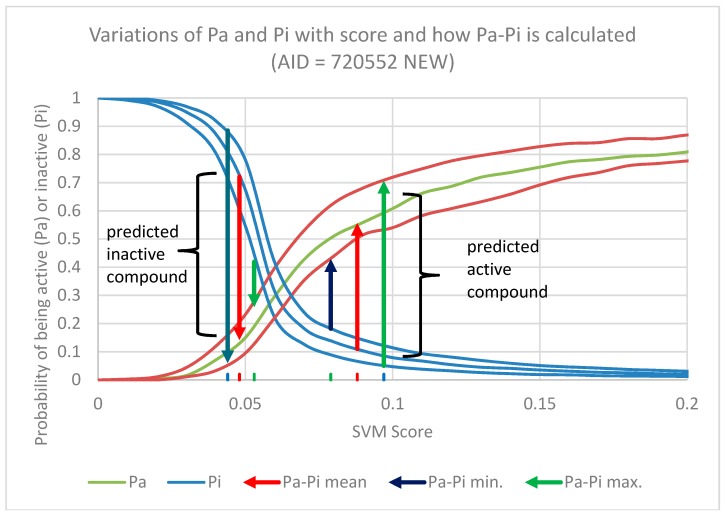
Variation of Pa and Pi probabilities with the SVM score in the model for agonists of the P53 signaling pathway (AID = 720552) together with its upper and lower limits. Calculation of the limits of Pa-Pi for each new compound involve the upper and lower limits of the score over all 30 models of the ensemble. Maximum Pa-Pi will be calculated from the higher value of Pa minus lower value of Pi and minimum Pa-Pi will be calculated from the lower value of Pa minus higher value of Pi. If this value falls below zero the compound is considered inactive.

The values of mean score were used to calculate the mean value of Pa and Pi, while the maximum and minimum values were used to estimate their upper and lower limits, as exemplified in the Figure 6. 

In the prediction phase, the scores of each unseen compound over all 30 models of the ensemble are averaged and the limits are calculated with 95% of confidence interval. The average score is used to calculated the Pa-Pi mean from the average value of Pa minus the average value of Pi. The upper limit of the score is used to calculate the upper limit of Pa-Pi, using the higher value of Pa minus the lowest value of Pi, whereas the lower limit is used to calculate the lowest value of Pa-Pi, using the lower value of Pa minus the higher value of Pi. The limits of Pa-Pi calculated in this way provide a better confidence interval of the prediction, although much larger than the analytical estimate it is more useful as it can be used as an applicability domain approximation, likewise the proposition of Norinder and co-workers [102]. If the new compound looks like an outlier the score variance over all 30 models can be very large and this will reflect in the range of values of Pa-Pi. If the lower value of Pa-Pi falls below zero the compound cannot be predicted as active.

#### 4.4.2. In Silico Prediction of Putative Activity Classes of Lignans

To predict potential biological activities of lignans used in this study, all 1987 datasets of Active-IT system were grouped into 924 activity classes. This grouping approach will make it easier to predict the larger datasets of compounds with a common substructure. To make the prediction of most probable activity classes all data points are filtered with a pre-defined threshold and, additionally, a positive minimum limit of Pa-Pi. In the next step, the instances that pass the filters are counted. The final activity class score is calculated by the sum of instances that pass the filter among all models belonging to the same class, divided by the number of compounds and by the square root of the number of models of the same class.

### 4.5. Evaluation of Antibacterial Activity

Antibacterial susceptibility was performed using the modified microdilution method for bacteria [36,103], against Gram-positive (*Staphylococcus aureus* ATCC 25923 and *Bacillus cereus* ATCC 11778) and Gram-negative recognized pathogens (*Escherichia coli* ATCC 11775, Enterohemorrhagic *E. coli* ATCC 43895-EHEC, *Pseudomonas aeruginosa* ATCC 10145 and *Salmonella choleraesuis* subs. *choleraesuis* sorotype Typhi BM/Panama-TY2). 

The experiments were performed in 96 well microplates. The extracts and isolated substance solubilized in DMSO (Vetec^TM^) at 50 mg/mL were diluted in Mueller Hinton Broth (MHB, Difco) at a concentration of 500 µg/mL. The bacterial isolates, criopreserved at −80 °C were grown in Tryptic Soy Agar (TSA, Acumedia^®^) plates, at 37 °C for 18–24 h. The inoculum was adjusted in saline solution in a spectrophotometer at 625 nm, to a concentration of 1–5 × 108 CFU/mL and then diluted in MHB to 1–5 × 105 CFU/mL. 

Volumes of 100 μL of the inoculum were added to wells containing 100 μL of the extracts at 500 mg/mL, in triplicate (one well intended for extract control), resulting in a final concentration of 250 μg/mL. Chloramphenicol at 50 μg/mL and 0.5% DMSO were used as positive and negative controls, respectively. Evaluation of microbial growth was carried out by adding the inoculum to a well containing only MHB. Sterility of the culture medium was also confirmed by incubation in the assay plate. Assays were performed in triplicate. The microplates were incubated at 37 °C for 24 h. As an indicator of microbial growth, 20 μL of 2,3,5-triphenyltetrazolium chloride (TTC, Sigma-Aldrich, St. Louis, MO, USA) at 5 mg/mLwere added to each well. The plates were incubated at 37 °C for 3 h and then the TTC was solubilized with 100 μL of sodium lauryl sulfate solution in isopropanol 7 μg/mL and measured in a microplate reader at 485 nm. The result was expressed as the percentage of inhibition compared with the microbial growth control [104], and was considered positive for antibacterial activity for those extracts with an inhibition higher than 50%.

### 4.6. Inhibition of Acetylcholinesterase

#### 4.6.1. Bioautographic Assay

The bioautographic assay was performed by thin layer chromatography (TLC) according to the method proposed by Marston, Kissling and Hostettman [105]. The extracts were solubilized in methanol (Merck) at 20 mg/mL, and a portion of 10 μL of this solution was applied on a TLC plate (silica gel 60 F254, Merck, Darmstadt, Germany), which was eluted with CHCl3:MeOH (9:1). The acetylcholinesterase enzyme (*Electropardus electricous* type VI-S, Sigma-Aldrich, St. Louis, MO, USA) solution was prepared by diluting 1 KU of the enzyme in 30 mL of Tris/HCl buffer, pH 7.8, with the addition of 30 mg of bovine serum albumin (BSA). The enzyme solution was sprayed on the TLC plate and incubated in a humid chamber at 37 °C for 20 min. The substrate solution was prepared using 1-naphthyl acetate (2.5 mg/mL) and Fast Blue B salt (2.5 mg/mL), and sprayed on the TLC plate after the incubation period. The purple staining shows the enzymatic activity and the appearance of white bands after 5 min indicates inhibition of AChE activity. The result was expressed by the intensity of the white bands observed.

#### 4.6.2. Microplate Assay

AChE inhibition assay was performed on a 96-well microplate according to Ellman’s method [106] with adaptations. Initially, the extracts were solubilized on MeOH, at 20 mg/mL, and diluted to 4 mg/mL using Tris/HCl buffer solution (50 mM, pH 8.0). A portion of 25 µL of the samples (4 mg/mL) were added to the wells of the microplate, as well as 50 μL of 0.1% BSA solution in Tris/HCl Buffe (pH 8.0), 125 μL of DTNB in Tris/HCl (3 mM) containing NaCl (10 mM) and MgCl2 (20 mM). Subsequently, 25 μL of the ATCI aqueous solution (15 mM) was added to all wells. Then, a background reading was performed at 405 nm using microplate reader (Multiskan Go, Thermo Scientific, Whaltham, MA, USA). The enzymatic reaction was initiated after the addition of 25 µL of AChE solution (AChE from *Electropardus electricous* type VI-S, 0.2 U/mL, Sigma-Aldrich, St. Louis, MO, USA) in Tris/HCl buffer (50 mM, pH 8.0) containing 0.1% BSA. The kinetic cycle was performed in a period of 25 min, with readings at 5 min intervals, at 405 nm. Physostigmine (Eserine, Sigma, St. Louis, MO, USA) was employed as the positive control. A blank was performed in the same assay conditions using MeOH. The assay was performed in triplicate and the % of inhibition (%I) was calculated according to the following equation: %I = [(a − b)/a] × 100, where a = ΔA/min of control; b = ΔA/min of test sample; ΔA = change in absorbance between time *x* and time zero. Extracts with enzyme %I higher than 40% at 400 μg/mL were considered promising.

### 4.7. Evaluation of Antioxidant Activity

#### 4.7.1. β-Carotene/Linoleic Acid Co-Oxidation Assay

The evaluation of the antioxidant activity using the β-carotene/linoleic acid co-oxidation system was performed according to Duarte-Almeida et al. [107], with adaptations. The extracts were solubilized in MeOH (2.2 mg/mL) and further diluted to concentrations ranging from 3.125 to 200 μg/mL. An aliquot of 25 μL of sample solutions were added in the 96-well microplate. 25 mg of linoleic acid and 100 mg Tween 20 (Sigma, St. Louis, MO, USA) were added to a round-bottom flask containing 1 mL of a β-carotene solution (1 mg/mL, Sigma, St. Louis, MO, USA) in chloroform. The latter was removed in a rotatory evaporator and, then, 50 mL of aerated water was added to the flask, affording the β-carotene emulsion. Portions of 250 μL of the freshly prepared emulsion were added to the wells corresponding to the odd columns of a microplate. A blank emulsion was prepared as described above, without the addition of the β-carotene solution, and portions of 250 μL were added to the even columns of the microplate. MeOH and quercetin (20 µg/mL, Sigma, St. Louis, MO, USA) were employed as negative and positive controls, respectively. Readings were performed immediately at 470 nm. After the first reading, the microplate was incubated at 45 °C and the kinetic cycle was performed with readings at 15 min intervals for a total of 120 min. The antioxidant activity was expressed as % of inhibition of lipid peroxidation (%I), using the formula (I% = Ac (initial abs − Final abs) − Aam (final abs − Initial abs)/Ac × 100). Assays were performed in triplicate and IC_50_ values were determined by non-linear regression using GraphPad Prism, version 6.0 (GraphPad Software, San Diego, CA, USA). It was considered promising when the extracts exhibited IC_50_ values lower than 50 µg/mL.

#### 4.7.2. DPPH Radical Scavenger Activity

The evaluation of the antioxidant activity using the 2,2-diphenyl-1-picrylhydrazyl radical (DPPH) was performed according to the method proposed by Mensor et al. [108], with adaptations. The samples were dissolved in MeOH at concentrations ranging from 1.0 to 200 μg/mL. A portion of 250 μL of each sample was added to a 96-well microplate. MeOH and pyrogallol (50 µg/mL) were employed as negative and positive controls, respectively. Then, 100 μL of either MeOH (blank wells) or DPPH solution (120 μg/mL, reaction wells) was added to the microplate. The reading was performed on a microplate reader (EL808IU-Biotek model, Biotek, Winooski, VT, USA), at 5 min intervals for a total of 45 min, at 515 nm. The percentage of radical scavenging activity (%RSA) was calculated using the following equation: % RSA = ((AC − AS)/AC) × 100, where AC is the absorbance of control and AS is the absorbance of samples taken at 35 min. The results were expressed in EC_50_ (effective concentration for 50% capture of the radicals) determined by non-linear regression using GraphPad Prism, version 6.0. In this assay, extracts showing EC_50_ values lower than 15 µg/mL were considered promising.

#### 4.7.3. Thiobarbituric Acid Reactive Substances (TBARS) Assay

The evaluation of antioxidant activity using the TBARS assay [109], with adaptations. Briefly, the samples were solubilized in methanol (50.0 to 31.25 μg/mL) and a portion of 25 μL of each extract solution was added to 10 mL test tubes in triplicate (two reaction tubes and one control). The tubes corresponding to the blank, negative and positive controls were prepared by adding 25 μL of deionized water, MeOH or propylgalate (Sigma, St. Louis, MO, USA, 0.1 mM), respectively. The phospholipid liposomes were prepared using bovine brain extract type VII (Sigma Aldrich, St. Louis, MO, USA, 10 mg/mL) in phosphate buffered saline (PBS) solution. The suspension was subjected to an ultrasonic bath until the formation of the liposomes. Then, 50 μL of the liposome suspension was added to the tubes corresponding to the blank, negative and positive controls, and sample reaction tubes. In sequence, 125 μL of PBS was added to all tubes. The lipid peroxidation was initiated by the addition of 25 μL of FeCl_3_ solution (Vetec, Rio de Janeiro, Brazil, 1 mM) and 25 μL of ascorbic acid solution (Vetec, Rio de Janeiro, Brazil, 1 mM) in the tubes corresponding to the negative control, positive control and sample reaction. The blank tubes were prepared by adding 75 μL of deionized water. All tubes were incubated for 20 min at 37 °C, and after that, 25 μL of BHT (di-tert-butyl methyl phenol, 2% in ethanol), 125 μL of thiobarbituric acid (Merck, Darmstadt, Germany, 1% in 50 mM NaOH), and 125 μL of hydrochloric acid (1%) were added in all tubes. All tubes were shaken vigorously and incubated for 30 min at 80–90 °C. Then, 500 μL of *n*-butanol (Vetec) was added to all tubes which were shaken and placed in the centrifuge for 10 min at 3600 rpm. The butanolic phase was removed and transferred to a 96-well microplate, and the absorbance was recorded on a Multiskan Go microplate reader (Thermo Scientific, Whaltham, MA, USA) at 532 nm. The percentage of inhibition (%I) of lipid peroxidation was calculated as: %I = ((AC − AS)/AC) × 100 where AC is the absorbance of control, AS is the absorbance of samples. Assays were performed in triplicate and IC_50_ values were determined by non-linear regression using GraphPad Prism, version 6.0 (GraphPad Software, San Diego, CA, USA). Extracts showing IC_50_ values lower than 50 µg/mL were considered promising.

### 4.8. Evaluation of Cytotoxicity in THP-1 Cells

The assay was performed following the sulforhodamine B (SRB) method [110]. THP-1 cells (acute human monocytic leukemia cells, ATCC TIB-202) were cultured in a RPMI medium supplemented with 10% fetal bovine serum. A cell suspension was prepared at the density of 1 × 10^6^ cells/mL with 2 μL of PMA (phorbol myristyl acetate, Sigma, St. Louis, MO, USA, 30 µg/mL). A portion of 100 μL of this suspension was transferred to the wells of a microplate. The plate was incubated at 37 °C, in a 5% CO_2_ atmosphere, for about 12 to 16 h to allow cells to adhere. After that, 100 μL of each sample (400, 200, 100, 50 and 25 μg/mL) were added to the wells. The plate was then incubated at 37 °C for 18 h. After incubation, 100 μL of cold trichloroacetic acid (TCA, 10%) solution was added in each well. The plate was incubated at 4 °C for 1 h. The supernatant was discarded and the wells were washed three times with 200 μL of distilled water. Then, the wells were allowed to dry for 24 h. After this period, 100 μL of sulforhodamine B solution (0.057%) was added to each well and the plate was kept at room temperature for 30 min. After that, the wells were washed with 200 μL of 1% acetic acid solution, four times. The plate was allowed to dry for 24 h. In sequence, 100 μL of 10 mM Tris Base solution (pH 10.5) was added to all wells. The plate was then stirred for 5 min inside the microplate reader. The optical density was measured at 510 nm in a microplate reader. A toxicity control of the sample diluent was performed. Cell viability was calculated according to the formula (A − B/C − B) × 100, where A, B and C corresponds to the absorbance measured for the samples, blank and negative control, respectively. The extracts were considered non-toxic when cell viability was higher than 80%.

### 4.9. Statistical Analysis

The results were expressed as mean ± SD of three independent experiments and IC_50_ values were determined by non-linear regression using GraphPad Prism, version 6.0 (GraphPad Software, San Diego, CA, USA). The statistical significance of differences was evaluated using one-way ANOVA in comparison with control groups. Results were considered different when *p* < 0.05. 

## 5. Conclusions

In this study the extracts from the leaves and roots of *D. cymosa* and from the rhizomes and roots of *P. hexandrum* showed antibacterial activity against *B. cereus* and *S. aureus*. On the other hand, podophyllotoxin inhibited the growth of *S. aureus* and *E. coli*. It is important to highlight that the antimicrobial activity of *D. cymosa* was not reported before. *D. cymosa* leaves showed anticholinesterase and antioxidant activities, while the extracts of roots showed antioxidant activities in all evaluated models. The extracts from the rhizomes and roots of *P. hexandrum* presented antioxidant activities in two models used except in the DDPH assay. Additionally, the evaluated extracts from both species were shown to be moderately toxic to THP-1 cells.

According to the chromatographic profiles, the presence of PTOX (**1**), deoxypodophyllotoxin (**5**), 4′-demethylpodophyllotoxin (**6**), podophyllotoxone (**11**), α-peltatin (**18**) and β-peltatin (**19**) were characterized in the EtOH extracts of *D. cymosa* and *P. hexandrum*. These lignans were previously isolated from both Berberidaceae species. Podophyllotoxin (**1**) was the major constituent of *P. hexandrum* extract while kaempferol and its hexoside were the main constituents of *D. cymosa* leaves and roots, respectively. To the best of our knowledge, this is the first report of the characterization of flavonoids and caffeoylquinic acid in *D. cymosa*.

Furthermore, it might be useful to investigate whether *D. cymosa* could be a possible source of podophyllotoxin, and how this could be achieved in the future.

Our Active-IT system proved to be very useful in predicting a broad spectra of biological activities. It was well validated in relation to both the direct score of probabilities to be active or inactive and indirect activity class score. Both approaches produced AUC-ROC values higher than 0.7, even for datasets where no information about lignans was provided. In particular, our in silico studies using machine learning methods was very effective in confirming both ethnopharmacological uses and biological activities of *D. cymosa* and *P. hexandrum* extracts. Moreover, the prediction results suggest that extracts of *D. cymosa* and *P. hexandrum* could provide insights in the research against Alzheimer’s, antimicrobial and anti-inflammatory diseases. In addition, new predicted activities against diseases related to the endocrine system, lipidic disorders, neuropathies, osteoporosis, as well as antiangiogenic should be investigated in the search for new drugs with a clinical use. The aforementioned activities and their associated targets should be more fully explored with the aim of obtaining new uses for known lignans as well as to contributing to the understanding of the mechanism of the actions of these natural compounds from *D. cymosa* and *P. hexandrum*. It would be desirable if the new predicted activities could attract more attention from researchers and students, and hopefully the results would be helpful to the worldwide community.

## Figures and Tables

**Table 1:** Identification of phenolic compounds in the EtOH extrats of *D. cymosa* leaves, *D. cymosa* roots and *P. hexandrum* rhizomes and roots by UPLC-DAD-ESI-MS/MS in negative and positive ionization modes.

Peak No.	Sample	RT (min)	Identity	[M − H]^−^ Parent Ion	[M + H]^+^ Parent Ion	MS2 Fragments Negative Mode (Daughter Ion)	MS2 Fragments Positive Mode (Daughter Ion)	UV (nm)
**1**	*D. cymosa* leaves	1.96	caffeoylquinic acid	353	355	191 [M − H − caffeoyl] 179 [M − H − quinic]	-	217, 246, 295, 326
**2**	*D. cymosa* leaves *D. cymosa* roots *P. hexandrum* rhizomes and roots	2.91	quercetin hexoside	463	465	-	303 [M + H − hexose]	255, 352
**3**	*D. cymosa* leaves *D. cymosa* roots *P. hexandrum* rhizomes and roots	3.17	kaempferol hexoside	447	449	285 [M − H − hexose]	287 pM + H − hexose]	265, 348
**4**	*P. hexandrum* rhizomes and roots	3.73	podophyllotoxin hexoside	575621 [M + formiate]^−^	577	413 [M − H − hexose]	-	290
**5**	*D. cymosa* leaves *D. cymosa* roots	3.84	diphyllin hexoside	-	543	-	381 [M + H − hexose] 363, 333, 319	261
**5**′	*P. hexandrum* rhizomes and roots	3.84	4′-demethylPTOX	399	401	-	-	287
**6**	*D. cymosa* leaves *D. cymosa* roots *P. hexandrum* rhizomes and roots	3.92	α-peltatin	399	401	-	247 [M + H − C_9_H_12_O_3_]	275
**7**	*D. cymosa* leaves *D. cymosa* roots	4.11	diphyllin hexoside	-	543	-	381 [M + H − hexose] 363, 333, 319	261
**8**	*D. cymosa* leaves *P. hexandrum* rhizomes and roots	4.46	podophyllotoxin	459 [M + formiate]^−^	415		247 [M + H − C_9_H_12_O_3_] 185 (247-H_2_O-CO_2_)	290
**9**	*D. cymosa* leaves *D. cymosa* roots *P. hexandrum* rhizomes and roots	4.53	kaempferol	285	-		-	265, 366
**10**	*D. cymosa* roots *P. hexandrum* rhizomes and roots	4.62	β-peltatin	413	415	-	247 [M + H − C_9_H_12_O_3_] 185 (247-H_2_O-CO_2_)	275
**11**	*P. hexandrum* rhizomes and roots	4.86	isopicropodophyllone	411	413	-	-	290
**12**	*D. cymosa* leaves *D. cymosa* roots *P. hexandrum* rhizomes and roots	5.52	diphyllin	379	381	319, 291, 275	363, 333, 319	261
**13**	*P. hexandrum* rhizomes and roots	5.78	deoxypodophyllotoxin	444 [M + formiate]^−^	-		-	291

**Table 2:** Results of the area under the ROC curve (AUC) for biological activity prediction of lignans. Only models with AUC > 0.5 were considered. For activity classes the experimental data points were merged into 125 activity classes. In this case, all 27 lignans have had their activity predicted and were used to build the activity class score.

Method	Dataset	Full Experimental		Activity Classes	
		AUC	Number of Data Points	AUC	Number of Data Points
Naïve Bayes	NEW ^a^	0.730 ± 0.037	292	0.767 ± 0.054	125
Naïve Bayes	OLD ^b^	0.816 ± 0.031	292	0.803 ± 0.050	125
SVM	NEW ^a^	0.710 ± 0.037	305	0.673 ± 0.062	125
SVM	OLD ^b^	0.962 ± 0.014	305	0.868 ± 0.041	125

^a^ Datasets where all lignans were excluded; ^b^ Datasets that include some lignans.

**Table 3:** Validation of biological activity prediction of lignans. For all methods the threshold was defined at Pa-Pi = 0.15. Compounds with Pa-Pi better or equal to the threshold are classified as active and compounds with Pa-Pi below the threshold are classified as inactive. Compounds with a Pa-Pi minimum below zero are classified as inactive, no matter the mean Pa-Pi. In the rows named as “Both” the compound was predicted as active if any method predicted it as active. The metrics F-score, MCC and EF were used to define the best threshold (full data not shown).

Method	Dataset	TP	FP	TN	FN	F-Score	MCC	EF
Naïve Bayes	NEW ^a^	30	17	151	94	0.35	0.19	1.54
SVM	NEW ^a^	46	21	159	79	0.48	0.30	1.66
Both	NEW ^a^	60	33	148	68	0.54	0.31	1.56
Naïve Bayes	OLD ^b^	51	17	151	73	0.53	0.36	1.81
SVM	OLD ^b^	106	18	162	19	0.85	0.75	2.06
Both	OLD ^b^	112	33	148	16	0.82	0.68	1.87

^a^ Datasets where all lignans were excluded; ^b^ Datasets that include some lignans.

**Table 4:** In vitro biological activities of extracts and lignans isolated from *Diphylleia cymosa* and *Podophyllum hexandrum*, ethnopharmacological uses and predicted activities using both machine learning methods.

Studies	Etnopharmacological Uses, Predicted and Observed Biological Activities		Literature
**Ethnopharmacological uses**	*P. hexandrum*: Treatment of diarrhoea and liver problems, to promote conception, eye treatment, chronic constipation, hepatic stimulant, antitumour, purgative, cholagogue and purgative		[11,24]
	*D. cymosa:* Diuretic, antiseptic, diaphoretic and for the treatment of smallpox		[21,24]
**Biological activities reported in the literature**	Antitumour, insecticidal, antimalarial, fungicidal, antiviral, anti-inflammatory, neurotoxic, immunosuppressive, antirheumatic, antispasmogenic and hypolipidemic properties		[41,42]
**Predicted biological activities using both machine learning methods**	ADMET	Cytochrome P450 3A4 (30/753), human intestinal absorption (48/753)	[43]
	Antibacterial	*S. typhimurium* (3/753), *M. tuberculosis* (4/753), *P. aeruginosa* (18/753), *S. aureus* (23/753)	[42]
	Antifungal	*C. albicans* (12/753)	[44]
	Antiparasitic	*Plasmodium falciparum* (5/753), *Trypanosoma* (15/753), *Caenorhabditis elegans* (16/753), *G. lamblia* (44/753)	[45]
	Antitumour	Ape1 Endonuclease (6/753), Agonist of p53 (7/753), GLI family zinc finger 1 (11/753), TOR pathway (13/753), RecQ-Like Dna Helicase 1 (RECQ1) (14/753), Microphthalmia-associated transcription factor (17/753), Hsf1 protein (22/753), serine/threonine-protein kinase 33 (25/753), miR-21 (27/753), sentrin-specific protease 8 (31/753), Acute myelogenous leukemia (35/753), Steroid receptor coactivator 3 (40/753), dual specificity protein phosphatase 3 (43/753), leukemia (45/753)	[41,42]
	Antivirus	HIV-1 (9/753), herpes (42/753)	[46,47,48,49]
	Cytotoxicity and genotoxicity	Lymphoblastoid (2/753), ATAD5 (10/753), isogenic chicken DT40 (19/753), HEK293 (32/753), MAGI-CCR5 (50/753)	[41,42]
	Endocrine disorders	Muscleblind-like protein 1 (26/753), estrogen receptor alpha agonist (34/753), estrogen receptor alpha antagonist (38/753), androgen receptor antagonist (46/753),	[50]
	Lipid disorders	Regulator of G-protein signaling 8 (28/753), 1-acylglycerol- 3-phosphate O-acyltransferase ABHD5 (41/753)	[51,52]
	Neuropathies	Sphingosine 1-phosphate receptor 1 (20/753), regulator of G-protein signaling 4 (33/753), peripheral myelin protein 22 (36/753), Mitochondria permeability (47/753), DNA damage-inducible transcript 3 protein (49/753)	[53]
	Others	Angiogenesis (21/753), osteoporosis (29/753) Anti-Inflammatory (1/753)	[5,40,41,42,54,55,56]

Numbers in parenthesis indicate the rank position among 753 activity classes.

**Table 5:** Antibacterial activity of EtOH extracts (250 µg/mL) from *D. cymosa* and *P. hexandrum* and podophyllotoxin.

Samples	*S. aureus* Mean ± SD	*B. cereus* Mean ± SD	*E. coli* Mean ± SD	EHEC *E. coli* Mean ± SD	*P. aeruginosa* Mean ± SD	*Salmonella* Typhi Mean ± SD
***D. cymosa* (leaves)**	24.76 ± 5.8	0	34.30 ± 2.33	0	23.18 ± 9.94	24.88 ± 3.93
***D. cymosa* (roots)**	67.56 ± 0.8	100.68 ± 0.22	44.68 ± 8.80	16.61 ± 2.13	20.41 ± 8.82	41.33 ± 8.32
***P. hexandrum* (rhizomes and roots)**	58.43 ± 2.7	100.66 ± 0.28	49.28 ± 6.97	18.53 ± 5.21	22.51 ± 15.91	42.52 ± 6.40
**Podophyllotoxin**	53.34 ± 8.8	0	51.57 ± 9.08	33.00 ± 7.76	30.19 ± 9.39	31.62 ± 7.54

**Table 6:** In vitro evaluation of antioxidant and anticholinesterase activity of ethanolic extracts from *D. cymosa*, *P. hexandrum* and podophyllotoxin.

Samples	Antioxidant Activity (IC_50_-µg/mL)			Inhibition of AChE		Cytotoxicity (CC_50_)
	β-Carotene/linoleic Acid	DPPH Radical Sequestration	TBARS Assay	TLC	Microplate (%I)	
***D. cymosa* (leaves)**	19.48 ± 5.90	133.94 ± 25.60	>50	+	64.22 ± 4.87	368.0 ± 13.8
***D. cymosa* (roots)**	20.76 ± 1.76	43.77 ± 6.69	10.20 ± 1.46	-	40.86 ± 3.70	100.0 ± 5.3
***P. hexandrum* (rhizomes and roots)**	30.70 ± 2.12	24.66 ± 4.45	13.66 ± 1.35	++++	47.04 ± 3.17	338.9 ± 15.1
**Podophyllotoxin**	>200	>200	>50	-	32.73 ± 5.38	400 ± 10.3
**Quercetin**	0.3 ± 0.1	NA	NA	NA	NA	NA
**Pyrogallol**	NA	1.14 ± 0.15	NA	NA	NA	NA
**Propylgalate**	NA	NA	<20	NA	NA	NA
**Physostigmine**	NA	NA	NA	++++	89.81 ± 1.16	NA

- not active; + slight inhibition, ++++ strong inhibition.

## Supplementary Materials

The following are available online at http://www.mdpi.com/1420-3049/23/12/3303/s1, Figures S1 and S2: Chromatographic profiles obtained by UPLC for the EtOH extract of leaves (S1) and roots (S2) from *D. cymosa* with detection by DAD and ESI-MS, Figure S3: Chromatographic profiles obtained by UPLC for the EtOH extract of rhizomes and roots from *P. hexandrum*, with detection by DAD and ESI-MS, Figures S4–S22: DAD, ESI-MS and ESI-MS/MS spectra obtained online by UPLC-DAD-ESI-MS/MS for peaks 1-2-3-4-5-5′, 4′-demethylpodophyllotoxin, peak 6, alpha-peltatin, peaks 7–8, podophyllotoxin, peaks 9–10, beta-peltatin, peaks 11–12–13, deoxypodophyllotoxin, respectively; Table S1: Biological activities better classified by the SVM method for the 27 lignans analyzed, Table S2: Biological activities better classified by the Naïve Bayes method for the 27 lignans analyzed, Table S3: Best predict activity classes for lignans using both methods SVM and Naïve Bayes, Table S4: Bioassays used to validate the bioactivity predictions of lignans, Table S5: Validation of lignans bioactivity prediction with new models built without lignans, Table S6: Main targets predicted associated to anti-inflammatory, antibacterial and anti-protozoa activities of lignans, Table S7: Targets related to anti-inflammatory effects, cytotoxicity THP-1, *Salmonella typhimurium*, *Mycobacterium tuberculosis*, *Plasmodium falciparum*, *Pseudomonas aeruginosa* and *Escherichia coli*.
