# Supplementary material for: Combined In Vitro Studies and in Silico Target Fishing for the Evaluation of the Biological Activities of Diphylleia cymosa and Podophyllum hexandrum
Source: Molecules. 2018 Dec 13;23(12):3303. doi: 10.3390/molecules23123303 (PMC6321136; doi:10.3390/molecules23123303)
Supplement: Supplementary file 1 [file molecules-23-03303-s001.zip › molecules-403221-SM - original/Suplementar_information_Tables S6_S7_Targets.pdf]

Table S6. Main targets predicted associated to by anti-inflammatory, antibacterial and anti-protozoa activities of lignans.

Rank	Activity Class	Subdivision	Description	Target	Symbol	#models	#Pairs	Score
1	Bacteria, Salmonella Typhimurium	S. Typh PhoP	The PhoP regulon is a major regulator of virulence in Salmonella that also controls the adaptation to Mg2+-limiting environments. The PhoP system enables Salmonella to determine its presence in an intracellular or extracellular environment, and to promote the expression of genes required for survival within or entry into host cells, respectively.			8	296	1.94
2	Anti-Inflammatory model	NF-kB	NF-kB inhibition. Many cellular pathways leading to activation of NF-kB-family transcription factors have been identified to be participating in host-defense, immunity, inflammation, and cancer.	signal transducer and activator of transcription 1-alpha/beta isoform alpha	STAT1	12	322	1.72
3	Plasmodium falciparum	Plasmodium	inhibitors of proliferation of Plasmodium falciparum			18	312	1.36
4	Mycobacterium tuberculosis	M. tb	Sensitize Mycobacterium Tuberculosis to Beta-lactam Antibiotics			4	143	1.32
5	Anti-Inflammatory model	Toll-like receptor	In atherosclerosis, kidney transplantation and other diseases, inappropriate inflammatory responses contribute to poor patient outcomes. Toll-like receptor (TLR) signaling has been strongly implicated. TLR recognition of microbial components and partner proteins signaling are important elements of the innate immune response	Toll-like receptor	TLR2, TLR6, TLR3, TLR4	4	137	1.27
6	Mycobacterium tuberculosis	M. tb	BioA catalyzes the reversible transamination between KAPA and DAPA in the biotin biosynthetic pathway. BioD catalyzes the irreversible ATP-dependent carbonylation of DAPA to provide dethiobiotin (DTB), and this step drives the BioA reaction forward.	bioA	bioA	4	128	1.19
7	Anti-Inflammatory model	TNF	TNF promotes the inflammatory response, which, in turn, causes many of the clinical problems associated with autoimmune disorders such as rheumatoid arthritis, ankylosing spondylitis, inflammatory bowel disease, psoriasis, hidradenitis suppurativa and refractory asthma. These disorders are sometimes treated by using a TNF inhibitor. A TNF inhibitor is a pharmaceutical drug that suppresses the physiologic response to tumor necrosis factor (TNF), which is part of the inflammatory response.	transcription factor p65 isoform 1	RELA	8	164	1.07
8	Bacteria, Staphylococcus aureus	S. au BQS	Inhibitors of Bacterial Quorum Sensing			2	72	0.94
9	Bacteria, Escherichia coli	E. coli	Several targets			13	175	0.9
10	Anti-Inflammatory model	STAT1	STAT1 inhibition	signal transducer and activator of transcription 1-alpha/beta isoform alpha	STAT1	4	97	0.9
11	Antimicrobial, E. coli	E. coli	Antimicrobial Assay for E. coli BW25113			2	67	0.88

12	Plasmodium berghei	Plasmodium	inhibitors of Plasmodium falciparum Glucose-6-phosphate dehydrogenase	glucose-6-phosphate dehydrogenase-6-phosphogluconolactonase	CAC24715	6	113	0.85
13	Bacteria, Pseudomonas aeruginosa	Pa VIM-2	VIM-2 metallo-beta-lactamase.	Beta lactamase (plasmid)	bla	4	87	0.81
14	Anti-Inflammatory model	STAT3	STAT3 inhibition	STAT3, partial	STAT3	4	79	0.73
15	Mycobacterium tuberculosis	M. tb	Inhibit Mycobacterium Tuberculosis			10	123	0.72
16	Mycobacterium tuberculosis	M. tb	inhibitors of non-replicating M. Tuberculosis			3	62	0.66
17	Plasmodium falciparum	Plasmodium	inhibitors of the Plasmodium falciparum M18 Aspartyl Aminopeptidase (PFM18AAP).	M18 aspartyl aminopeptidase	PfM18AAP	6	86	0.65
18	Bacteria, Staphylococcus aureus	S. au NAD	Therapeutically unexplored target pathway, biosynthesis of an indispensable redox cofactor, nicotinamide adenine dinucleotide (NAD). Targeting of the key essential genes involved in this pathway presents a promising strategy for the development of novel antibiotics.	hypothetical protein SA1422	P65502	1	34	0.63
19	Plasmodium falciparum	Plasmodium	inhibitors of the Plasmodium falciparum M18 Alanyl Aminopeptidase (PfM18AAP)	M18 aspartyl aminopeptidase	PfM18AAP	1	33	0.61
20	Citotoxicidade, THP-1	THP-1	Citotoxicidade, THP-1			7	82	0.57
21	Bacteria, Pseudomonas aeruginosa	Pa PvdQ	Inhibitors of P. aeruginosa PvdQ acylase. Many pathogens such as P. aeruginosa produce siderophores (e.g. pyoverdine) with molecular weights below 1500 Da that bind to iron			2	40	0.52
22	Plasmodium falciparum	Plasmodium	Inhibition of recombinant Plasmodium falciparum MIF expressed in Escherichia coli BL21 (DE3)	Macrophage migration inhibitory factor homolog, putative	MIF	1	28	0.52
23	Plasmodium falciparum	Plasmodium	inhibit dihydroorotate dehydrogenase in Plasmodium falciparum			1	27	0.5
24	Bacteria, Pseudomonas aeruginosa	Pa Elastase	Elastase; Neutral metalloproteinase; PAE; Pseudolysin; Pro-elastase	Elastase	lasB	1	27	0.5
25	Bacteria, Salmonella Typhimurium	S. typh	DSSTox (CPDBAS) Carcinogenic Potency Database SalmonellaMutagenicity			1	27	0.5
26	Bacteria, Pseudomonas aeruginosa	Pa IMP-1	IMP-1metallo-beta-lactamase	metallo-beta-lactamase IMP-1	AAN87168	3	43	0.46
27	Plasmodium falciparum	Plasmodium	inhibitors of Plasmodium falciparum Glucose-6-phosphate dehydrogenase 6-phosphogluconolactonase			1	24	0.44
28	Plasmodium falciparum	Plasmodium	delayed death inhibitors of the malarial parasite plastid, 96 hour incubation			4	48	0.44
29	Plasmodium falciparum	Plasmodium	inhibitors of the Plasmodium falciparum M7 Leucine Aminopeptidase (PfM17LAP)	M17 leucyl aminopeptidase	LAP	2	33	0.43

30	Mycobacterium tuberculosis	M. tb	Identify Non-Covalent Inhibitors of RecA-Intein Splicing Activity	replicative DNA helicase	dnaB	1	21	0.39

Table S7. Targets related to anti-inflammatory effects, cytotoxicity THP-1, *Salmonella* typhimurium, *Mycobacterium tuberculosis*, *Plasmodium falciparum*, *Pseudomonas aeruginosa and Escherichia coli*.

		SVM OK	Bayes OK	SUM	AID	Description	Target	Symbol
Anti-Inflammatory model	Toll-like receptor	9	27	36	1065537	Inhibition of TLR4	Toll-like receptor 4	TLR4
Anti-Inflammatory model	Toll-like receptor	0	10	10	941	Inhibition of TLR4-MyD88. In atherosclerosis, kidney transplantation and other diseases, inappropriate inflammatory responses contribute to poor patient outcomes. Toll-like receptor (TLR) signaling has been strongly implicated. TLR recognition of microbial components and partner proteins signaling are important elements of the innate immune response	toll-like receptor 4	TLR4
Anti-Inflammatory model	Toll-like receptor	27	27	54	1065534	Inhibition of TLR2	Toll-like receptor 2; Toll-like receptor 6	TLR2; TLR6
Anti-Inflammatory model	Toll-like receptor	23	14	37	602277	Modifiers of Toll-like and RIG-like Receptor Signaling-Poly ICStimulus	Toll-like receptor 3	TLR3
Anti-Inflammatory model	Cytoskeleton	0		0	1249	Modifiers Of Cytoskeleton Assembly		
Anti-Inflammatory model	NF-kB	0	14	14	489006	inhibitors of B-cell specific antigen receptor-induced NF-kB activation. Many cellular pathways leading to activation of NF-kB-family transcription factors have been identified to be participating in host-defense, immunity, inflammation, and cancer.		
Anti-Inflammatory model	NF-kB	0	22	22	489004	inhibitors of both B-cell and T-cell specific antigen receptor-induced NF-kB activation. Many cellular pathways leading to activation of NF-kB-family transcription factors have been identified to be participating in host-defense, immunity, inflammation, and cancer.		
Anti-Inflammatory model	NF-kB	27	27	54	435020	inhibitors of T-cell specific antigen receptor-induced NF-kB activation. Many cellular pathways leading to activation of NF-kB-family transcription factors have been identified to be participating in host-defense, immunity, inflammation, and cancer.		
Anti-Inflammatory model	NF-kB	0	25	25	504665	inhibitors of T-cell specific antigen receptor-induced NF-kB activation. Many cellular pathways leading to activation of NF-kB-family transcription factors have been identified to be participating in host-defense, immunity, inflammation, and cancer.		
Anti-Inflammatory model	NF-kB	0	22	22	489033	inhibitors of T-cell specific antigen receptor-induced NF-kB activation. Many cellular pathways leading to activation of NF-kB-family transcription factors have been identified to be participating in host-defense, immunity, inflammation, and cancer.		
Anti-Inflammatory model	NF-kB	0	22	22	489035	inhibitors of T-cell specific antigen receptor-induced NF-kB activation. Many cellular pathways leading to activation of NF-kB-family transcription factors have been identified to be participating in host-defense, immunity, inflammation, and cancer.		
Anti-Inflammatory model	NF-kB	23	6	29	2333	inhibitors of T-cell specific antigen receptor-induced NF-kB activation. The modulation of immune response activity is one of the major goals in the development of novel therapeutics for auto-immune and inflammatory diseases. The innate system resides at the intersection of the pathways of microbial recognition, inflammation, and cell death, thereby offering various therapeutic	nucleotide-binding oligomerization domain-containing protein 1	NOD1

						targets. In this context, NOD1 and NOD2 are of particular interest, since they recognize distinct structures derived from bacterial peptidoglycans and directly activate NF-kB, a central regulator of immune response, inflammation, and apoptosis. Mutations in the NOD1 and NOD2 genes are associated with a number of human inflammatory disorders, including Crohn's disease (CD), Blau syndrome, early-onset sarcoidosis, and atopic diseases, which characteristically cause constitutive NF-kB activation. Chemical inhibitors of NOD1 and NOD2 would provide powerful research tools for elucidating the roles of these proteins in primary cultured cells from humans and in animal models.		
Anti-Inflammatory model	NF-kB	20	22	42	1308	NF-kappaB inhibition.	nuclear factor NF-kappa-B p105 subunit isoform 1	NFKB1
Anti-Inflammatory model	NF-kB	27	23	50	1309	NF-kB activation. Many cellular pathways leading to activation of NF-kB-family transcription factors have been identified to be participating in host-defense, immunity, inflammation, and cancer.	nuclear factor NF-kappa-B p105 subunit isoform 1	NFKB1
Anti-Inflammatory model	NF-kB	1	19	20	1306	NF-kB activation. Many cellular pathways leading to activation of NF-kB-family transcription factors have been identified to be participating in host-defense, immunity, inflammation, and cancer.	signal transducer and activator of transcription 1-alpha/beta isoform alpha	STAT1
Anti-Inflammatory model	NF-kB	0	0	0	1241	NF-kB activation. The pharmacological treatment of neurodegenerative disorders has been a disappointment when compared to the successes obtained in stroke, other neurological diseases like seizures, and in mental health diseases. It has to be said that the pathogenesis of neurodegenerative disorders and their early diagnosis represent a definite obstacle to effective intervention. Nuclear factor kB (NF-kB) is a key cellular signaling factor in the central nervous system. Although NF-kB signaling pathways have been extensively investigated in cancer and in immunological diseases, NF-kB role in the central nervous system physiology and pathology in non inflammatory disorders of the brain is still unclear. NF-kB has an important role as an inhibitor of neuronal apoptosis and at least in this capacity it represents an interesting target to pursue.	NFKB1 protein, partial ; transcription factor p65 isoform 1	NFKB1; RELA
Anti-Inflammatory model	NF-kB	0	22	22	1303	NF-kB inhibition. Many cellular pathways leading to activation of NF-kB-family transcription factors have been identified to be participating in host-defense, immunity, inflammation, and cancer.	signal transducer and activator of transcription 1-alpha/beta isoform alpha	STAT1
Anti-Inflammatory model	STAT1	21	8	29	1262	STAT1 activation	signal transducer and activator of transcription 1-alpha/beta isoform alpha	STAT1
Anti-Inflammatory model	STAT1	0	11	11	1318	STAT1 activation	signal transducer and activator of transcription 1-alpha/beta isoform alpha	STAT1
Anti-Inflammatory model	STAT1	10	22	32	1317	STAT1 inhibition	signal transducer and activator of transcription 1-	STAT1

							alpha/beta isoform alpha	
Anti-Inflammatory model	STAT1	25	0	25	1263	STAT1 inhibition	signal transducer and activator of transcription 1-alpha/beta isoform alpha	STAT1
Anti-Inflammatory model	STAT3	20	25	45	1316	STAT3 activation	STAT3, partial	STAT3
Anti-Inflammatory model	STAT3	0	8	8	1267	STAT3 activation	STAT3, partial	STAT3
Anti-Inflammatory model	STAT3	0		0	1398	STAT3 activation	STAT3, partial	STAT3
Anti-Inflammatory model	STAT3	24	2	26	1265	STAT3 inhibition	STAT3, partial	STAT3
Anti-Inflammatory model	TNF	10	8	18	457	Augmentation of TNFa induced VCAM-1 cell surface expression. TNF promotes the inflammatory response, which, in turn, causes many of the clinical problems associated with autoimmune disorders such as rheumatoid arthritis, ankylosing spondylitis, inflammatory bowel disease, psoriasis, hidradenitis suppurativa and refractory asthma. These disorders are sometimes treated by using a TNF inhibitor. A TNF inhibitor is a pharmaceutical drug that suppresses the physiologic response to tumor necrosis factor (TNF), which is part of the inflammatory response. TNF is involved in autoimmune and immune-mediated disorders such as rheumatoid arthritis, ankylosing spondylitis, inflammatory bowel disease, psoriasis, hidradenitis suppurativa and refractory asthma, so TNF inhibitors may be used in their treatment. The important side effects of TNF inhibitors include lymphomas, infections (especially reactivation of latent tuberculosis), congestive heart failure, demyelinating disease, a lupus-like syndrome, induction of auto-antibodies, injection site reactions, and systemic side effects.[1]		
Anti-Inflammatory model	TNF	1	9	10	1288	Inhibitors of TNF alpha stimulated E Selectin expression. TNF promotes the inflammatory response, which, in turn, causes many of the clinical problems associated with autoimmune disorders such as rheumatoid arthritis, ankylosing spondylitis, inflammatory bowel disease, psoriasis, hidradenitis suppurativa and refractory asthma. These disorders are sometimes treated by using a TNF inhibitor.	selectin E	SELE
Anti-Inflammatory model	TNF	26	23	49	1013	inhibitors of TNF alpha stimulated VCAM1 expression. TNF promotes the inflammatory response, which, in turn, causes many of the clinical problems associated with autoimmune disorders such as rheumatoid arthritis, ankylosing spondylitis, inflammatory bowel disease, psoriasis, hidradenitis suppurativa and refractory asthma. These disorders are sometimes treated by using a TNF inhibitor.		
Anti-Inflammatory model	TNF	9	8	17	895	inhibitors of TNF alpha/NFkB signaling.	NFKB1 protein, partial	NFKB1
Anti-Inflammatory model	TNF	27	1	28	2337	inhibitors of TNFa specific NF-kB induction. TNF promotes the inflammatory response, which, in turn, causes many of the clinical problems associated with autoimmune disorders such as rheumatoid arthritis, ankylosing spondylitis, inflammatory bowel disease, psoriasis, hidradenitis suppurativa and refractory asthma. These disorders are sometimes treated by using a TNF inhibitor.	tumor necrosis factor	TNF

Anti-Inflammatory model	TNF	10	22	32	1852	inhibitors of TNF-a-specific NF-kB induction. TNF promotes the inflammatory response, which, in turn, causes many of the clinical problems associated with autoimmune disorders such as rheumatoid arthritis, ankylosing spondylitis, inflammatory bowel disease, psoriasis, hidradenitis suppurativa and refractory asthma. These disorders are sometimes treated by using a TNF inhibitor.	tumor necrosis factor	TNF
Anti-Inflammatory model	TNF	5	5	10	2485	inhibitors of TNF-a-specific NF-kB induction. TNF promotes the inflammatory response, which, in turn, causes many of the clinical problems associated with autoimmune disorders such as rheumatoid arthritis, ankylosing spondylitis, inflammatory bowel disease, psoriasis, hidradenitis suppurativa and refractory asthma. These disorders are sometimes treated by using a TNF inhibitor.	tumor necrosis factor	TNF
Anti-Inflammatory model	TNF	0		0	438	TNF alpha induced NFkappaB translocation. Many cellular pathways leading to activation of NF-kB-family transcription factors have been identified to be participating in host-defense, immunity, inflammation, and cancer.	transcription factor p65 isoform 1	RELA
Antimicrobial, E. coli	E. coli	21	22	43	638	Antimicrobial Assay for E. coli BW25113 (wild type) - DR		
Antimicrobial, E. coli	E. coli	4	20	24	635	Antimicrobial Assay for E. coli BW25113 (wild type) mutant pool - DR		
antioxidant response element (ARE)	Oxid	0	0	0	651593	Many diseases have some form of oxidative stress injury and ties to inflammation, causing a host of problems for the patient. The antioxidant response element (ARE) plays an important role in alleviating the harmful effects of oxidative stress. The antioxidant response element (ARE) is a transcriptional regulatory element involved in the activation of genes coding for a number of antioxidant proteins and detoxifying enzymes. These enzymes work in concert to protect tissues from oxidative insults and chemical toxicities in human hepatocytes and immune cells. are essential to study the ARE pathway, and eventually to determine whether this pathway does activate genes that could protect against a host of diseases, including cardiovascular diseases, obesity, diabetes, Alzheimer's, and Parkinson's disease.	Nrf2	NFE2L2
antioxidant response element (ARE)	Oxid	0	0	0	651597	Many diseases have some form of oxidative stress injury and ties to inflammation, causing a host of problems for the patient. The antioxidant response element (ARE) plays an important role in alleviating the harmful effects of oxidative stress. The antioxidant response element (ARE) is a transcriptional regulatory element involved in the activation of genes coding for a number of antioxidant proteins and detoxifying enzymes. These enzymes work in concert to protect tissues from oxidative insults and chemical toxicities in human hepatocytes and immune cells. are essential to study the ARE pathway, and eventually to determine whether this pathway does activate genes that could protect against a host of diseases, including cardiovascular diseases, obesity, diabetes, Alzheimer's, and Parkinson's disease.	Nrf2	NFE2L2
antioxidant response element (ARE)	Oxid	5	1	6	493153	Nrf2 is a transcription factor that maintains cellular redox homoestasis and protects cells from xenobiotics [1,2]. Nrf2 binds to the antioxidant response element (ARE) to induce gene expression of a broad spectrum of genes that encode for antioxidants. Hence this Nrf2 pathway provides a first line of defense against stress caused by exposure to radiation, electrophiles, and xenobiotics. In many cancers it has been found that tumor cells have manipulate the Nrf2 pathway for their survival against cytotoxic chemotherapeutics and radiotherapeutic agents. Finding a small molecule that act as an inhibitor of Nrf2 function would represent a novel therapeutic target that could lead to improvement in survival of patients undergoing chemo- and/or radio- therapy.	nuclear factor erythroid 2-related factor 2 isoform 2	NFE2L2
Antioxidante	Oxid	5	11	16	Modelo-Antioxidante			

Bacteria, Pseudomonas aeruginosa	Pa Elastase	0	27	27	468996	Elastase; Neutral metalloproteinase; PAE; Pseudolysin; Pro-elastase	Elastase	lasB
Bacteria, Pseudomonas aeruginosa	Pa IMP-1	17	18	35	2756	IMP-1metallo-beta-lactamase	metallo-beta-lactamase IMP-1	AAN87168
Bacteria, Pseudomonas aeruginosa	Pa IMP-1	0	8	8	1	IMP-1metallo-beta-lactamase		
Bacteria, Pseudomonas aeruginosa	Pa IMP-1	0	0	0	2189	IMP-1metallo-beta-lactamase	metallo-beta-lactamase IMP-1	AAN87168
Bacteria, Pseudomonas aeruginosa	Pa LasB	0	0	0	624096	Inhibitor profiling of the Pseudomonas aeruginosa virulence factor LasB using N-alpha mercaptoamide template-based inhibitors. Bioorg Med Chem Lett. 2009 Nov 1; 19(21):6230-2	metallo beta-lactamase	blaVIM-2
Bacteria, Pseudomonas aeruginosa	Pa PvdQ	8	15	23	493231	Inhibitors of P. aeruginosa PvdQ acylase. Many pathogens such as P. aeruginosa produce siderophores (e.g. pyoverdine) with molecular weights below 1500 Da that bind to iron	protein PvdQ	YP_002440506
Bacteria, Pseudomonas aeruginosa	Pa PvdQ	4	13	17	PvdQ pyoverdine synthesis	Inhibitors of P. aeruginosa PvdQ acylase. Many pathogens such as P. aeruginosa produce siderophores (e.g. pyoverdine) with molecular weights below 1500 Da that bind to iron		
Bacteria, Pseudomonas aeruginosa	Pa TEM-1	3	21	24	2755	TEM-1 serine-beta-lactamase.	Beta lactamase (plasmid)	bla
Bacteria, Pseudomonas aeruginosa	Pa TEM-1	5	0	5	2184	TEM-1 serine-beta-lactamase.	Beta lactamase (plasmid)	bla
Bacteria, Pseudomonas aeruginosa	Pa VIM-2	17	21	38	2	VIM-2 metallo-beta-lactamase.		
Bacteria, Pseudomonas aeruginosa	Pa VIM-3	16	20	36	2754	VIM-2 metallo-beta-lactamase.	metallo beta-lactamase	blaVIM-2
Bacteria, Pseudomonas aeruginosa	Pa VIM-4	13	0	13	2187	VIM-2 metallo-beta-lactamase.	metallo beta-lactamase	blaVIM-2
Bacteria, Pseudomonas aeruginosa	Pa VIM-5	0	0	0	1860	VIM-2 metallo-beta-lactamase.	Beta lactamase (plasmid)	bla
Bacteria, Salmonella Typhimurium	S. typh	27	0	27	1194	DSSTox (CPDBAS) Carcinogenic Potency Database Salmonella Mutagenicity		
Bacteria, Salmonella Typhimurium	S. Typh PhoP	27	27	54	2831	The PhoP regulon is a major regulator of virulence in Salmonella that also controls the adaptation to Mg2+-limiting environments. The PhoP system enables Salmonella to determine its presence in an intracellular or extracellular environment, and to promote the expression of genes required for survival within or entry into host cells, respectively.		
Bacteria, Salmonella Typhimurium	S. Typh PhoP	27	27	54	2834	The PhoP regulon is a major regulator of virulence in Salmonella that also controls the adaptation to Mg2+-limiting environments. The PhoP system enables Salmonella to determine its presence in an intracellular or extracellular		

						environment, and to promote the expression of genes required for survival within or entry into host cells, respectively.		
Bacteria, Salmonella Typhimurium	S. Typh PhoP	27	27	54	2839	The PhoP regulon is a major regulator of virulence in Salmonella that also controls the adaptation to Mg2+-limiting environments. The PhoP system enables Salmonella to determine its presence in an intracellular or extracellular environment, and to promote the expression of genes required for survival within or entry into host cells, respectively.		
Bacteria, Salmonella Typhimurium	S. Typh PhoP	27	25	52	2840	The PhoP regulon is a major regulator of virulence in Salmonella that also controls the adaptation to Mg2+-limiting environments. The PhoP system enables Salmonella to determine its presence in an intracellular or extracellular environment, and to promote the expression of genes required for survival within or entry into host cells, respectively.		
Bacteria, Salmonella Typhimurium	S. Typh PhoP	11	19	30	1981	The PhoP regulon is a major regulator of virulence in Salmonella that also controls the adaptation to Mg2+-limiting environments. The PhoP system enables Salmonella to determine its presence in an intracellular or extracellular environment, and to promote the expression of genes required for survival within or entry into host cells, respectively.		
Bacteria, Salmonella Typhimurium	S. Typh PhoP	9	19	28	2401	The PhoP regulon is a major regulator of virulence in Salmonella that also controls the adaptation to Mg2+-limiting environments. The PhoP system enables Salmonella to determine its presence in an intracellular or extracellular environment, and to promote the expression of genes required for survival within or entry into host cells, respectively.		
Bacteria, Salmonella Typhimurium	S. Typh PhoP	0	13	13	2384	The PhoP regulon is a major regulator of virulence in Salmonella that also controls the adaptation to Mg2+-limiting environments. The PhoP system enables Salmonella to determine its presence in an intracellular or extracellular environment, and to promote the expression of genes required for survival within or entry into host cells, respectively.		
Bacteria, Salmonella Typhimurium	S. Typh PhoP	0	11	11	1985	The PhoP regulon is a major regulator of virulence in Salmonella that also controls the adaptation to Mg2+-limiting environments. The PhoP system enables Salmonella to determine its presence in an intracellular or extracellular environment, and to promote the expression of genes required for survival within or entry into host cells, respectively.		
Bacteria, Staphylococcus aureus	S. au BQS	17	22	39	700	Inhibitors of Bacterial Quorum Sensing		
Bacteria, Staphylococcus aureus	S. au BQS	11	22	33	1014	Inhibitors of Bacterial Quorum Sensing		
Bacteria, Staphylococcus aureus	S. au NAD	12	22	34	624309	Comparative and functional genomics studies identified a therapeutically unexplored target pathway, biosynthesis of an indispensable redox cofactor, nicotinamide adenine dinucleotide (NAD). Targeting of the key essential genes involved in this pathway presents a promising strategy for the development of novel antibiotics. Blocking NAD biosynthesis by inhibition of an essential enzyme, a nicotinic acid mononucleotide adenylyltransferase (NaMNAT) of the NadD family conserved in most bacterial pathogens, leads to growth suppression of Gram-negative and Gram-positive bacteria, thus validating it as a druggable target amenable to inhibition by small molecules.	hypothetical protein SA1422	P65502
Cytotoxicity, THP-1	THP-1	21	27	48	504852	Cytotoxicity, THP-1		
Cytotoxicity, THP-1	THP-1	7	15	22	THP1	Cytotoxicity, THP-1		

Cytotoxicity, THP-1	THP-1	6	4	10	489025	Cytotoxicity, THP-1		
Cytotoxicity, THP-1	THP-1	0	2	2	1117359	Cytotoxicity, THP-1		
Cytotoxicity, THP-1	THP-1	0	0	0	2252	Cytotoxicity, THP-1		
Cytotoxicity, THP-1	THP-1	0	0	0	2253	Cytotoxicity, THP-1		
Cytotoxicity, THP-1	THP-1	0	0	0	504683	Cytotoxicity, THP-1		
Escherichia coli	E. coli	11	22	33	504843	Bacterial Growth Inhibition. E. coli system was designed to identify inhibitors of translocation of fully folded protein through the Twin Arginine Translocation (Tat) system, through induced expression of the Tat machinery and cargo protein. Both inhibitors of the Tat system and to cell growth show a positive result, and decrease the fluorescence signal detected from the engineered fluorescent cargo protein. To separate specific Tat inhibitors from non-specific growth inhibitors, an antibacterial counter screen was performed to identify non-specific bacterial growth inhibitors.		
Escherichia coli	E. coli	2	22	24	1966	Beta-galactosidase	Beta-galactosidase	Q8VNN2
Escherichia coli	E. coli	0	0	0	488956	Cytotoxicity, bacterial viability (counterscreen). nonselective inhibitors of the AddAB helicase-nuclease complex due to bacterial cytotoxicity		
Escherichia coli	E. coli	0	4	4	651983	Cytotoxicity, bacterial viability (counterscreen). nonselective inhibitors of the RecBCD due to bacterial cytotoxicity		
Escherichia coli	E. coli	0	9	9	720486	Counterscreen, E. coli SSB		
Escherichia coli	E. coli	0	1	1	365	E. coli RNase H Inhibition	Ribonuclease HI	rnhA
Escherichia coli	E. coli	0	21	21	488955	exonuclease V (RecBCD complex) [Escherichia coli str. K-12 substr. MG1655]. As designed, compounds that inhibit RecBCD will allow the virus to replicate and block bacterial growth	exonuclease V (RecBCD complex), beta subunit; exonuclease V (RecBCD complex), gamma chain; exonuclease V (RecBCD complex), alpha chain	RECB; RECC; RECD
Escherichia coli	E. coli	0	8	8	651982	exonuclease V (RecBCD complex) [Escherichia coli str. K-12 substr. MG1655]. As designed, compounds that inhibit RecBCD will allow the virus to replicate and block bacterial growth	exonuclease V (RecBCD complex), beta subunit; exonuclease V (RecBCD complex), alpha chain	RECB; RECD
Escherichia coli	E. coli	0	1	1	623921	exonuclease V (RecBCD complex) [Escherichia coli str. K-12 substr. MG1655]. As designed, compounds that inhibit RecBCD will allow the virus to replicate and block bacterial growth	exonuclease V (RecBCD complex), beta subunit; exonuclease V (RecBCD complex), gamma chain	RECB; RECC
Escherichia coli	E. coli	14	22	36	602230	inhibitors of Escherichia coli DNA-binding ATP-dependent protease La (eLon)	DNA-binding ATP-dependent protease La	lon
Escherichia coli	E. coli	22		22	588481	mRNA interferase toxin, antitoxin is MazE [Escherichia coli str. K-12 substr. MG1655]. The goal of the assay is to identify compounds that can disrupt the MazEF TA system and effectively activate the MazF toxin. Compounds with	mRNA interferase toxin, antitoxin is MazE	mazF

						EC50 <= 20uM are desired. The discovery of such compounds would enable the validation of toxin activation as a novel antibacterial strategy, and the compounds themselves would have potential as antimicrobial agents.		
Escherichia coli	E. coli	0	8	8	504941	Screen for inhibitors of E. coli twin-arginine translocation (TAT) system.	TatABCE protein translocation system subunit	tatA
Escherichia coli	E. coli	1	7	8	2320	Shiga toxin (Stx) is released by certain strains of E. coli and is associated with food-borne gastroenteritis. In some patients, especially children, the toxin enters the bloodstream and causes hemolytic uremic syndrome, a condition that results in kidney, heart, and occasionally brain injury.	shiga toxin 1 variant A subunit	BAC78637
Mycobacterium tuberculosis	M. tb	20	27	47	720590	BioA catalyzes the reversible transamination between KAPA and DAPA in the biotin biosynthetic pathway. BioD catalyzes the irreversible ATP-dependent carbonylation of DAPA to provide dethiobiotin (DTB), and this step drives the BioA reaction forward.	bioA	bioA
Mycobacterium tuberculosis	M. tb	2	27	29	743070	BioA catalyzes the reversible transamination between KAPA and DAPA in the biotin biosynthetic pathway. BioD catalyzes the irreversible ATP-dependent carbonylation of DAPA to provide dethiobiotin (DTB), and this step drives the BioA reaction forward.	bioA	bioA
Mycobacterium tuberculosis	M. tb	0	27	27	743073	BioA catalyzes the reversible transamination between KAPA and DAPA in the biotin biosynthetic pathway. BioD catalyzes the irreversible ATP-dependent carbonylation of DAPA to provide dethiobiotin (DTB), and this step drives the BioA reaction forward.	bioA	bioA
Mycobacterium tuberculosis	M. tb	0	25	25	743071	BioA catalyzes the reversible transamination between KAPA and DAPA in the biotin biosynthetic pathway. BioD catalyzes the irreversible ATP-dependent carbonylation of DAPA to provide dethiobiotin (DTB), and this step drives the BioA reaction forward.	bioA	bioA
Mycobacterium tuberculosis	M. tb	14	0	14	492952	Compounds that Modulate Non-Replicating, Drug-tolerant Compounds in Replicating H37Rv TB of Mycobacterium tuberculosis		
Mycobacterium tuberculosis	M. tb	17	5	22	743175	Elucidation of physiology of non-replicating, drug-tolerant Mycobacterium tuberculosis		
Mycobacterium tuberculosis	M. tb	4	11	15	624273	FATTY-ACID-CoA LIGASE FADD28 (FATTY-ACID-CoA SYNTHETASE) (FATTY-ACID-CoA SYNTHASE) [Mycobacterium tuberculosis H37Rv]	FATTY-ACID-CoA LIGASE FADD28 (FATTY-ACID-CoA SYNTHETASE) (FATTY-ACID-CoA SYNTHASE)	fadD28
Mycobacterium tuberculosis	M. tb	0	21	21	449750	Identify Non-Covalent Inhibitors of RecA-Intein Splicing Activity	replicative DNA helicase	dnaB
Mycobacterium Tuberculosis	M. tb	2	6	8	504860	Inhibit Mycobacterium Tuberculosis		
Mycobacterium Tuberculosis	M. tb	2	5	7	504898	Inhibit Mycobacterium Tuberculosis		
Mycobacterium Tuberculosis	M. tb	2	5	7	504903	Inhibit Mycobacterium Tuberculosis		
Mycobacterium Tuberculosis	M. tb	2	2	4	504857	Inhibit Mycobacterium Tuberculosis		
Mycobacterium Tuberculosis	M. tb	2	2	4	504901	Inhibit Mycobacterium Tuberculosis		
Mycobacterium Tuberculosis	M. tb	1	2	3	504897	Inhibit Mycobacterium Tuberculosis		

Mycobacterium tuberculosis	M. tb	0	1	1	1332	Inhibit Mycobacterium Tuberculosis		
Mycobacterium tuberculosis	M. tb	27	8	35	504645	Inhibit Mycobacterium Tuberculosis		
Mycobacterium tuberculosis	M. tb	27	8	35	504646	Inhibit Mycobacterium Tuberculosis		
Mycobacterium tuberculosis	M. tb	14	5	19	449764	Inhibit Mycobacterium Tuberculosis		
Mycobacterium tuberculosis	M. tb	2	0	2	540359	Inhibitors of Mycobacterium tuberculosis UDP-galactopyranose mutase (UGM) enzyme	UDP-galactopyranose mutase	glf
Mycobacterium tuberculosis	M. tb	0	26	26	651617	inhibitors of non-replicating M. tb using log phase replicating mycobacteria		
Mycobacterium tuberculosis	M. tb	7	0	7	435010	Inhibitors of RecA-Intein Splicing Activity	recombinase A	recA
Mycobacterium tuberculosis	M. tb	0	0	0	489010	Inhibitors of RecA-Intein Splicing Activity	recombinase A	recA
Mycobacterium tuberculosis	M. tb	0	0	0	652135	inhibitors of the fructose-bisphosphate aldolase (FBA) of M. tuberculosis	fructose-bisphosphate aldolase	fba
Mycobacterium tuberculosis	M. tb	0	0	0	2761	inhibitors of the membrane-associated serine protease Rv3671c in M.tuberculosis	serine protease	Rv3671c
Mycobacterium tuberculosis	M. tb	1	1	2	PHOSPHOTYROSINE PROTEIN PHOSPHATASE PTPB (PROTEIN-TYROSINE-PHOSPHATASE)	Mycobacterium_tuberculosis-PHOSPHOTYROSINE_PROTEIN_PHOSPHATASE_PTPB_(PROTEIN-TYROSINE-PHOSPHATASE)		
Mycobacterium tuberculosis	M. tb	27	27	54	504703	Sensitize Mycobacterium Tuberculosis to Beta-lactam Antibiotics		
Mycobacterium tuberculosis	M. tb	26	27	53	504702	Sensitize Mycobacterium Tuberculosis to Beta-lactam Antibiotics		
Mycobacterium tuberculosis	M. tb	13	16	29	434987	Sensitize Mycobacterium Tuberculosis to Beta-lactam Antibiotics		
Mycobacterium tuberculosis	M. tb	0	7	7	493013	Sensitize Mycobacterium Tuberculosis to Beta-lactam Antibiotics		
Plasmodium berghei	Plasmodium	16	22	38	540269	inhibitors of Plasmodium falciparum Glucose-6-phosphate dehydrogenase	glucose-6-phosphate dehydrogenase-6-phospho-gluconolactonase	CAC24715
Plasmodium berghei	Plasmodium	14	22	36	504753	inhibitors of Plasmodium falciparum Glucose-6-phosphate dehydrogenase	glucose-6-phosphate dehydrogenase-6-phospho-gluconolactonase	CAC24715
Plasmodium berghei	Plasmodium	14	9	23	540252	inhibitors of Plasmodium falciparum Glucose-6-phosphate dehydrogenase	glucose-6-phosphate dehydrogenase-6-phospho-gluconolactonase	CAC24715
Plasmodium berghei	Plasmodium	0	16	16	504765	inhibitors of Plasmodium falciparum Glucose-6-phosphate dehydrogenase	glucose-6-phosphate dehydrogenase-6-phospho-gluconolactonase	CAC24715

Plasmodium berghei	Plasmodium	0	0	0	588415	inhibitors of Plasmodium falciparum Glucose-6-phosphate dehydrogenase	glucose-6-phosphate dehydrogenase-6-phospho-gluconolactonase	CAC24715
Plasmodium berghei	Plasmodium	0	0	0	588593	inhibitors of Plasmodium falciparum Glucose-6-phosphate dehydrogenase	glucose-6-phosphate dehydrogenase-6-phospho-gluconolactonase	CAC24715
Plasmodium falciparum	Plasmodium	0	5	5	1159586	Biochemical screen of P. falciparum PK6	protein kinase 6	PfPK6
Plasmodium falciparum	Plasmodium	12	2	14	488752	delayed death inhibitors of the malarial parasite plastid, 48 hour incubation		
Plasmodium falciparum	Plasmodium	4	1	5	504850	delayed death inhibitors of the malarial parasite plastid, 48 hour incubation		
Plasmodium falciparum	Plasmodium	11	5	16	504848	delayed death inhibitors of the malarial parasite plastid, 96 hour incubation		
Plasmodium falciparum	Plasmodium	11	2	13	488745	delayed death inhibitors of the malarial parasite plastid, 96 hour incubation		
Plasmodium falciparum	Plasmodium	0	1	1	743244	Gametocytocidal Compounds. To be transmitted from person to person via a mosquito, the parasites must switch from asexual to sexual development and produce male and female gametocytes. Malaria gametocytes consist of five stages (I, II, III, IV and V). The late stage gametocytes (III, IV and V) are difficult to be cultured in vitro and the long lifespan of mature gametocytes allows them being transmitted from host to mosquitoes unless they are completely eliminated by gametocytocidal agents.		
Plasmodium falciparum	Plasmodium	1	0	1	1154	identify antagonists of the plasmodial surface anion channel (PSAC)		
Plasmodium falciparum	Plasmodium	0	0	0	1155	identify antagonists of the plasmodial surface anion channel (PSAC)		
Plasmodium falciparum	Plasmodium	0	0	0	1157	identify antagonists of the plasmodial surface anion channel (PSAC)		
Plasmodium falciparum	Plasmodium	25	2	27	1175	inhibit dihydroorotate dehydrogenase in Plasmodium falciparum		
Plasmodium falciparum	Plasmodium	2	2	4	540271	inhibiting malaria HSP40-mediated yeast toxicity. During its life cycle in its human host P. falciparum infects and remodels red blood cells. Strikingly, P. falciparum also shows a marked expansion of the heat shock protein 40 (Hsp40) family of co-chaperones and it has been proposed that during this process the parasite relies on a greatly expanded class of Hsp40 co-chaperones to infect and kill cells. In this screening project, the capacity of small molecules to block malaria HSP40-mediated yeast toxicity and induce yeast growth will be measured through luminescence mediated by the presence of intracellular ATP in yeast as an indicator of cell viability.	HSP40, subfamily A, putative	PF14_0359
Plasmodium falciparum	Plasmodium	0	0	0	1159585	inhibition of calcium-dependent protein kinase 1 from Plasmodium falciparum	Calcium-dependent protein kinase 1	CDPK1
Plasmodium falciparum	Plasmodium	0	1	1	1159588	inhibition of calcium-dependent protein kinase 4 from Plasmodium falciparum	calcium-dependent protein kinase 4	PfCDPK4
Plasmodium falciparum	Plasmodium	0	27	27	2302	Inhibition of P. falciparum Dd2	lactate dehydrogenase	ABH03417
Plasmodium falciparum	Plasmodium	13	15	28	711030	Inhibition of recombinant Plasmodium falciparum MIF expressed in Escherichia coli BL21 (DE3)	Macrophage migration inhibitory	MIF

							factor homolog, putative	
Plasmodium falciparum	Plasmodium	6	18	24	Plasmodium falciparum-glucose-6-phosphate dehydrogenase-6-phospho-gluconolactonase	inhibitors of Plasmodium falciparum Glucose-6-phosphate dehydrogenase 6-phosphogluconolactonase		
Plasmodium falciparum	Plasmodium	14	10	24	1828	Inhibitors of Plasmodium falciparum proliferation. This summary is written for the purposes of summarizing the status of the profiling of the Plasmodium falciparum strains. The average PubChem activity score from different strains is used for each compound.		
Plasmodium falciparum	Plasmodium	27	12	39	1876	inhibitors of proliferation of Plasmodium falciparum line 3D7		
Plasmodium falciparum	Plasmodium	0	1	1	449703	inhibitors of proliferation of Plasmodium falciparum line 3D7		
Plasmodium falciparum	Plasmodium	13	13	26	1815	inhibitors of proliferation of Plasmodium falciparum line 7G8		
Plasmodium falciparum	Plasmodium	0	1	1	504316	inhibitors of proliferation of Plasmodium falciparum line 7G8		
Plasmodium falciparum	Plasmodium	0	1	1	504320	inhibitors of proliferation of Plasmodium falciparum line CP250		
Plasmodium falciparum	Plasmodium	11	4	15	1877	inhibitors of proliferation of Plasmodium falciparum line D10		
Plasmodium falciparum	Plasmodium	12	2	14	1882	inhibitors of proliferation of Plasmodium falciparum line Dd2		
Plasmodium falciparum	Plasmodium	0	2	2	504314	inhibitors of proliferation of Plasmodium falciparum line Dd2		
Plasmodium falciparum	Plasmodium	0	1	1	1159567	inhibitors of proliferation of Plasmodium falciparum line Dd2		
Plasmodium falciparum	Plasmodium	27	15	42	1816	inhibitors of proliferation of Plasmodium falciparum line GB4		
Plasmodium falciparum	Plasmodium	0	3	3	504315	inhibitors of proliferation of Plasmodium falciparum line GB4		
Plasmodium falciparum	Plasmodium	27	12	39	1886	inhibitors of proliferation of Plasmodium falciparum line HB3		
Plasmodium falciparum	Plasmodium	2	0	2	504318	inhibitors of proliferation of Plasmodium falciparum line HB3		
Plasmodium falciparum	Plasmodium	0		0	743327	inhibitors of proliferation of Plasmodium falciparum line HB3		
Plasmodium falciparum	Plasmodium	27	27	54	1883	inhibitors of proliferation of Plasmodium falciparum line W2		
Plasmodium falciparum	Plasmodium	0	21	21	449704	inhibitors of proliferation of Plasmodium falciparum line W2		
Plasmodium falciparum	Plasmodium	0	0	0	743274	inhibitors of the Plasmodium falciparum M17 Leucine Aminopeptidase (M17LAP)		
Plasmodium falciparum	Plasmodium	6	11	17	Plasmodium falciparum-M17 leucyl aminopeptidase	inhibitors of the Plasmodium falciparum M17 Leucine Aminopeptidase (PFM17LAP)		
Plasmodium falciparum	Plasmodium	0	3	3	624175	inhibitors of the Plasmodium falciparum M17 Leucine Aminopeptidase (PFM17LAP)		

Plasmodium falciparum	Plasmodium	0	2	2	588679	inhibitors of the Plasmodium falciparum M17 Leucine Aminopeptidase (PFM17LAP)	M17 leucyl aminopeptidase	LAP
Plasmodium falciparum	Plasmodium	11	22	33	492975	inhibitors of the Plasmodium falciparum M18 Alanyl Aminopeptidase (PfM18AAP)	M18 aspartyl aminopeptidase	PfM18AAP
Plasmodium falciparum	Plasmodium	0	7	7	624177	inhibitors of the Plasmodium falciparum M18 Aspartyl Aminopeptidase (PFM18AAP)		
Plasmodium falciparum	Plasmodium	0	2	2	588678	inhibitors of the Plasmodium falciparum M18 Aspartyl Aminopeptidase (PFM18AAP)	M18 aspartyl aminopeptidase	PfM18AAP
Plasmodium falciparum	Plasmodium	0	1	1	720736	inhibitors of the Plasmodium falciparum M18 Aspartyl Aminopeptidase (PFM18AAP)	M18 aspartyl aminopeptidase	
Plasmodium falciparum	Plasmodium	9	20	29	Plasmodium falciparum-M18 aspartyl aminopeptidase	inhibitors of the Plasmodium falciparum M18 Aspartyl Aminopeptidase (PFM18AAP).		
Plasmodium falciparum	Plasmodium	6	19	25	2170	inhibitors of the Plasmodium falciparum M18 Aspartyl Aminopeptidase (PFM18AAP).	M18 aspartyl aminopeptidase	PfM18AAP
Plasmodium falciparum	Plasmodium	8	14	22	492974	inhibitors of the Plasmodium falciparum M18 Aspartyl Aminopeptidase (PFM18AAP).	M18 aspartyl aminopeptidase	PfM18AAP
Plasmodium falciparum	Plasmodium	0	7	7	624176	inhibitors of the Plasmodium falciparum M1AAP (PFM1AAP)		
Plasmodium falciparum	Plasmodium	0	2	2	588680	inhibitors of the Plasmodium falciparum M1AAP (PFM1AAP)	M1-family alanyl aminopeptidase	MAL13P1.56
Plasmodium falciparum	Plasmodium	2	17	19	492973	inhibitors of the Plasmodium falciparum M7 Leucine Aminopeptidase (PfM17LAP)	M17 leucyl aminopeptidase	LAP
Plasmodium falciparum	Plasmodium	1	13	14	492977	inhibitors of the Plasmodium falciparum M7 Leucine Aminopeptidase (PfM17LAP)	M17 leucyl aminopeptidase	LAP
Plasmodium falciparum	Plasmodium	0	2	2	Plasmodium falciparum-m1-family aminopeptidase	inhibitors of the Plasmodium_falciparum-m1-family_aminopeptidase		
Plasmodium falciparum	Plasmodium	0	0	0	652047	inhibitory activity of small molecule on Plasmodium flaciparum (3D7 strain) survival		
Plasmodium falciparum	Plasmodium	1	0	1	652041	inhibitory activity of small molecule on Plasmodium flaciparum (HB3 strain) survival		
Plasmodium falciparum	Plasmodium	0	8	8	602156	Liver Stage Dataset: Malariabox Annotation. Most malaria drug development focuses on parasite stages detected in red-blood cells even though to achieve eradication next-generation drugs active against both erythrocytic and exo-erythrocytic forms would be preferable.		
Plasmodium falciparum	Plasmodium	0	7	7	602118	Liver Stage Dataset: Malariabox Annotation. Most malaria drug development focuses on parasite stages detected in red-blood cells even though to achieve eradication next-generation drugs active against both erythrocytic and exo-erythrocytic forms would be preferable.		
Plasmodium falciparum	Plasmodium	6	14	20	Plasmodium falciparum-Glutathione metabolism (Plasmodium falciparum 3D7)	Plasmodium_falciparum-Glutathione_metabolism_(Plasmodium_falciparum_3D7)		
