# Supplementary material for: Combined In Vitro Studies and in Silico Target Fishing for the Evaluation of the Biological Activities of Diphylleia cymosa and Podophyllum hexandrum
Source: Molecules. 2018 Dec 13;23(12):3303. doi: 10.3390/molecules23123303 (PMC6321136; doi:10.3390/molecules23123303)
Supplement: Supplementary file 1 [file molecules-23-03303-s001.zip › molecules-403221-SM - original/Supplementar_information_LC_MS.pdf]

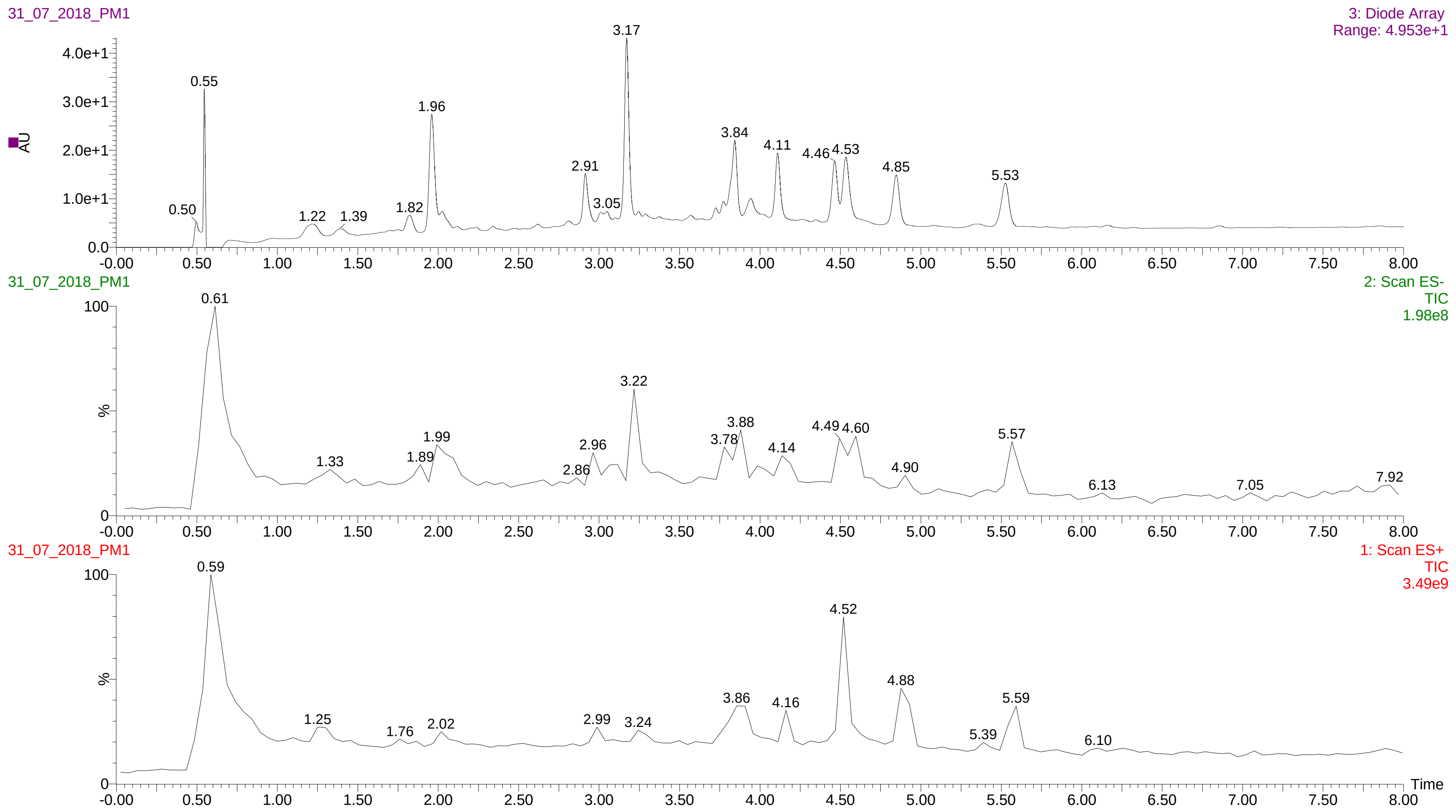

Figure S1. Chromatographic profiles obtained by UPLC for the EtOH extract of leaves from *D. cymosa* with detection by DAD and ESI-MS in the negative and positive ionization modes.

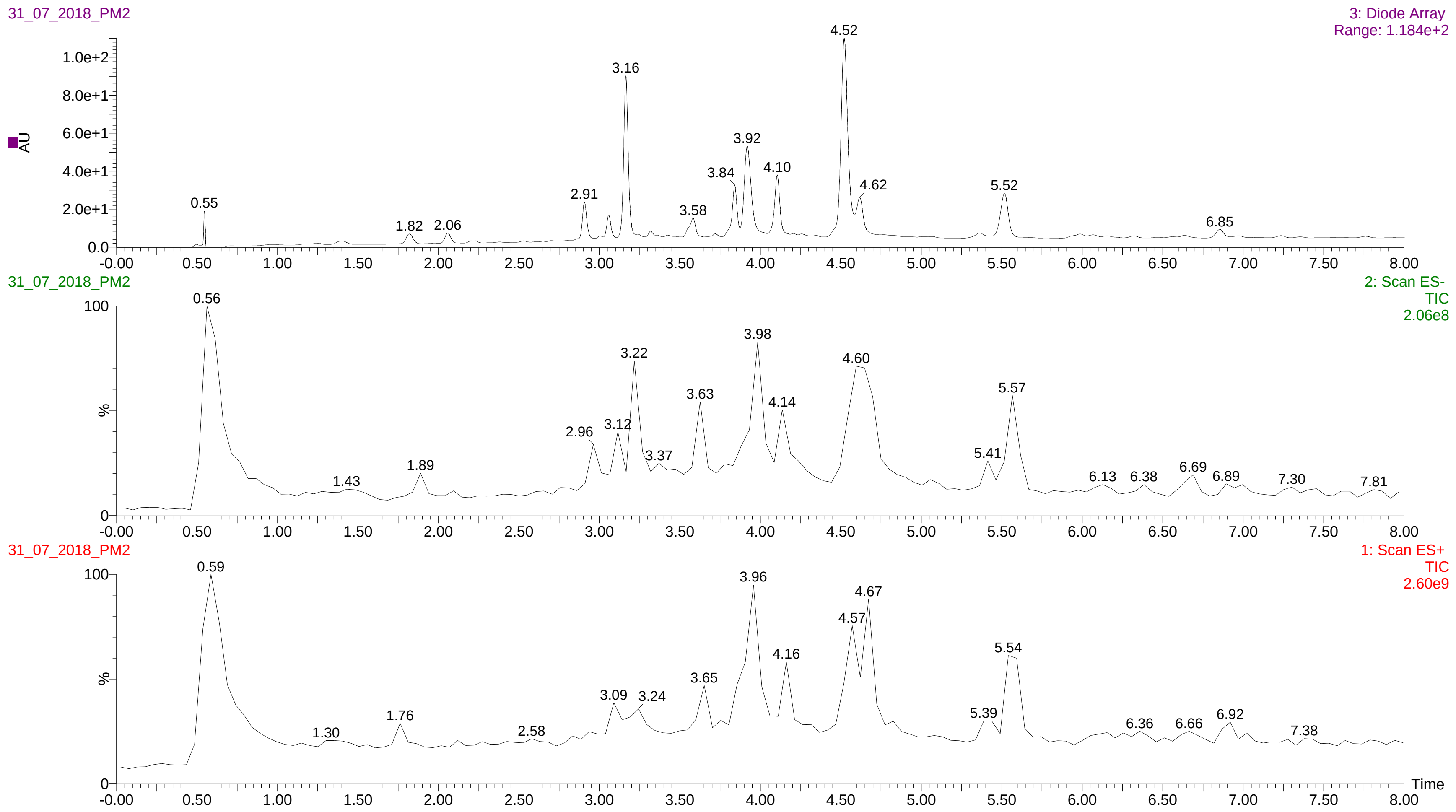

Figure S2. Chromatographic profiles obtained by UPLC for the EtOH extract of roots from *D. cymosa* with detection by DAD and ESI-MS in the negative and positive ionization modes.

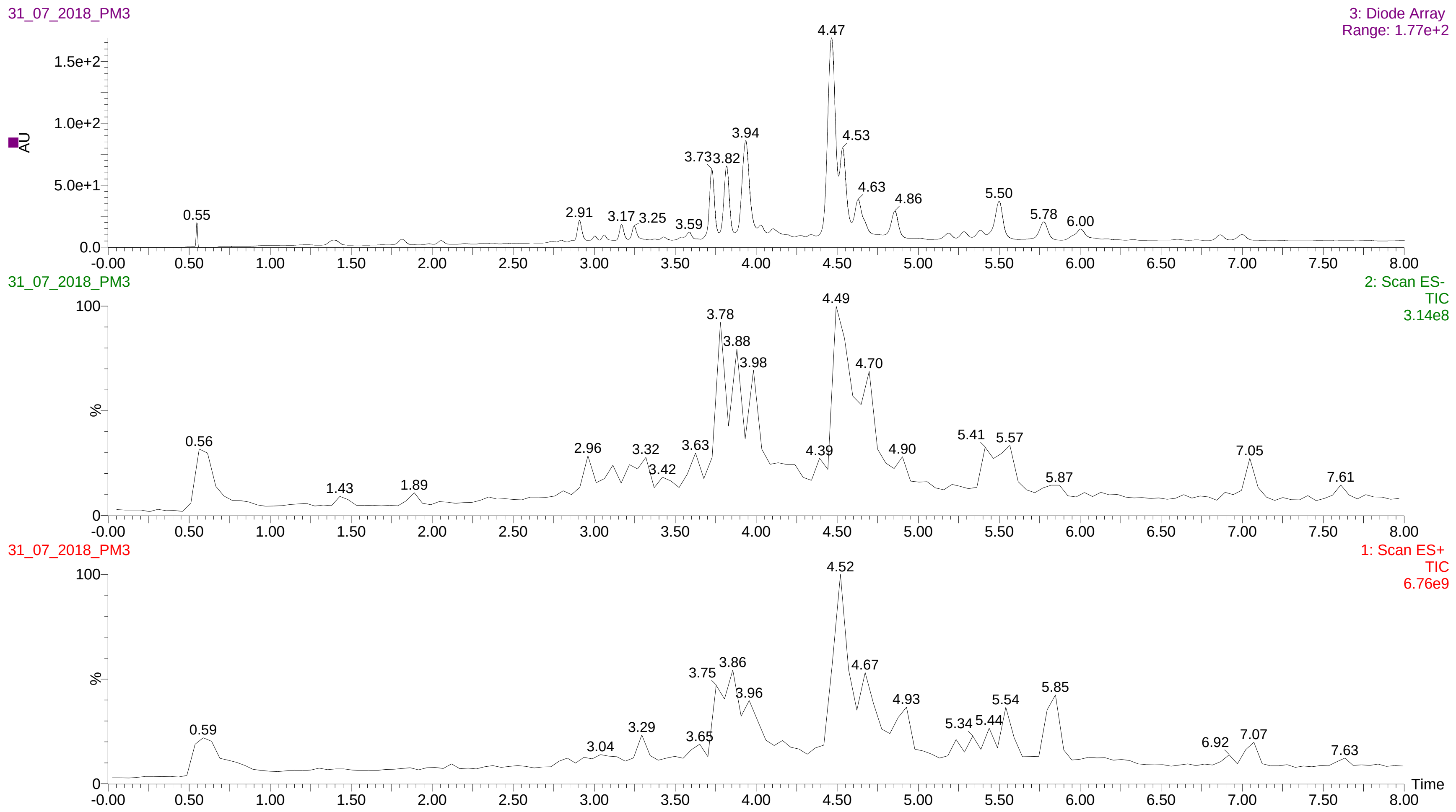

Figure S3. Chromatographic profiles obtained by UPLC for the EtOH extract of rhizomes and roots from *P. hexandrum* with detection by DAD and ESI-MS in the negative and positive ionization modes.

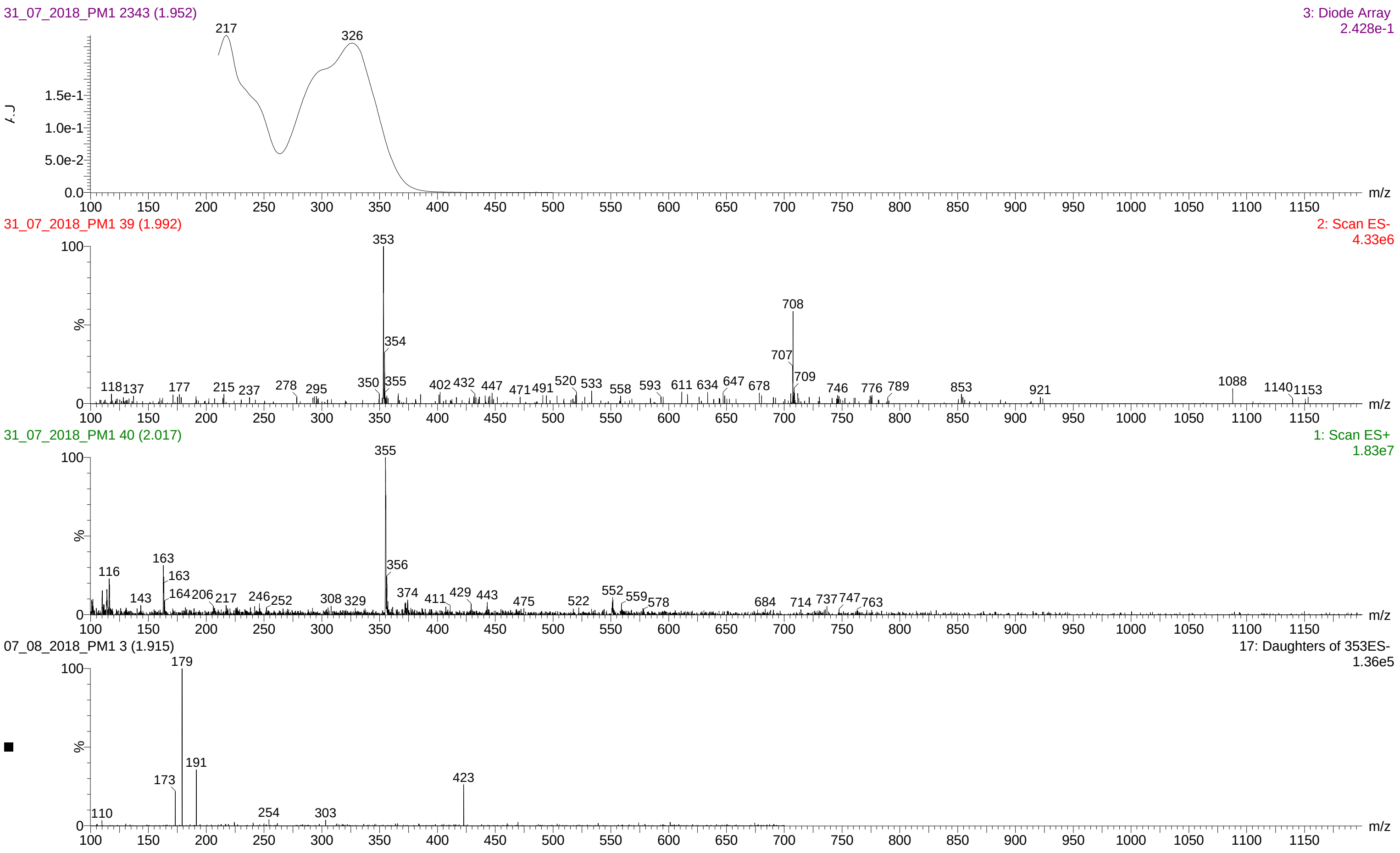

Figure S4. DAD, ESI-MS in the negative and positive ionization modes, and ESI-MS/MS spectra obtained online by UPLC-DAD-ESI-MS/MS for chromatographic peak 1.

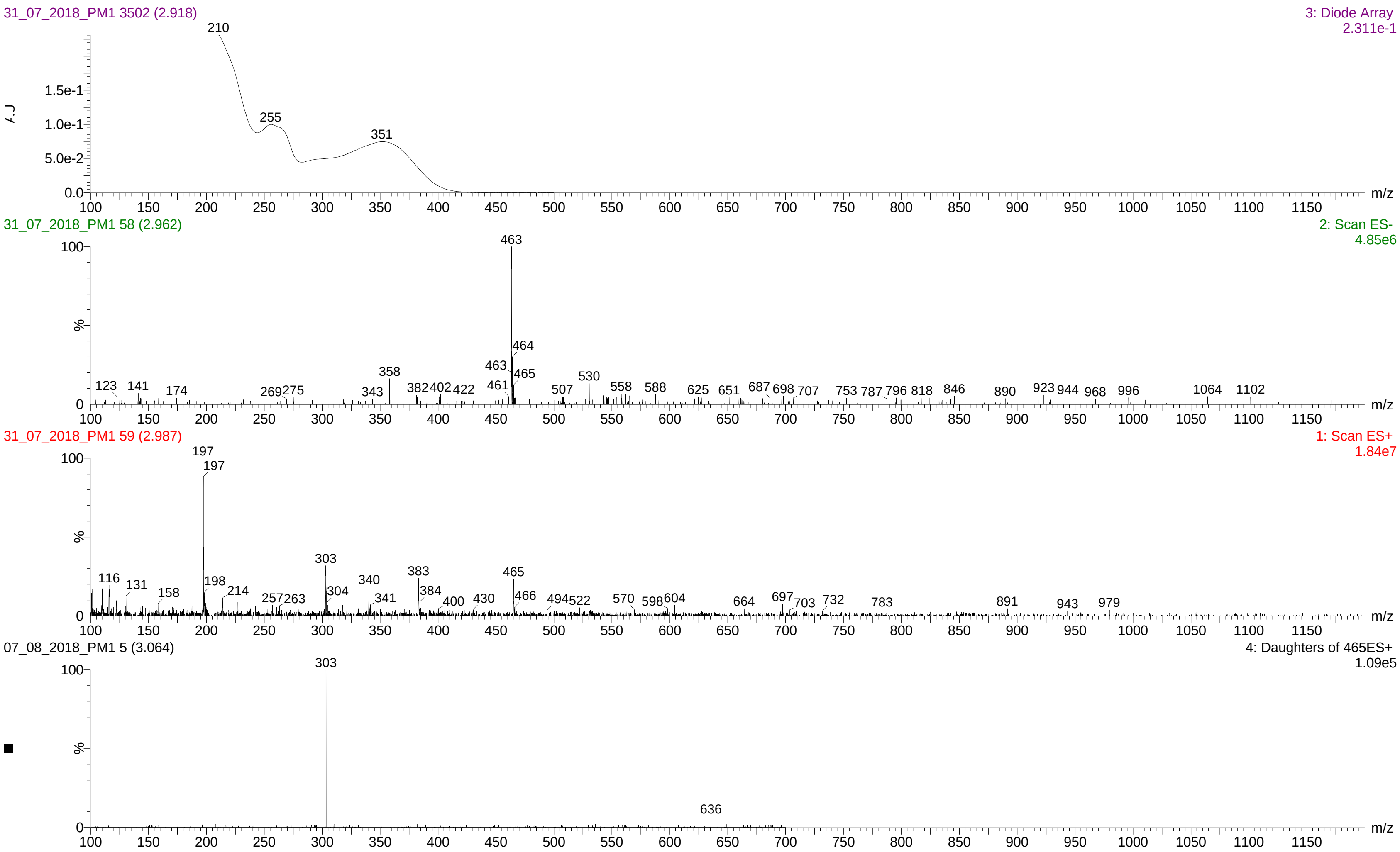

Figure S5. DAD, ESI-MS in the negative and positive ionization modes, and ESI-MS/MS spectra obtained online by UPLC-DAD-ESI-MS/MS for chromatographic peak 2.

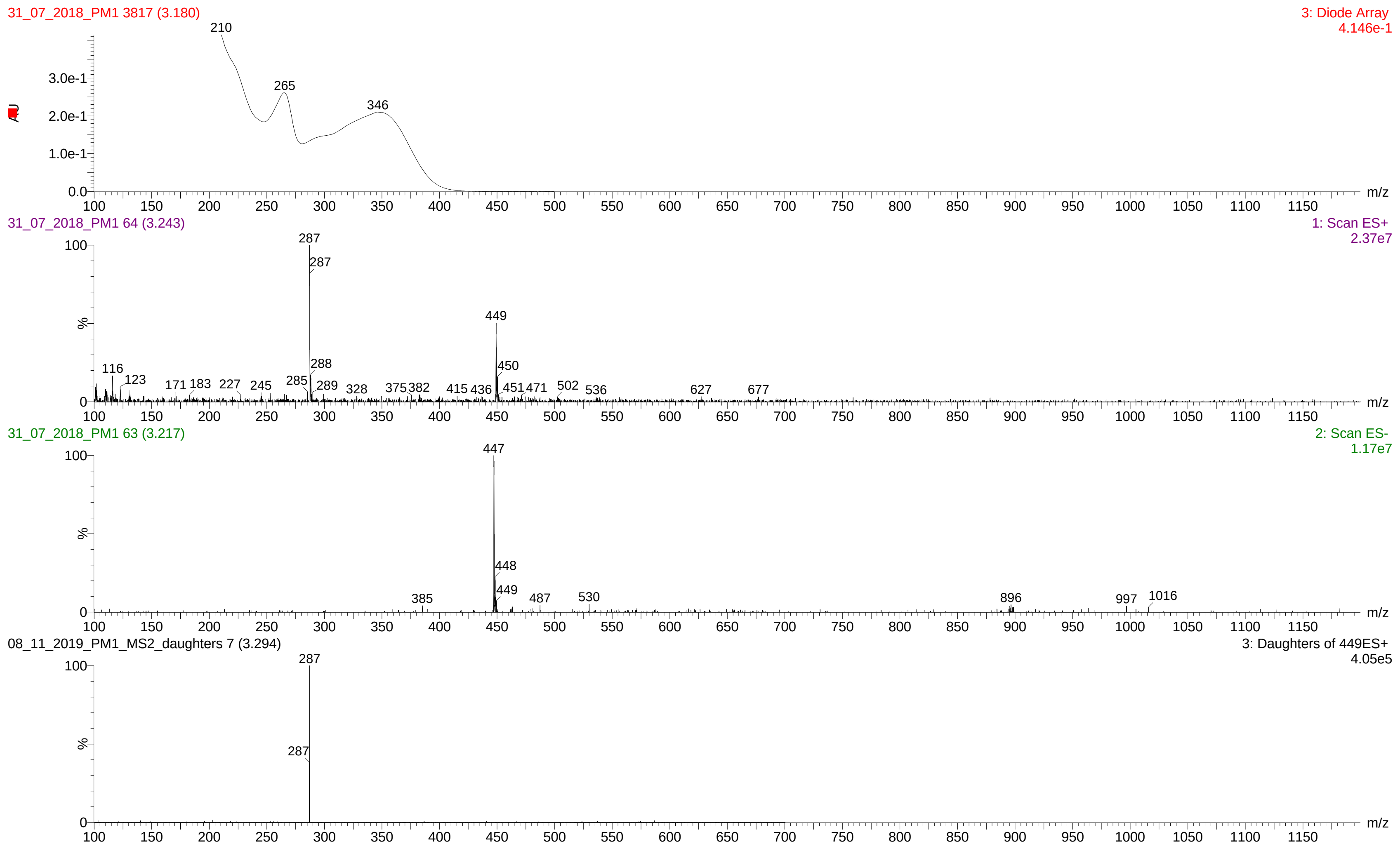

Figure S6. DAD, ESI-MS in the negative and positive ionization modes and ESI-MS/MS spectra obtained online by UPLC-DAD-ESI-MS/MS for chromatographic peak 3.

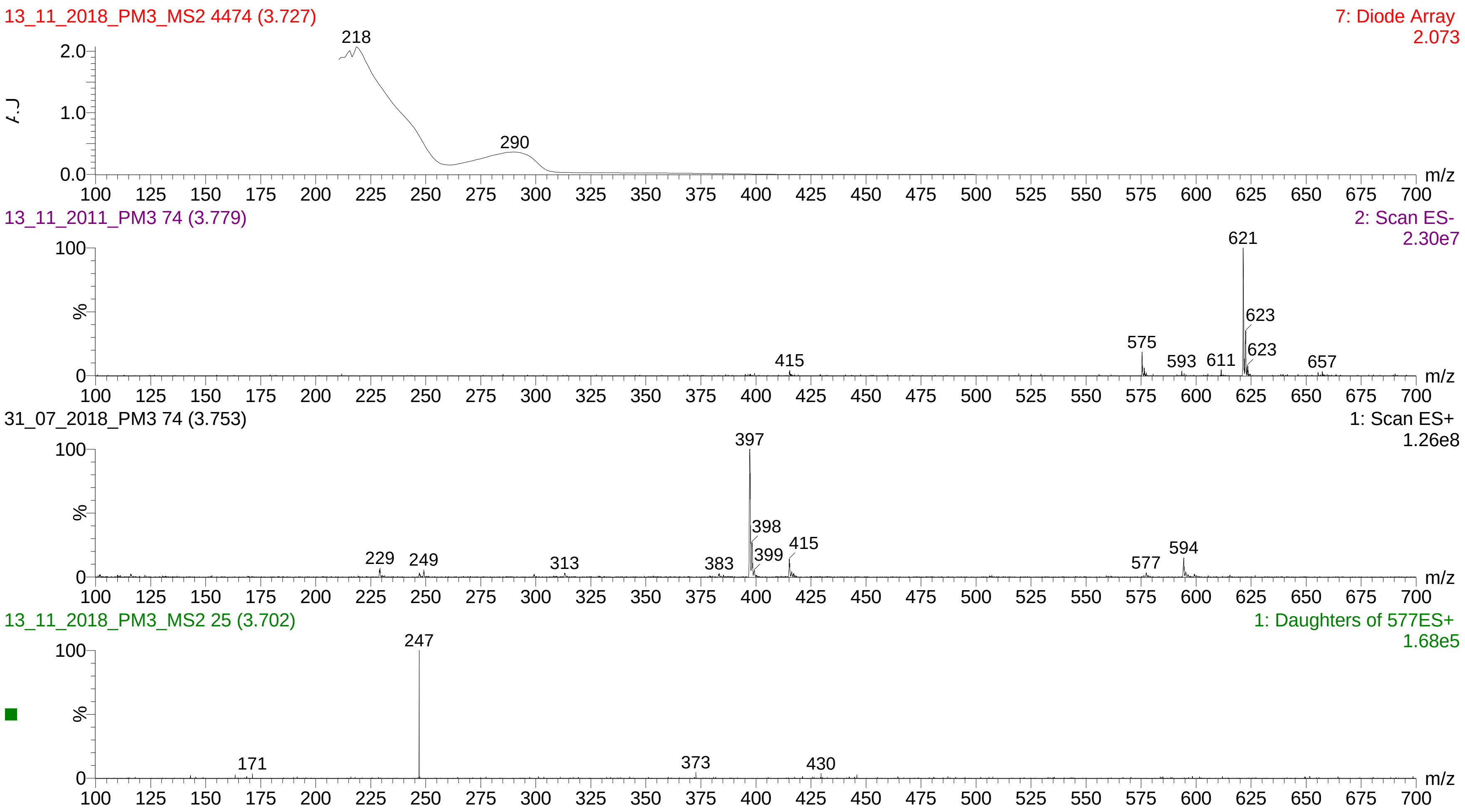

Figure S7. DAD, ESI-MS in the negative and positive ionization modes, and ESI-MS/MS spectra obtained online by UPLC-DAD-ESI-MS/MS for chromatographic peak 4.

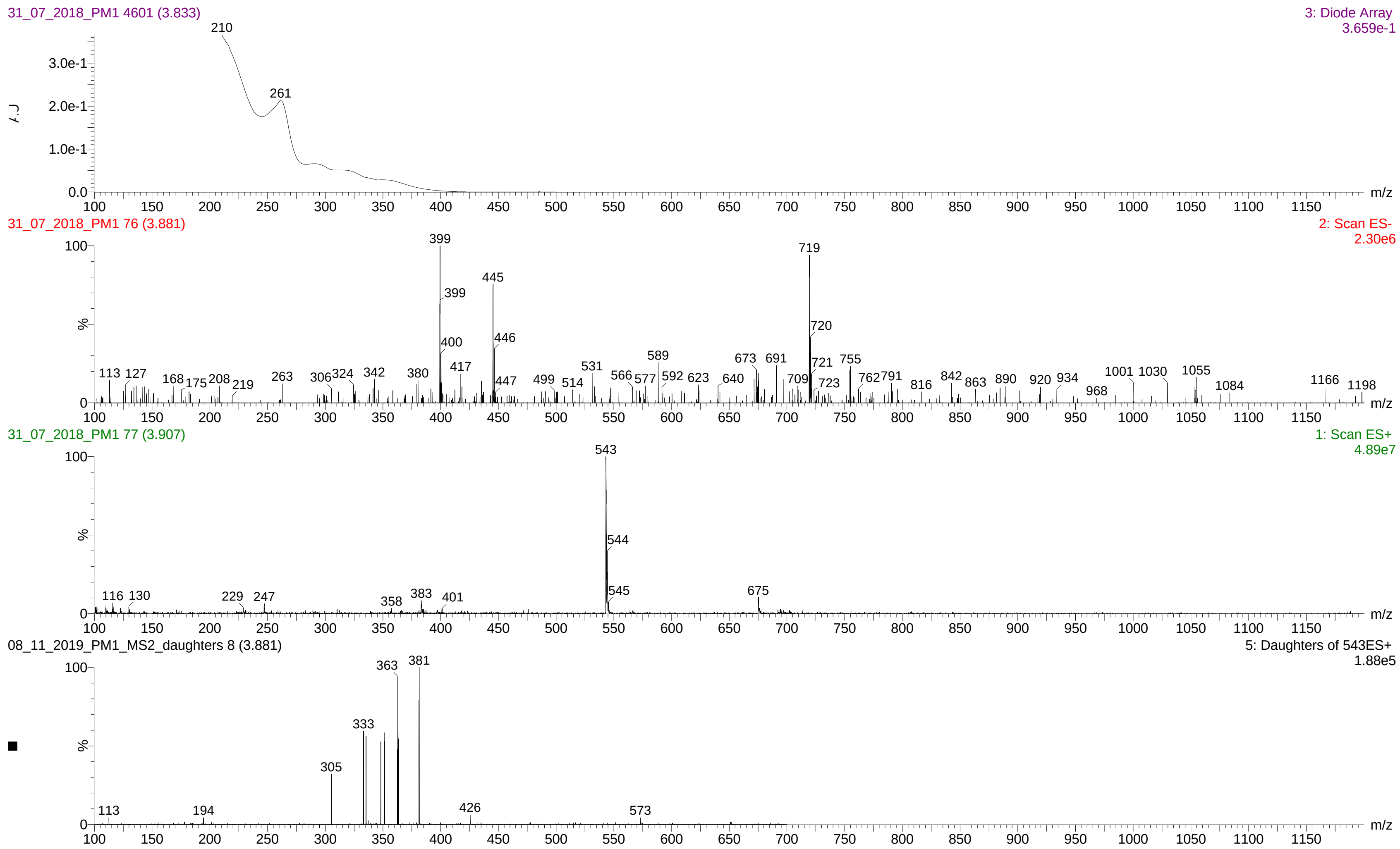

Figure S8. DAD, ESI-MS in the negative and positive ionization modes, and ESI-MS/MS spectra obtained online by UPLC-DAD-ESI-MS/MS for chromatographic peak 5.

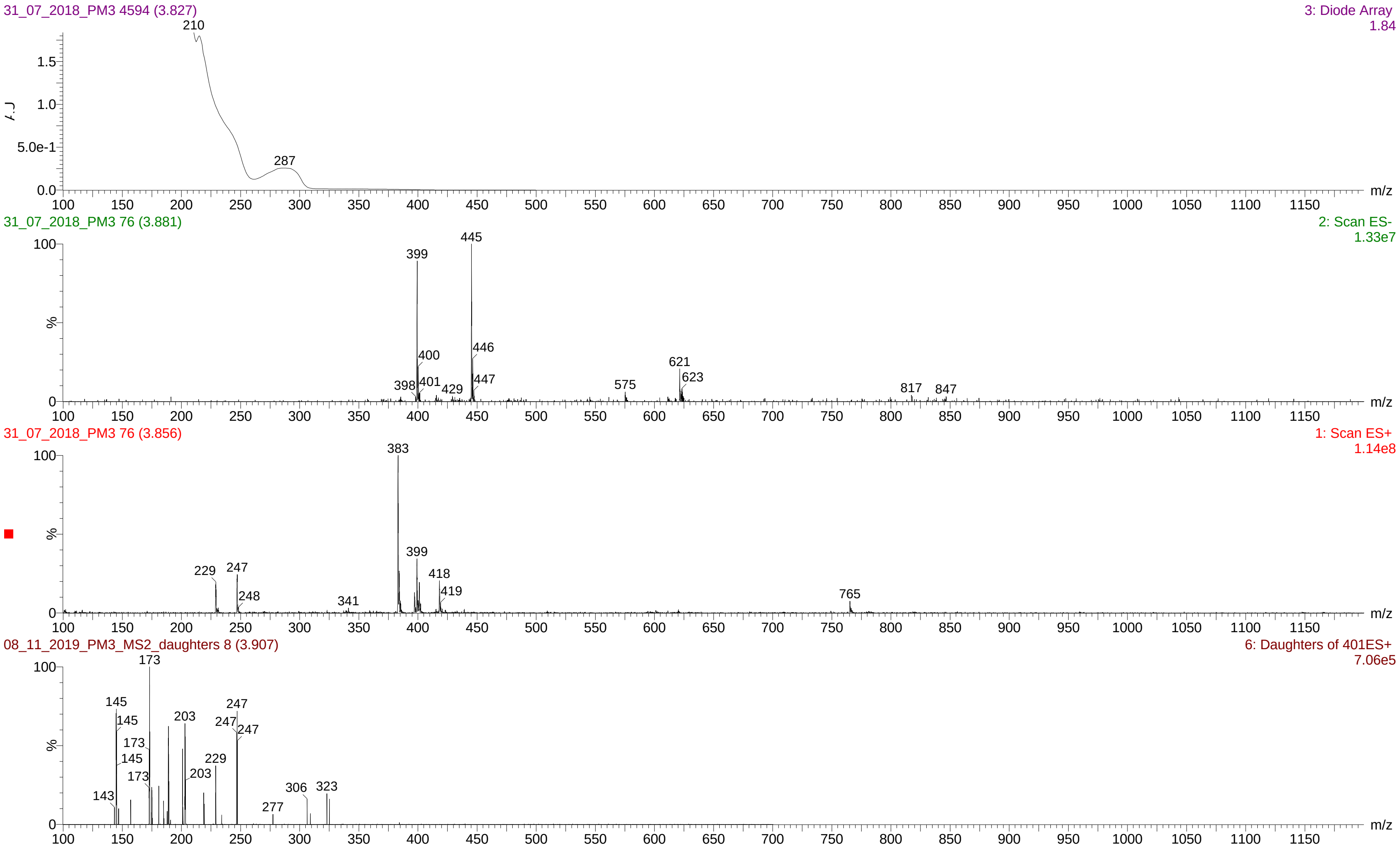

Figure S9. DAD, ESI-MS in the negative and positive ionization modes, and ESI-MS/MS spectra obtained online by UPLC-DAD-ESI-MS/MS for chromatographic peak 5'.

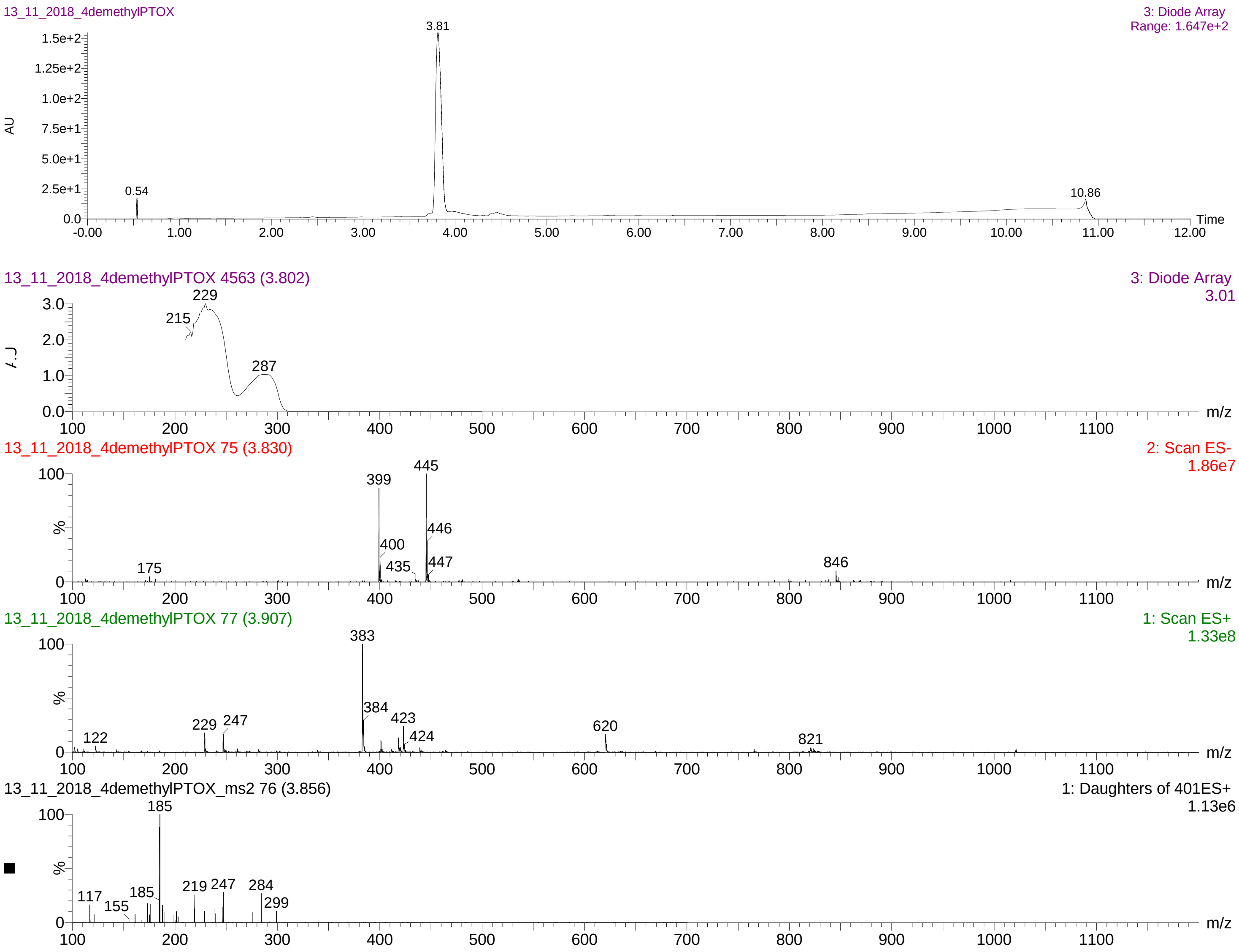

S10. DAD, ESI-MS in the negative and positive ionization modes, and ESI-MS/MS spectra obtained online by UPLC-DAD-ESI-MS/MS for 4’-demethylpodophyllotoxin.

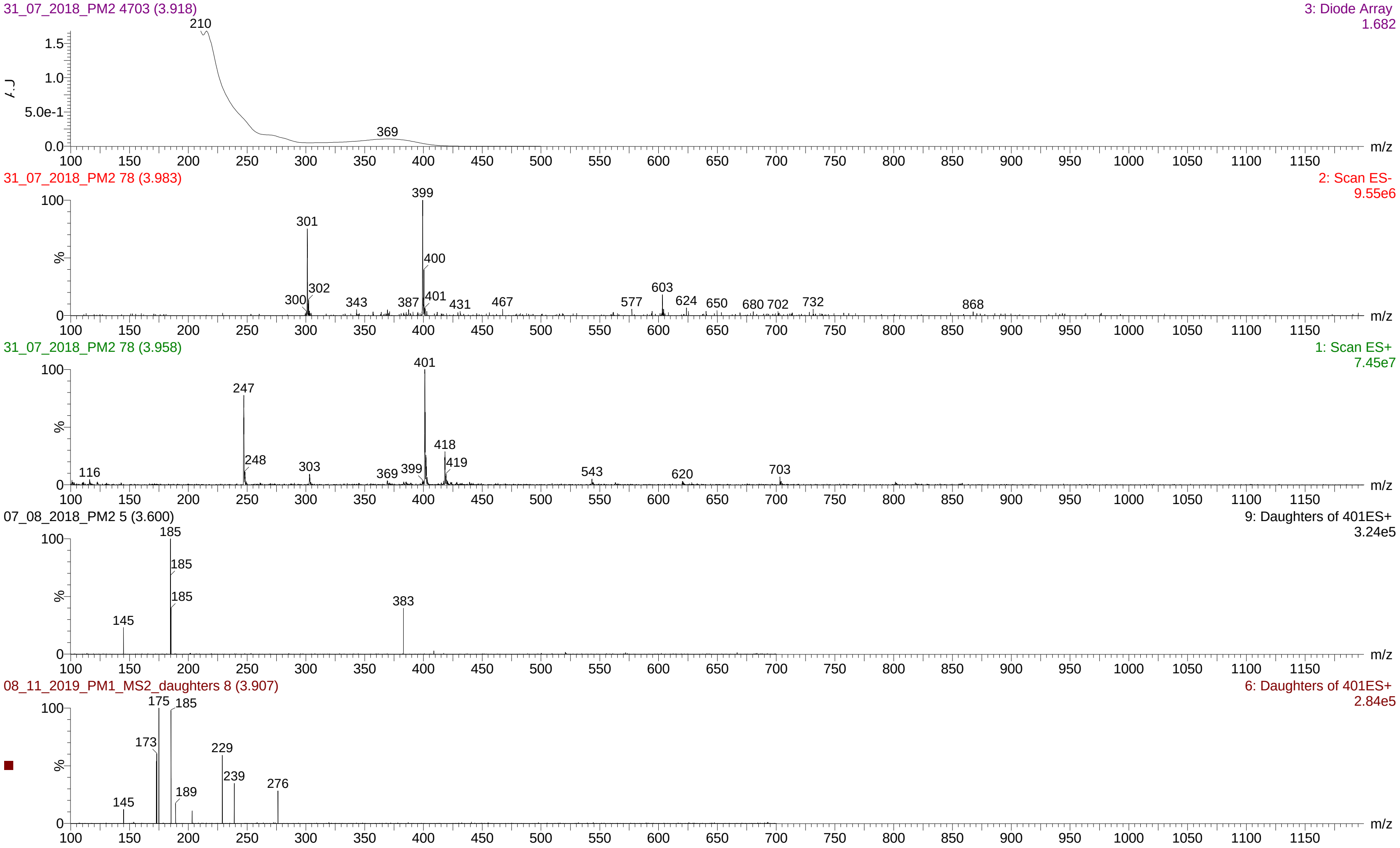

Figure S11. DAD, ESI-MS in the negative and positive ionization modes, and ESI-MS/MS spectra obtained online by UPLC-DAD-ESI-MS/MS for chromatographic peak 6.

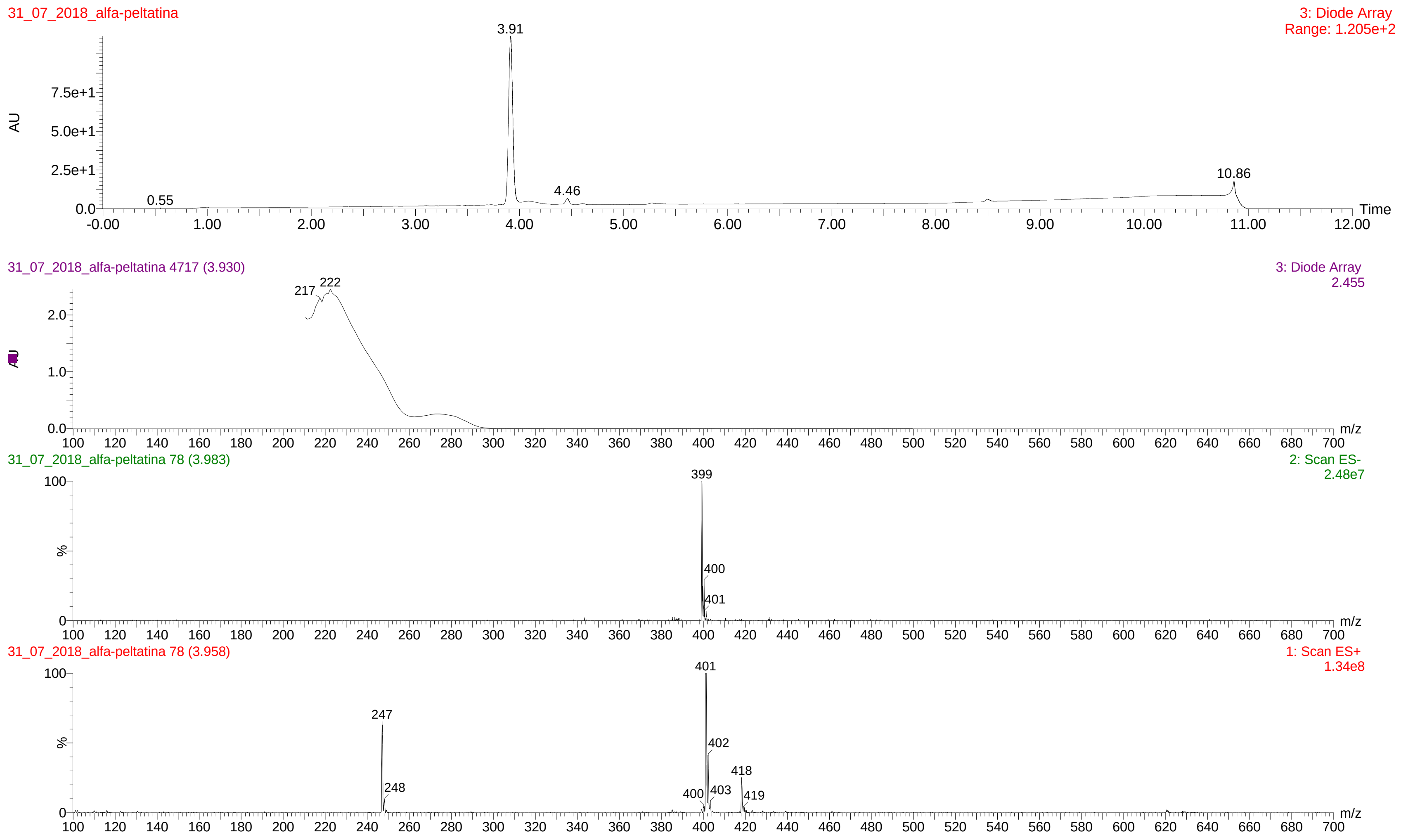

Figure S12. DAD, ESI-MS in the negative and positive ionization modes, and ESI-MS/MS spectra obtained online by UPLC-DAD-ESI-MS/MS for alpha-peltatin.

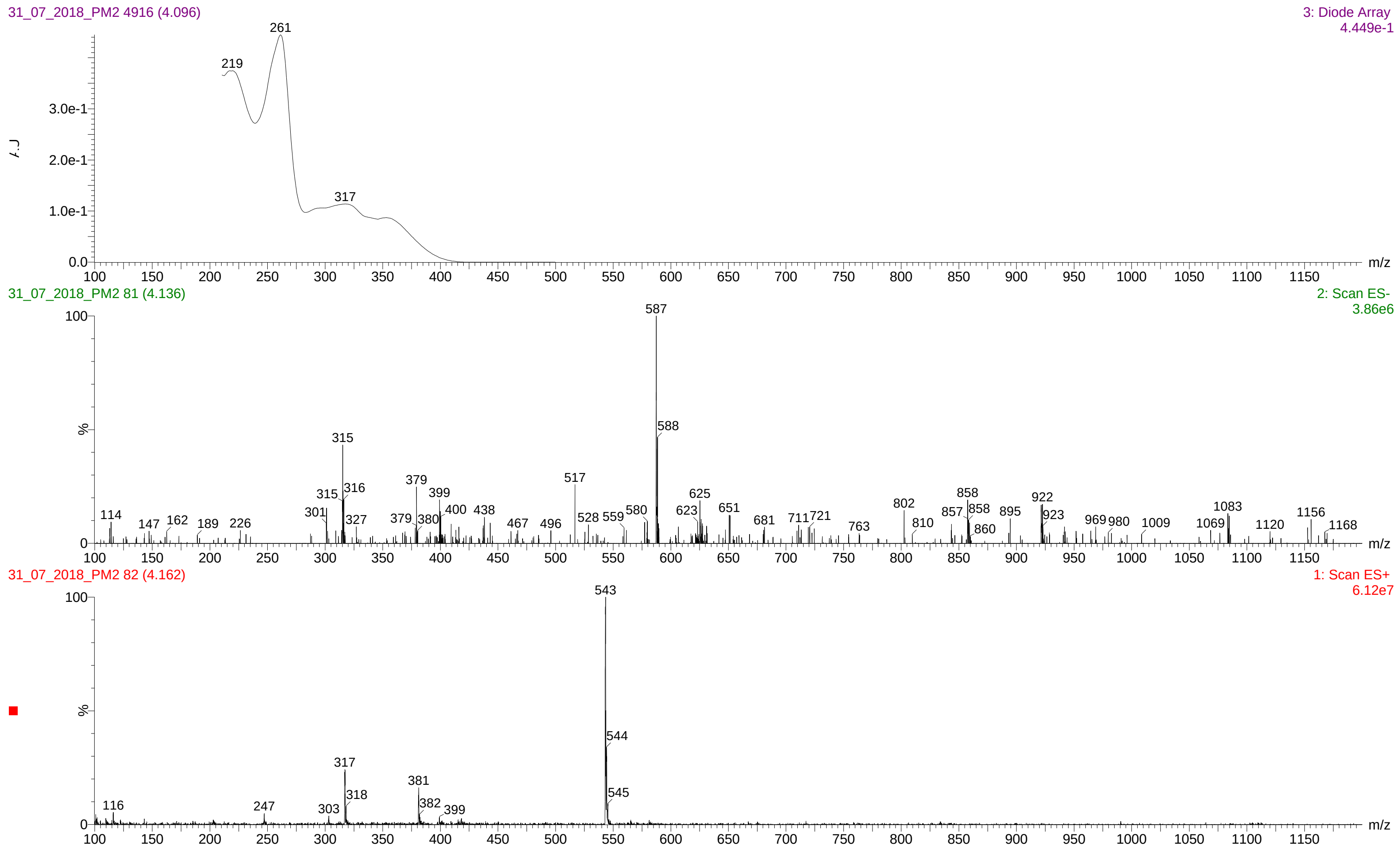

Figure S13. DAD, ESI-MS in the negative and positive ionization modes, and ESI-MS/MS spectra obtained online by UPLC-DAD-ESI-MS/MS for chromatographic peak 7.

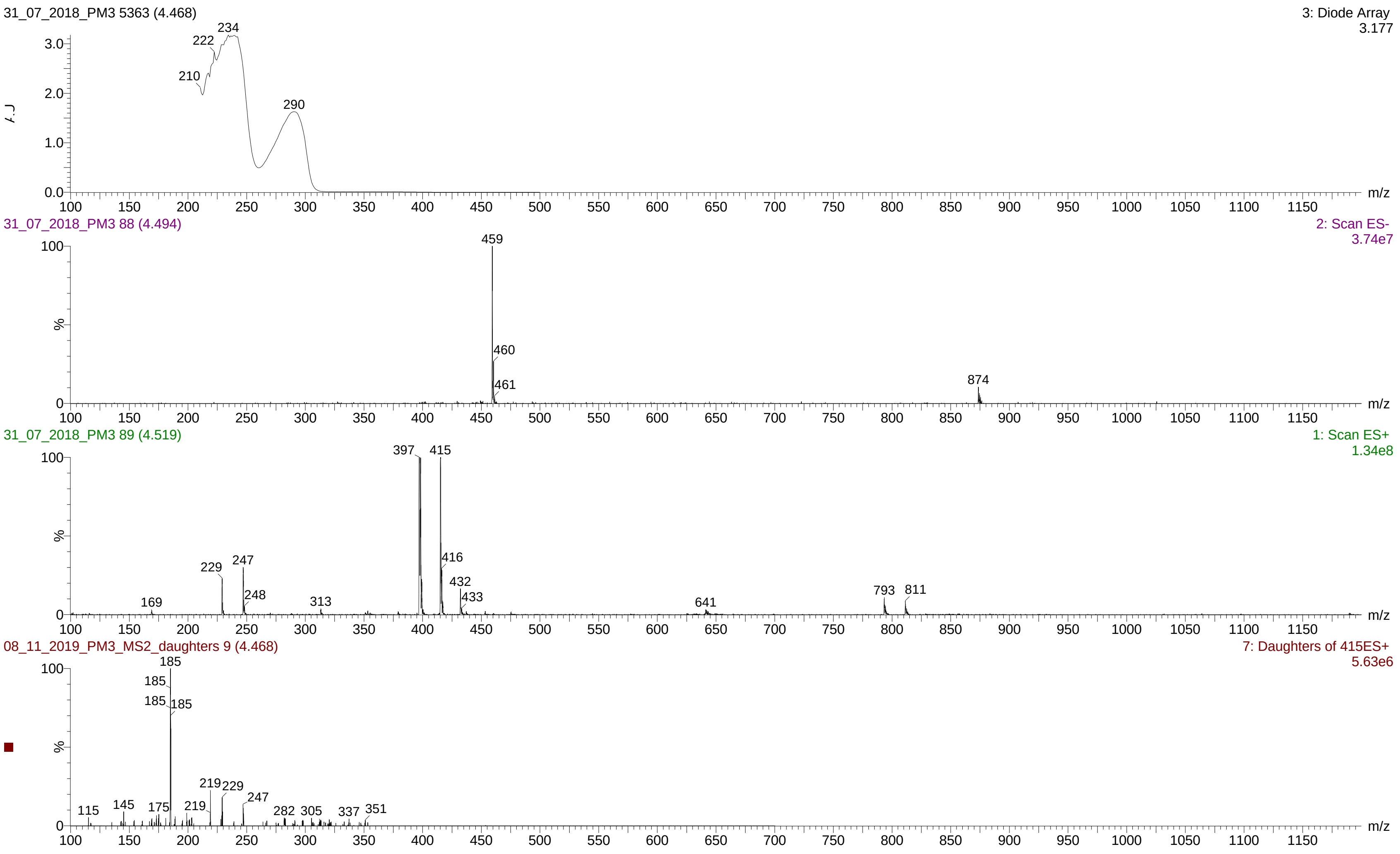

Figure S14. DAD, ESI-MS in the negative and positive ionization modes, and ESI-MS/MS spectra obtained online by UPLC-DAD-ESI-MS/MS for chromatographic peak 8.

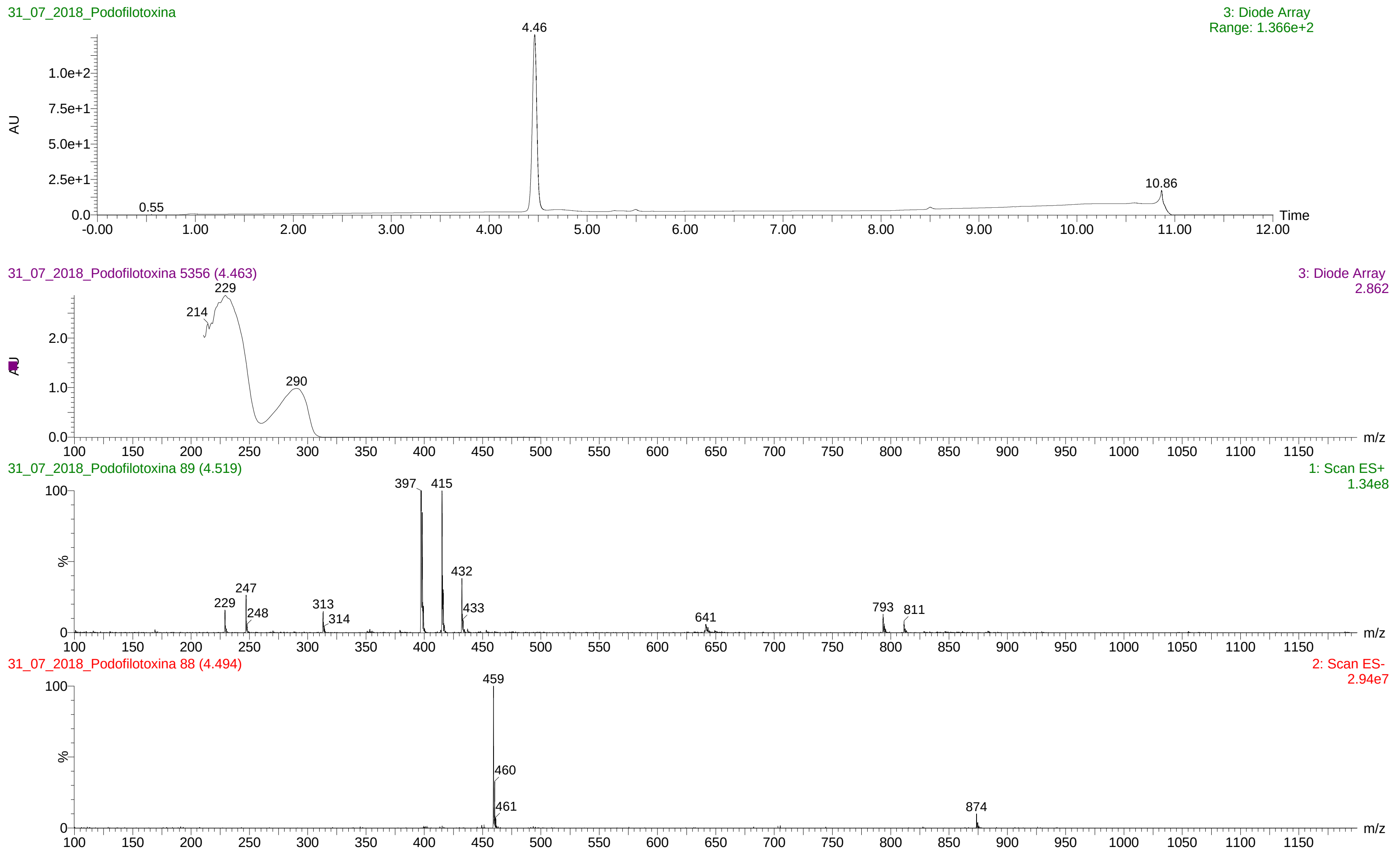

Figure S15. DAD, ESI-MS in the negative and positive ionization modes, and ESI-MS/MS spectra obtained online by UPLC-DAD-ESI-MS/MS for podophyllotoxin.

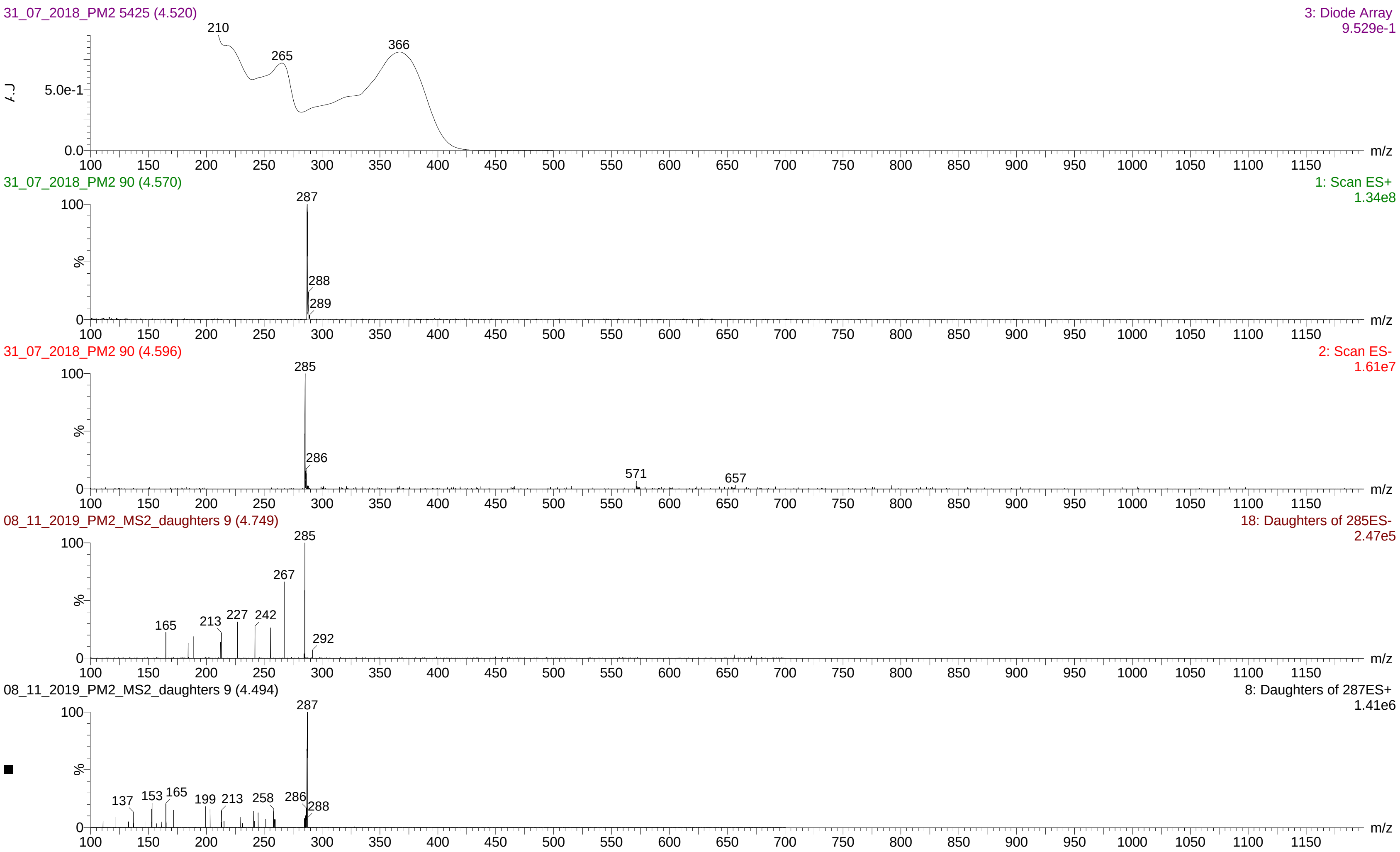

Figure S16. DAD, ESI-MS in the negative and positive ionization modes, and ESI-MS/MS spectra obtained online by UPLC-DAD-ESI-MS/MS for chromatographic peak 9.

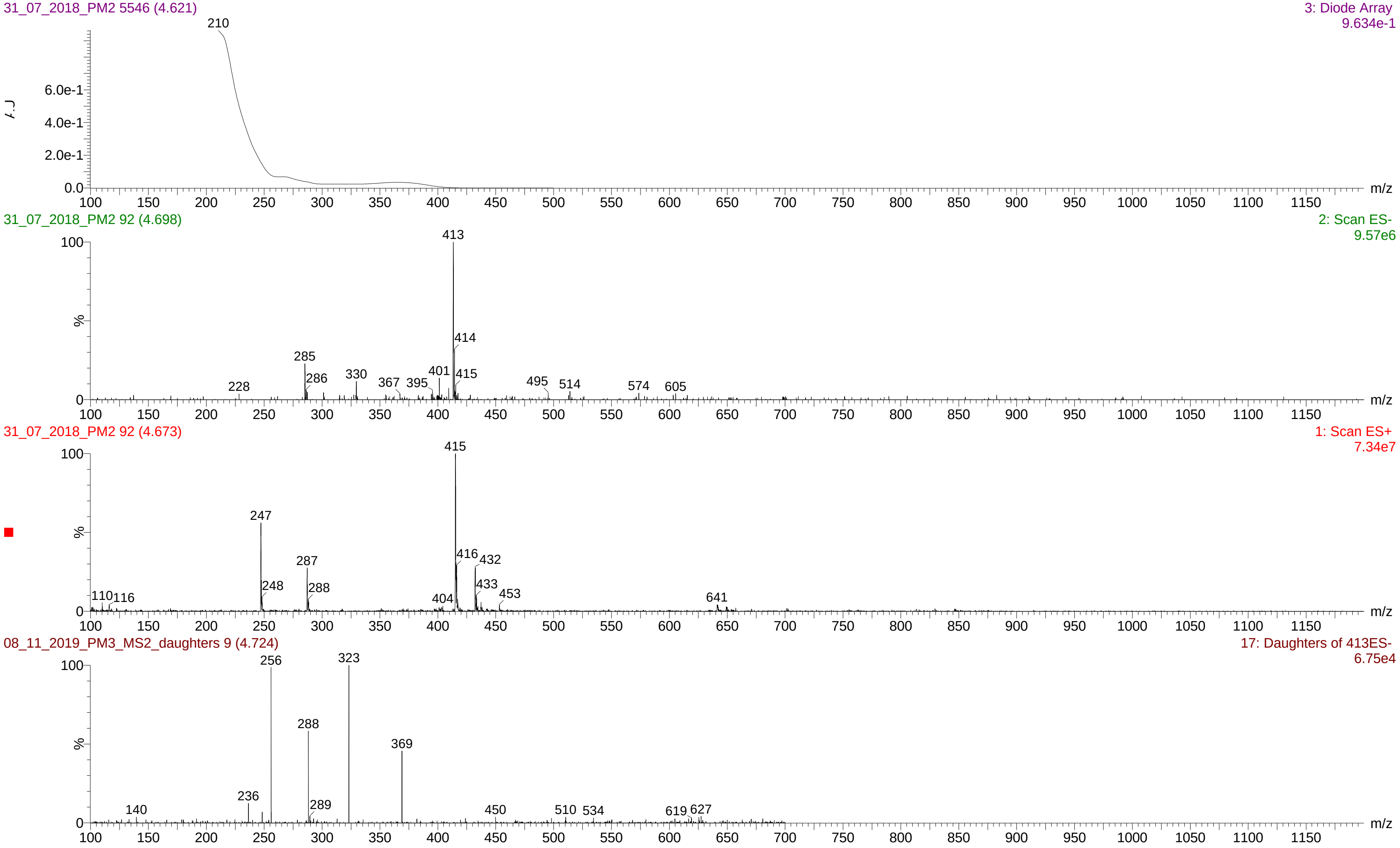

Figure S17. DAD, ESI-MS in the negative and positive ionization modes, and ESI-MS/MS spectra obtained online by UPLC-DAD-ESI-MS/MS for chromatographic peak 10.

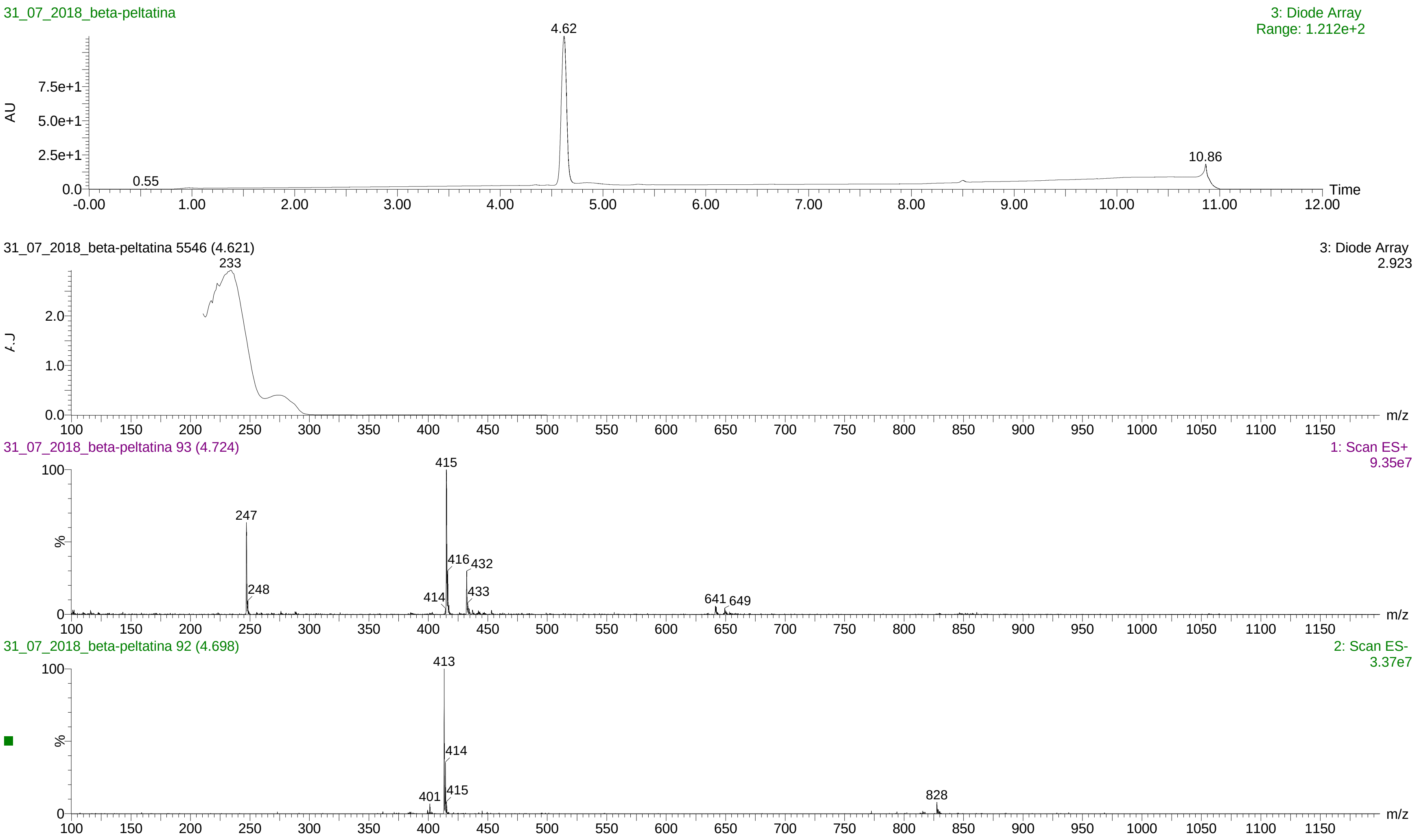

Figure S18. DAD, ESI-MS in the negative and positive ionization modes, and ESI-MS/MS spectra obtained online by UPLC-DAD-ESI-MS/MS for beta-peltatine.

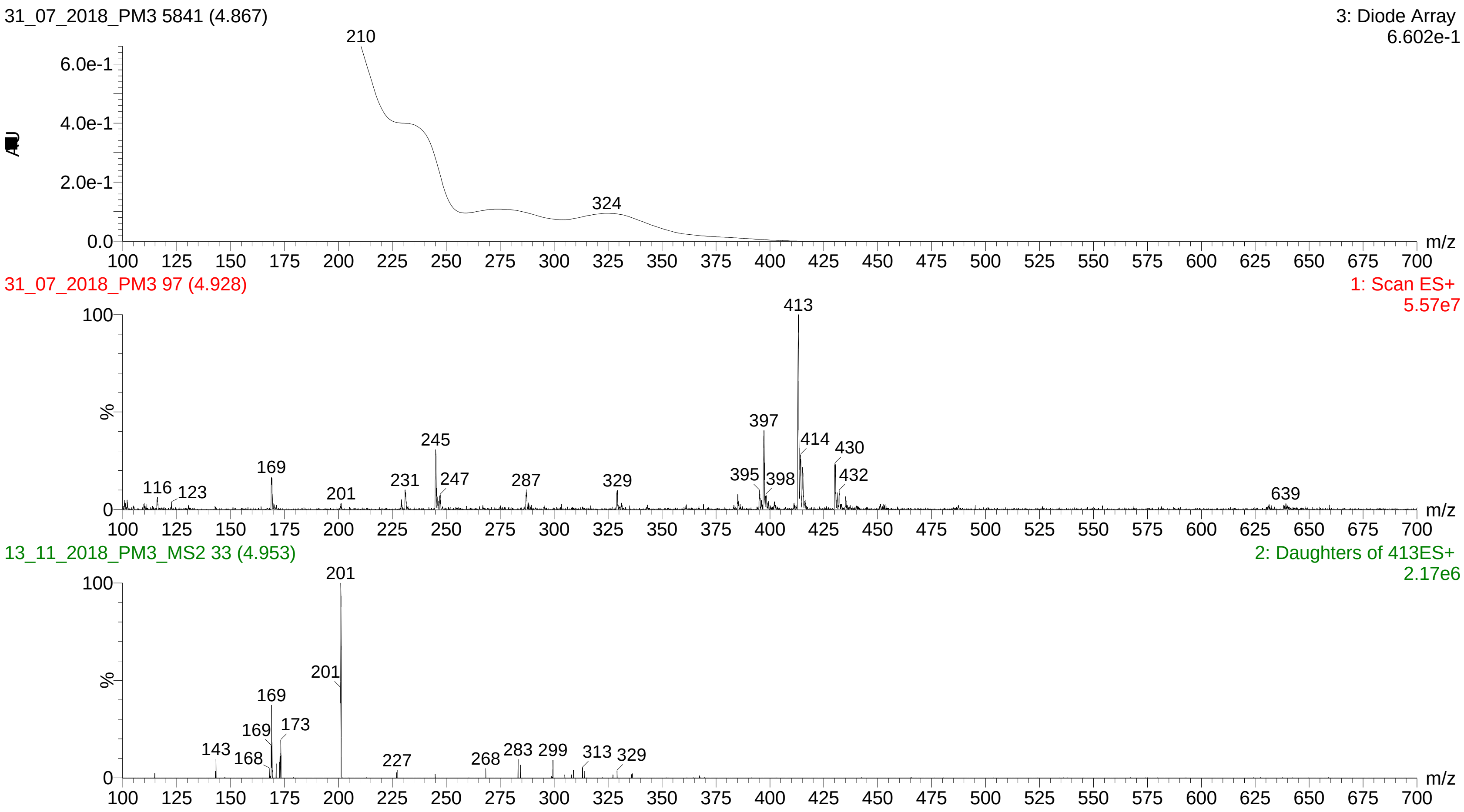

Figure S19. DAD, ESI-MS in positive ionization mode, and ESI-MS/MS spectra obtained online by UPLC-DAD-ESI-MS/MS for chromatographic peak 11.

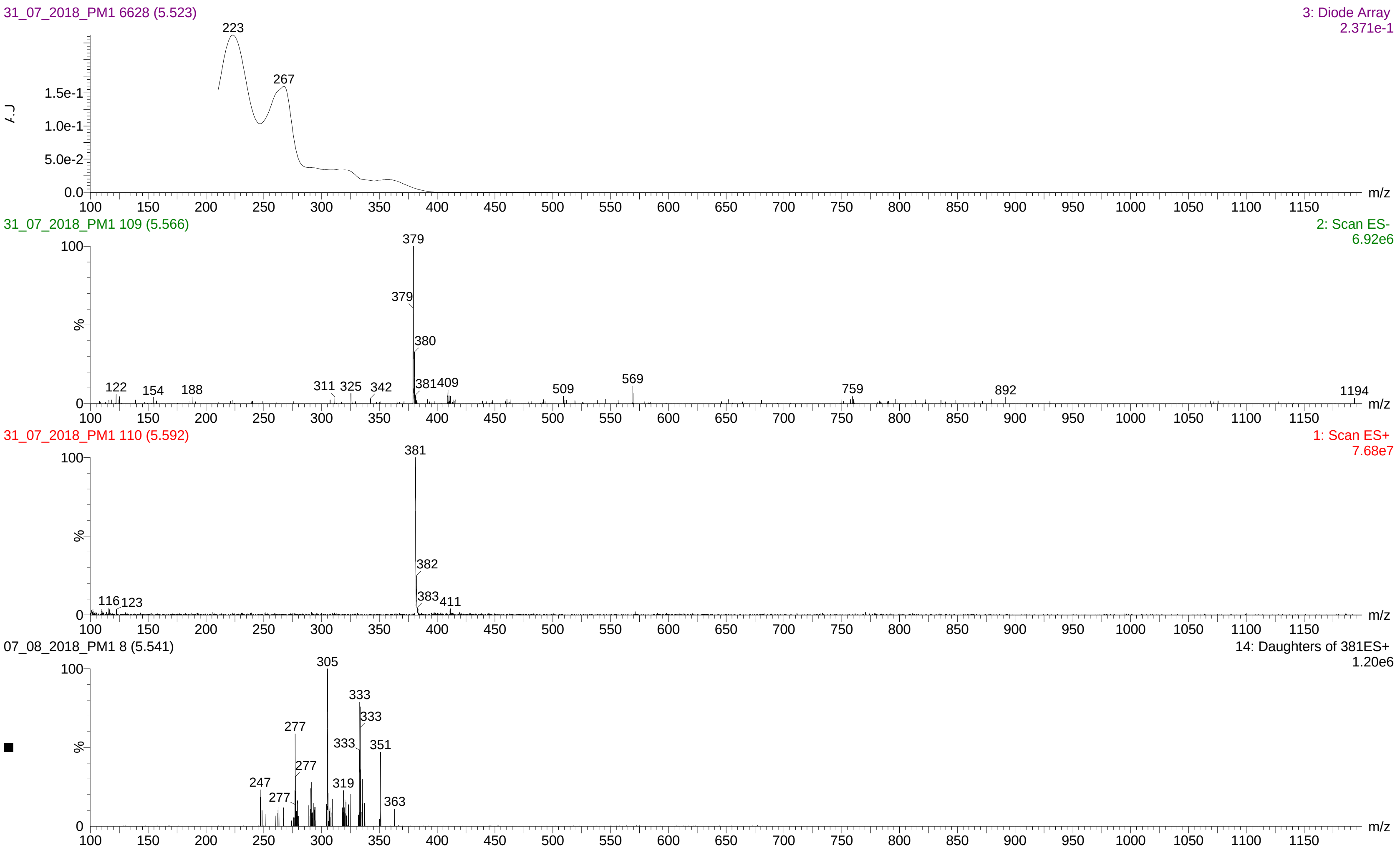

Figure S20. DAD, ESI-MS in the negative and positive ionization modes, and ESI-MS/MS spectra obtained online by UPLC-DAD-ESI-MS/MS for chromatographic peak 12.

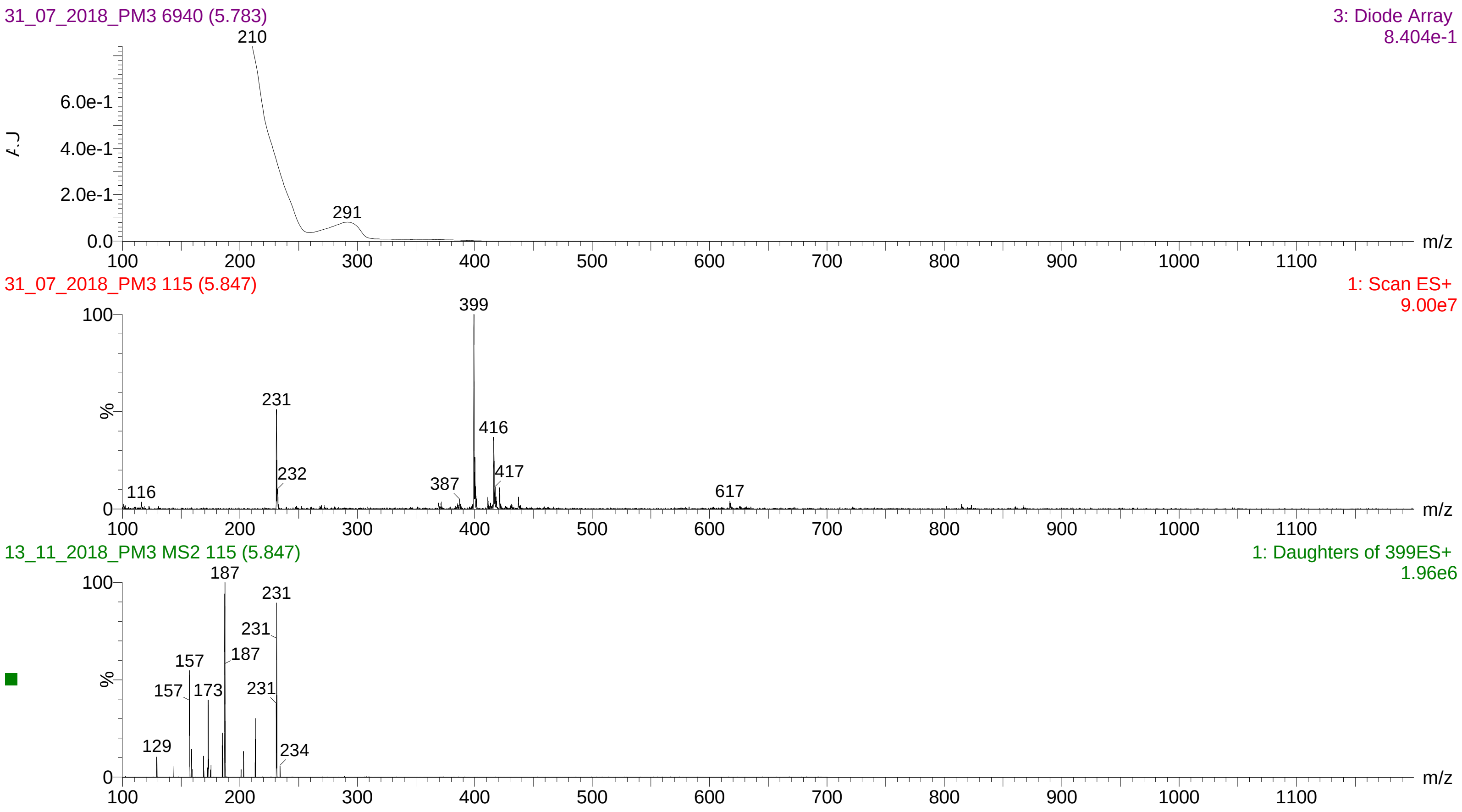

Figure S21. DAD, ESI-MS in the positive ionization mode, and ESI-MS/MS spectra obtained online by UPLC-DAD-ESI-MS/MS for chromatographic peak 13.

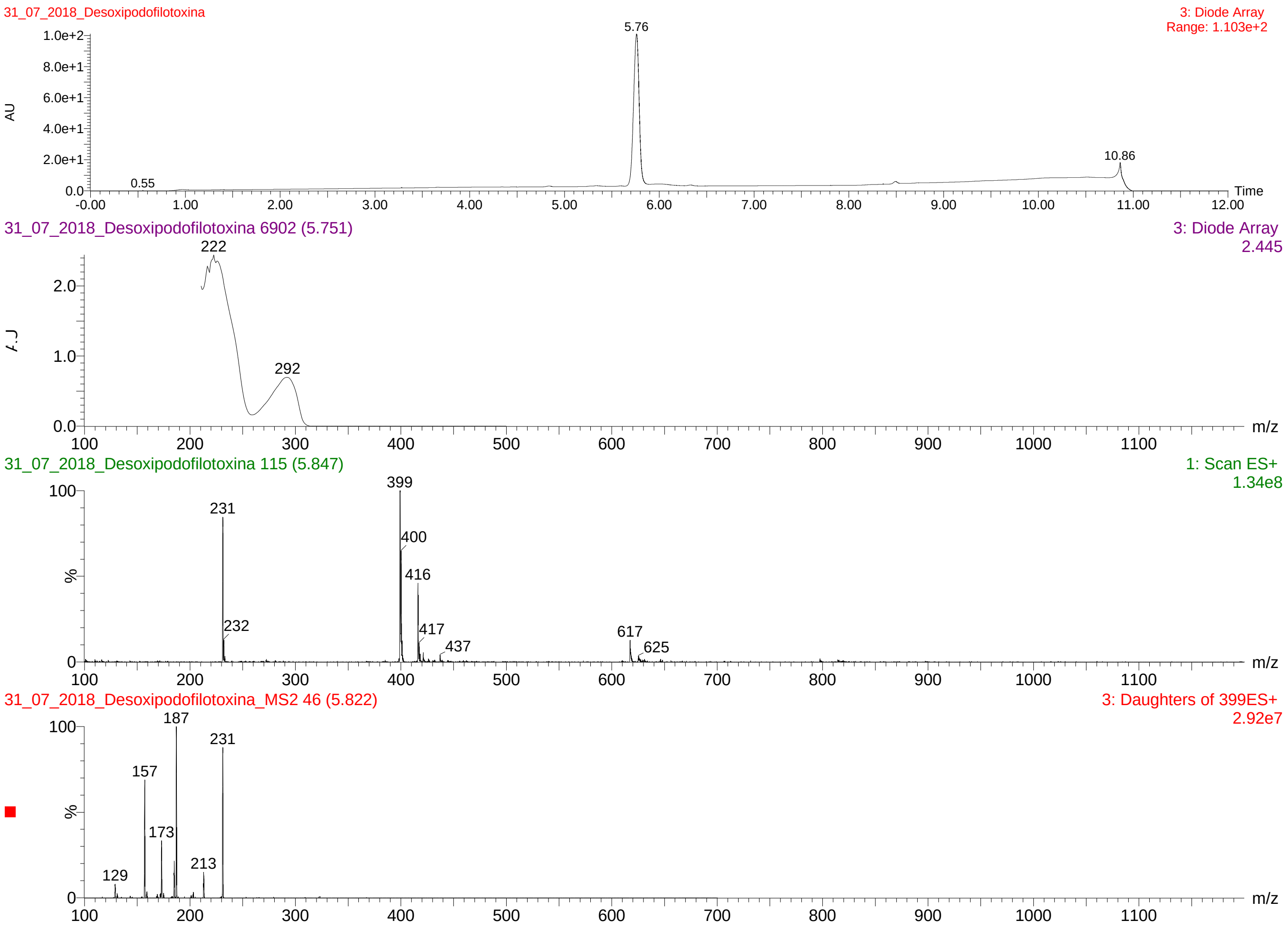

Figure S22. DAD, ESI-MS in the positive ionization mode, and ESI-MS/MS spectra obtained online by UPLC-DAD-ESI-MS/MS for deoxypodophyllotoxin.
