# Supplementary material for: Combined In Vitro Studies and in Silico Target Fishing for the Evaluation of the Biological Activities of Diphylleia cymosa and Podophyllum hexandrum
Source: Molecules. 2018 Dec 13;23(12):3303. doi: 10.3390/molecules23123303 (PMC6321136; doi:10.3390/molecules23123303)
Supplement: Supplementary file 1 [file molecules-23-03303-s001.zip › molecules-403221-SM - original/Tables S1-S2-S3-S4-S5_Biological activities-Methods_V6.pdf]

Table S1: Biological activities better classified by the SVM method for the group of twenty seven lignans analyzed. The column #Pairs represents the number of occurrences of compounds/models with values of **Pa-Pi greater or equal to 0.25**, with Pa-Pi minimum (pessimistic range) greater than zero. The column #model shows the total number of different models associated with activity class. The score was calculated by dividing the #Pairs by 27, and dividing by the root square of the number of models. The Best Model column shows one of the class models. The Activity column is related to the number of active and inactive compounds found in relation to the number of active and inactive compounds belonging to the same activity class.

Entry	Activity Class	#Pairs	#models	Score	Best model	Activity	Description	Targets	TTD-Type	TTD-Disease(2017)
1	Cytotoxicity, Human Lymphoblastoid Cells	482	37	2.93	994		lymphoblastoid cell line			
2	Anti-Inflammatory model	345	33	2.22	435020	act=4/5 inact=1/5	NF-kB activation. Many cellular pathways leading to activation of NF-kB-family transcription factors have been identified to be participating in host-defense, immunity, inflammation, and cancer.			
3	Bacteria, Salmonella Typhimurium	128	8	1.68	2840					
4	Ape1 Endonuclease	77	3	1.65	2572		overexpression of APE1 in many cancers and resistance of these tumor cells to radio- and chemotherapy. Thus, targeting APE1 could improve the efficacy of current treatment paradigms by promoting selective sensitization or protection of diseased and normal cells, respectively.	Chain A, Human Ape1 Endonuclease With Bound Abasic Dna And Mn2+ Ion	Clinical Trial target	Ocular cancer
5	Agonista, p53	75	3	1.6	3	act=2/2 inact=1/2	p53, a tumor suppressor protein, is activated following cellular insult, including DNA damage and other cellular stresses. The activation of p53 regulates cell fate by inducing DNA repair, cell cycle arrest, apoptosis, or cellular senescence. The activation of p53, therefore, is a good indicator of DNA damage and other cellular stresses.	Cellular tumor antigen p53		
6	Genotoxicity, ATAD5	104	6	1.57	720516	act=5/5 inact=1/1	ATPase family AAA domain-containing protein 5. Involved in DNA damage response. Involved in a RAD9A-related damage checkpoint, a pathway that is important in determining whether DNA damage is compatible with cell survival or whether it requires cell elimination by apoptosis. Modulates the RAD9A interaction with BCL2 and thereby induces DNA damages-induced apoptosis.	ATPase family AAA domain-containing protein 5		
7	GLI family zinc finger 1	83	4	1.54	651994		Glioma, B-cell lymphoma, sarcoma. Medulloblastoma, the most common pediatric brain tumor. Hh pathway dysregulation has	Gli1	Research target	

							been linked to tumors of the brain, skin, pancreas, breast, ovaries, and blood, and small molecules targeting this pathway are now being pursued as anti-cancer therapies.			
8	Plasmodium falciparum	295	54	1.49	1886	act=2/4 inact=0/3	Malaria			
9	Anticancer, RecQ-Like Dna Helicase 1 (RECQ1)	69	3	1.48	2708		Human RECQ1 Is a DNA Damage Responsive Protein Required for Genotoxic Stress Resistance and Suppression of Sister Chromatid Exchanges. Developing drugs that interfere with DNA repair, which could sensitize cancer cells to conventional therapy.	Chain A, Structure Of Human Recq-like Helicase In Complex With A Dna Substrate		
10	Mycobacterium tuberculosis	212	29	1.46	504703					
11	Microphthalmia-associated transcription factor	107	8	1.4	493177		MITF-related melanoma and renal cell carcinoma predisposition syndrome. nervous system disease, eye disease, genetic disorder, skin disease, metabolic disease	Microphthalmia-associated transcription factor		
12	Angiogenesis model	74	4	1.37	1117340	act=2/2 inact=1/1	Co-culture of endothelial colony forming cells (ECFC) with adipose-derived stromal cells (ADSC)			
13	Cytotoxicity, isogenic chicken DT40 cell line	64	3	1.37	743012	act=6/6 inact=0/0	Identification of genotoxic compounds			
14	Cell Cycle Modulation (counterscreen)	51	2	1.34	1117349	act=2/2 inact=0/0				
15	serine/threonine-protein kinase 33	60	3	1.28	588632		The serine/threonine kinase 33, STK33, has been identified and shown to be required for the survival and proliferation of mutant KRAS-dependent cells involved in cancer	serine/threonine-protein kinase 33 isoform a	Research target	
16	muscleblind-like protein 1	49	2	1.28	493205		Dystrophia myotonica 1 (DM1). A muscular disorder characterized by myotonia, muscle wasting in the distal extremities, cataract, hypogonadism, defective endocrine functions, male baldness and cardiac arrhythmias.	muscleblind-like protein 1 isoform a		
17	Herpes simplex virus Virion Protein 16 (counterscreen)	102	9	1.26	687007	act=1/1 inact=0/4	Virus, Herpes	transactivating tegument protein VP16		
18	ADMET, Cytochrome P450 3A4	68	4	1.26	625251	act=1/1 inact=0/1	Cytochromes P450 are a group of heme-thiolate monooxygenases. In liver microsomes, this enzyme is involved in an NADPH-dependent electron transport pathway. It performs a variety of oxidation reactions (e.g. caffeine 8-oxidation, omeprazole sulphoxidation, midazolam 1'-hydroxylation and midazolam 4-hydroxylation) of structurally unrelated compounds, including steroids, fatty acids, and xenobiotics. Acts as a 1,8-cineole 2- exo-monooxygenase. The enzyme also hydroxylates etoposide.	Cytochrome P450 3A4	Clinical Trial target	Anxiety disorder; Cancer

19	Osteoporosis model	59	3	1.26	1117326	act=3/3 inact=0/0	Wnt potentiation module is to identify agents that may serve as novel medications for bone development and, specifically, osteoporosis			
20	regulator of G-protein signaling 8	48	2	1.26	1869		Since GPCRs control numerous physiologic processes in diverse tissues, including brain, heart, liver, and lung, modulation of the RGS/G protein interaction has become an attractive target for drug discovery.	RGS8 protein		
21	Virus, HIV-1	150	20	1.24	652241			Vif		
22	Agonista, estrogen receptor alpha (ER-alpha)	67	4	1.24	743077		Estrogen receptor alpha. Estrogen insensitivity syndrome is a very rare condition characterized by a defective ERα that is insensitive to estrogens. The clinical presentation of a female was observed to include absence of breast development and other female secondary sexual characteristics at puberty, hypoplastic uterus, primary amenorrhea, enlarged multicystic ovaries and associated lower abdominal pain, mild hyperandrogenism (manifested as cystic acne), and delayed bone maturation as well as an increased rate of bone turnover.	estrogen nuclear receptor alpha	Successful target	Acne vulgaris; Adrenocortical carcinoma; Advanced prostate cancer; Arthralgia; Atrophic vaginitis; Atrophy; Autoimmune diabetes; Brain cancer; Breast cancer; Prostate cancer; Female infertility; Cancer; Carcinoma; Contraception; Dysmenorrhea; Dyspareunia; Estrogen deficiency; Eye disorders; Female hypogonadism; Female infertility; Female infertility due to anovulation; Female sexual dysfunction; Gonorrheal vaginitis; Hormone deficiency; Hormone replacement therapy; Hyperlipidaemia; Hypogonadism; Infertility; Irregularities; Leukemia; Menopausal disorder;

										Postmenopausal disorder; Menopause symptoms; Hormone refractory prostate cancer; Menorrhagia; Multiple scierosis; Neurological disease; Oral contraceptives; Osteopetrosis; Osteoporosis; Pain; Post-menopausal vaginal atrophy; Trematode infection; Vagina disease
23	peripheral myelin protein 22	47	2	1.23	624044	act=2/2 inact=0/0	Destabilization of myelin and neuropathic disorders, such as Charcot–Marie–Tooth type 1A (CMT1A), Dejerine–Sottas disease, and Hereditary Neuropathy with Liability to Pressure Palsy (HNPP)	peripheral myelin protein 22		
24	Antagonista, estrogen receptor alpha (ER-alpha)	82	7	1.15	743091	act=2/2 inact=0/6	Estrogen receptor alpha. Estrogen insensitivity syndrome is a very rare condition characterized by a defective ERα that is insensitive to estrogens. The clinical presentation of a female was observed to include absence of breast development and other female secondary sexual characteristics at puberty, hypoplastic uterus, primary amenorrhea, enlarged multicystic ovaries and associated lower abdominal pain, mild hyperandrogenism (manifested as cystic acne), and delayed bone maturation as well as an increased rate of bone turnover.	estrogen nuclear receptor alpha	Successful target	Acne vulgaris; Adrenocortical carcinoma; Advanced prostate cancer; Arthralgia; Atrophic vaginitis; Atrophy; Autoimmune diabetes; Brain cancer; Breast cancer; Prostate cancer; Female infertility; Cancer; Carcinoma; Contraception; Dysmenorrhea; Dyspareunia; Estrogen deficiency; Eye disorders; Female hypogonadism; Female infertility; Female infertility due to anovulation; Female sexual dysfunction;

										Gonorrheal vaginitis; Hormone deficiency; Hormone replacement therapy; Hyperlipidaemia; Hypogonadism; Infertility; Irregularities; Leukemia; Menopausal disorder; Postmenopausal disorder; Menopause symptoms; Hormone refractory prostate cancer; Menorrhagia; Multiple scierosis; Neurological disease; Oral contraceptives; Osteopetrosis; Osteoporosis; Pain; Post-menopausal vaginal atrophy; Trematode infection; Vagina disease
25	1-acylglycerol-3-phosphate O-acyltransferase ABHD5	61	4	1.13	651677		lipid disorders such as obesity, diabetes, and cardiovascular disease	1-acylglycerol-3-phosphate O-acyltransferase ABHD5; perilipin-5		
26	ADP Fluorescence Polarization Displacement Assay (counterscreen)	43	2	1.13	2712		0			
27	Giardia lamblia	72	6	1.09	2787			Chain A, Structure Of Giardia Fructose-1,6-biphosphate Aldolase In Complex With Phospho-glycolohydroxamate		
28	Anticancer, Leukemia	109	14	1.08	328	act=11/11 inact=0/5	0			

29	TOR pathway GFP-fusion proteins [Saccharomyces cerevisiae]	97	11	1.08	488808		TORC1 is a central hub of a signaling network that couples cues from hormones and growth factors (in mammalian cells), energy and stresses, and the abundance of nutrients, to cell growth and proliferation. Very recent work has elucidated many details of the signaling events upstream of TORC1 as well as downstream targets of TORC1. Importantly, in this context, most negative regulators of mammalian TORC1 (mTORC1)have been previously identified as tumor suppressor gene products, while many positive regulators of mTORC1 have been identified as proto-oncoproteins and/or are found at elevated levels in tumor-derived cell lines.	RPL19A		
30	Antagonista, androgen receptor (AR)	77	7	1.08	743035	act=4/4 inact=0/4	Androgen receptor. The normal development and maintenance of the prostate is dependent on androgen acting through the androgen receptor (AR). AR remains important in the development and progression of prostate cancer. AR ligands are widely used in a variety of clinical applications (i.e., agonists are employed for hypogonadism, while antagonists are used for prostate cancer therapy).	AR protein	Successful target	Acne vulgaris; Atherosclerosis; Advanced prostate cancer; Alcoholic hepatitis; Alopecia; Bladder cancer; Cachexia; Cancer; Cardiovascular disorder; Castration-resistant prostate cancer; Contraception; Cystic fibrosis; Dermatological disease; Dysmenorrhea; Endocrine disease; Female androgenresponsive recurrent mammary cancer; Heart failure; Hormone deficiency; Hormone refractory prostate cancer; Hypogonadism; Male hormonal deficiencies; Male hypogonadism; Breast cancer; Metastatic castration-resistant prostate cancer; Metastatic prostate

										cancer; Muscle atrophy; Osteoporosis; Osteoporosis in post-menopausal women; Pain; Prostate cancer; Prostate hyperplasia; Testosterone deficiency
31	dual specificity protein kinase CLK4	50	3	1.07	1795			dual specificity protein kinase CLK4		
32	Cytotoxicity, Jurkat	63	5	1.04	364	act=1/1 inact=0/1	Organism: Homo sapiens / Tissue: peripheral blood / Cell Type: T Cell/ T Lymphocyte / Disease: acute T cell leukemia. immortalized line of human T lymphocyte cells that are used to study acute T cell leukemia, T cell signaling, and the expression of various chemokine receptors susceptible to viral entry, particularly HIV. Jurkat cells are useful in science because of their ability to produce interleukin 2. Their primary use, however, is to determine the mechanism of differential susceptibility of cancers to drugs and radiation.			
33	regulator of G-protein signaling 7	39	2	1.02	1871		Since GPCRs control numerous physiologic processes in diverse tissues, including brain, heart, liver, and lung, modulation of the RGS/G protein interaction has become an attractive target for drug discovery.	RGS7, partial		
34	Cytotoxicity, L929	39	2	1.02	463117		Organism: Mus musculus, mouse / Tissue: subcutaneous connective tissue; areolar and adipose /			
35	Anticancer, Mixed Lineage Leukemia	39	2	1.02	624162		The MLL (Mixed Lineage Leukemia) gene is involved in chromosomal translocation that results in either acute lymphoid leukemia (ALL) or acute myeloid leukemia (AML). Chromasomal translocation of MLL gene is responsible for the fusion of N-terminal MLL to more than 60 different partner genes in frame. We have miniaturized and optimized an in-vitro assay to screen for inhibitors of the MLL-CXXC domain and HOX-A DNA interaction, which could have utility in MLL fusion leukemias.			
36	Antagonista, retinoid-related orphan receptor gamma (ROR-gamma)	39	2	1.02	1159523	act=2/2 inact=0/0	RAR-related orphan receptor gamma (RORγ). The RORγ protein is a DNA-binding transcription factor and is a member of the NR1	RAR-related orphan receptor gamma		

							subfamily of nuclear receptors. The RORγ isoform appears to be involved in the regulation of circadian rhythms. Also, since the levels of RORγ are rhythmic in some tissues (liver, kidney), it has been proposed to impose a circadian pattern of expression on a number of clock-controlled genes, for example the cell cycle regulator p21. RORγt is the most studied of the two isoforms. Its best understood functionality is in the immune system. The transcription factor is essential for lymphoid organogenesis, in particular lymph nodes and Peyer's patches, but not the spleen. RORγt also plays an important regulatory role in thymopoiesis, by reducing apoptosis of thymocytes and promoting thymocyte differentiation into pro-inflammatory T helper 17 (Th17) cells. It also plays a role in inhibiting apoptosis of undifferentiated T cells and promoting their differentiation into Th17 cells, possibly by down regulating the expression of Fas ligand and IL2, respectively.			
37	Antagonista, thyroid receptor (TR)	38	2	1	743067	act=3/3 inact=0/0	Among the most important functions of thyroid hormone receptors are regulation of metabolism and heart rate. In addition, they play critical roles in the development of organisms. Certain mutations in the thyroid hormone receptor are associated with thyroid hormone resistance.	thyroid hormone receptor beta isoform 2	Successful target	High cholesterol levels in blood; Hyperlipidaemia; Hyperthyroidism; Hypothyroidism; Lipid metabolism disorder; Wound healing
38	yeast SKN7-mediated toxicity (counterscreen)	27	1	1	624258		counterscreen , the aim is to eliminate compounds with non-specific effects on yeast growth	Skn7p		
39	Steroidogenic acute regulatory protein (StAR) promoter (counterscreen)	27	1	1	651611	act=1/1 inact=0/0	Interference with HTS			
40	regulator of G-protein signaling 19	27	1	1	1884		Regulates G protein-coupled receptor signaling cascades. Inhibits signal transduction by increasing the GTPase activity of G protein alpha subunits, thereby driving them into their inactive GDP-bound form (PMID:11602604, PMID:18434541). Plays an important role in the phototransduction cascade by regulating the lifetime and effective concentration of activated transducin alpha. May regulate extra and intracellular mitogenic signals.	regulator of G-protein signaling 19		
41	regulator of G-protein signaling 16	27	1	1	1888		Regulates G protein-coupled receptor signaling cascades. Inhibits signal transduction by	regulator of G-protein signaling 16	Research target	

							increasing the GTPase activity of G protein alpha subunits, thereby driving them into their inactive GDP-bound form. Plays an important role in the phototransduction cascade by regulating the lifetime and effective concentration of activated transducin alpha. May regulate extra and intracellular mitogenic signals.			
42	RanGTP-Importin-beta complex	27	1	1	2823		Ran has been implicated in the regulation of several mitotic events, including spindle assembly, kinetochore assembly, and nuclear envelope assembly. The small molecule inhibitors/probes identified in the screen would be extremely useful for elucidating the mitotic roles of Ran and disrupting the Ran pathway at particular stages in mitosis. This will allow us to assess the importance of this pathway throughout the course of cell division. In addition, the small molecule inhibitors/probes have potential therapeutic value for human disease, such as cancer drug.			
43	Prepl protein	27	1	1	624481		Hypotonia-cystinuria syndrome (HCS) and 2p21 deletion syndrome are debilitating and poorly understood diseases, both involving deletion of the gene encoding prolyl oligopeptidase-like enzyme (PREPL), a member of the prolyl oligopeptidase (POP) family of serine peptidases.	Prepl protein		
44	polyadenylate-binding protein 1	27	1	1	435018		Translation is an essential cellular process whose deregulation is associated with alterations in cell growth, cell cycle progression, and cell death responses. Translation initiation control is usurped upon viral infection and is deregulated in many human cancers. Over-expression of certain translation factors can lead to malignant transformation and many of the components of the translational apparatus are over-expressed in human cancers. These results validate translation initiation as a potential chemotherapeutic target.	polyadenylate-binding protein 1		
45	geminin	27	1	1	463097		geminin are critical regulators of this process and their misregulation results in DNA re-replication in normal and cancer cells.	geminin	Research target	
46	cysteine protease ATG4B	27	1	1	504756		Chemical modulators of autophagy are essentially non-existent, with the only available agent, 3-methyladenine, requiring millimolar concentrations to inhibit class III phosphatidyl inositol kinases (PI3Ks) involved in autophagy.	cysteine protease ATG4B isoform a		

							A need exists for chemicals that target specific components of the autophagy machinery -- both for use as research tools for addressing questions about the role of autophagy in diseases such as cancer.			
47	Cytotoxicity, SK-N-SH	27	1	1	435		Organism: Homo sapiens, human / Tissue: brain; derived from metastatic site: bone marrow / Disease: neuroblastoma.			
48	Cytotoxicity, Renal Proximal Tubule	27	1	1	545		Toxicidade renal. Renal proximal tubule cells which are derived from normal kidney cells freshly isolated from rat.			
49	Cytotoxicity, RAS-Dependent BJeLR Fibroblast	27	1	1	2610		Organism: Homo sapiens, human / Cell Type: fibroblast / Tissue: skin; foreskin / Disease: normal.			
50	Cytotoxicity, H-4-II-E	27	1	1	543		Organism: Rattus norvegicus, rat / Tissue: liver / Disease: hepatoma.			

Table S2: Biological activities better classified by the Naïve Bayes method for the group of twenty seven lignans analyzed. The column #Pairs represents the number of occurrences of compounds/models with values of **Pa-Pi greater or equal to 0.35**, with Pa-Pi minimum (pessimistic range) greater than zero. The column #model shows the total number of different models associated with activity class. The score was calculated by dividing the #Pairs by 27, and dividing by the root square of the number of models. The Best Model column shows one of the class models. The Activity column is related to the number of active and inactive compounds found in relation to the number of active and inactive compounds belonging to the same activity class.

Entry	Activity Class	#Pairs	#models	Score	Best model	Activity	Descrição	Targets	TTD-Disease(2017)
1	Anti-Inflammatory model	454	30	3.07	435020	act=4/5 inact=2/5	NF-kB activation. Many cellular pathways leading to activation of NF-kB-family transcription factors have been identified to be participating in host-defense, immunity, inflammation, and cancer.		
2	**Bacteria, Salmonella Typhimurium**	168	8	2.2	2839				
3	Mycobacterium tuberculosis	291	29	2	743073			bioA	
4	Plasmodium falciparum	377	53	1.92	2302	act=1/4 inact=1/3		lactate dehydrogenase	
5	Herpes simplex virus Virion Protein 16 (counterscreen)	154	9	1.9	624395	act=1/1 inact=3/4	Interfence with HTS	transactivating tegument protein VP16	
6	Virus, HIV-1	211	19	1.79	651939			Envelope surface glycoprotein gp160, precursor	
7	Fungus, Candida albicans	195	18	1.7	Fungus, Candida albicans	act=2/3 inact=0/1			
8	TOR pathway GFP-fusion proteins [Saccharomyces cerevisiae]	151	11	1.69	488808		TORC1 is a central hub of a signaling network that couples cues from hormones and growth factors (in mammalian cells), energy and stresses, and the abundance of nutrients, to cell growth and proliferation. Importantly, in this context, most negative regulators of mammalian TORC1 (mTORC1) have been previously identified as tumor suppressor gene products, while many positive regulators of mTORC1 have been identified as proto-oncoproteins and/or are found at elevated levels in tumor-derived cell lines.	RPL19A	
9	Trypanosoma	226	27	1.61	686980		Chagas' disease; Trypanolytic infections	phosphoglycerate kinase	
10	Antiparasitic, Caenorhabditis elegans	135	10	1.58	651563		Antiparasitic, Caenorhabditis elegans	Protein skinhead-1	
11	**Bacteria, Pseudomonas aeruginosa**	143	13	1.47	468996			Elastase	
12	sphingosine 1-phosphate receptor 1	96	6	1.45	1821		Agonists of S1P1 would be of interest in the enhancement of endothelial barriers and therefore	sphingosine 1-phosphate receptor 1	Acne vulgaris; Advanced non-

							potentially for the treatment of multiple sclerosis, transplant rejection and adult respiratory distress syndrome.		small cell lung cancer; Autoimmune diabetes; Cancer; Cardiovascular disorder; Cutaneous lupus erythematosus; Hepatocellular carcinoma; Multiple scierosis; Immune disorder; Inflammatory disease; Macular degeneration; Multiple scierosis; Primary progressive multiple sclerosis; Psoriasis; Rheumatoid arthritis
13	Hsf1 protein	87	5	1.44	435004		Function as a stress-inducible and DNA-binding transcription factor that plays a central role in the transcriptional activation of the heat shock response (HSR), leading to the expression of a large class of molecular chaperones heat shock proteins (HSPs) that protect cells from cellular insults' damage. Involved in stress-induced cancer cell proliferation in a IER5-dependent manner.	Hsf1 protein	Amyotrophic lateral sclerosis; Atrial fibrillation; Cancer; Diabetes; Diabetic neuropathy; Gastrointestinal cancers; Herpes simplex virus infection; Leukemia; Metastasis
14	**Bacteria, Staphylococcus aureus**	66	3	1.41	700				
15	Agonista, p53	63	3	1.35	720552	act=2/2 inact=2/2	p53, a tumor suppressor protein, is activated following cellular insult, including DNA damage and other cellular stresses. The activation of p53 regulates cell fate by inducing DNA repair, cell cycle arrest, apoptosis, or cellular senescence. The activation of p53, therefore, is a good indicator of DNA damage and other cellular stresses.	Cellular tumor antigen p53	Acute myeloid leukemia; Cancer; Colorectal cancer; Head and neck cancer; Hematological malignancies; Late-stage solid tumors; Oral cavity cancer; Renal artery disease; Solid tumours; Toxicity; Hearing disorder

16	miR-21	51	2	1.34	2508		miR-21-3p plays a crucial oncogenic role in cell metastasis during oral squamous cell carcinoma progression. High miR21 expression is associated with renal cell carcinoma. MIR21 promotes PTEN expression in colorectal cancer. mir-21 overexpression is associated with hepatocellular carcinoma. miR-21 overexpression promotes the migration and invasion of glioma cells. Sox2 is a crucial mediator in miR-21-induced migration and invasion in human glioma cells. miR-21/Sox2-induced migration/invasion of glioma cells is involved in the activation of the Wnt/beta-catenin signaling.		
17	GLI family zinc finger 1	71	4	1.31	651994		Glioma, B-cell lymphoma, sarcoma. Medulloblastoma, the most common pediatric brain tumor. Hh pathway dysregulation has been linked to tumors of the brain, skin, pancreas, breast, ovaries, and blood, and small molecules targeting this pathway are now being pursued as anti-cancer therapies.	Gli1	
18	sentrin-specific protease 8	85	6	1.29	488903		Potent and selective SENP8 chemical probes would provide invaluable tools to help elucidate the function of this therapeutically important enzyme, and may ultimately lead to the development of new anticancer therapies.	sentrin-specific protease 8	
19	Cytotoxicity, HEK293	199	33	1.28	488924	act=1/3 inact=0/9	Organism: Homo sapiens, human / Tissue: embryonic kidney . Toxicidade renal		
20	serine/threonine-protein kinase 33	60	3	1.28	2821		The serine/threonine kinase 33, STK33, has been identified and shown to be required for the survival and proliferation of mutant KRAS-dependent cells involved in cancer.	serine/threonine-protein kinase 33 isoform a	
21	regulator of G-protein signaling 4	88	7	1.23	492999		RGS4 has potential involvement in the pathology of pain where RGS4 mRNA is upregulated in response to nerve injury models of neuropathic pain [8]. Additionally, RGS4 may be involved in negative regulation of insulin release from pancreatic beta cells, indicated by enhanced glucose tolerance and insulin release in pancreatic beta-cell-specific RGS4 k/o mice. Also, reduced expression of RGS4 has been correlated with schizophrenia.	regulator of G-protein signaling 4 isoform 2	
22	muscleblind-like protein 1	47	2	1.23	493199		Dystrophia myotonica 1 (DM1). A muscular disorder characterized by myotonia, muscle wasting in the distal extremities, cataract, hypogonadism, defective endocrine functions, male baldness and cardiac arrhythmias.	muscleblind-like protein 1 isoform a	
23	Anticancer, Acute myelogenous leukemia	80	6	1.21	2068			tyrosine-protein phosphatase non-	

								receptor type 7 isoform 2	
24	Yeast Lifespan	73	5	1.21	809				
25	Steroid Receptor Coactivator 3	46	2	1.2	602166	act=2/2 inact=0/0	Given the central role that SRC-3 plays in breast and other cancers, the search for small molecule agents that target SRC-1 and SRC-3 represent an innovative and potentially effective strategy to identify agents to treat hormone-refractory breast cancers and other cancers where these coactivators are overexpressed. Compounds that target the function of steroid receptor coactivator 3 (SRC-3) protein promise to be different because cancer cells are less likely to bypass the comprehensive disruption of multiple growth factor signaling systems that result from the loss of SRC-3 function.	nuclear receptor coactivator 3 isoform a	
26	Virus, Herpes	45	2	1.18	435023		Virus, Herpes	LANA	
27	dual specificity protein phosphatase 3	83	7	1.16	2684		dual specificity protein phosphatase 3. The protein encoded by this gene is a member of the dual specificity protein phosphatase subfamily. These phosphatases inactivate their target kinases by dephosphorylating both the phosphoserine/threonine and phosphotyrosine residues. They negatively regulate members of the mitogen-activated protein (MAP) kinase superfamily (MAPK/ERK, SAPK/JNK, p38), which are associated with cellular proliferation and differentiation.	dual specificity protein phosphatase 3	
28	Mitochondria, permeability	54	3	1.15	743360		Mitochondria permeability Transition. In 1976, Hunter et al3 discovered a phenomenon called permeability transition (PT) as a result of the opening of a channel, the permeability transition pore (PTP), which results in a modification in permeability properties of the mitochondrial inner membrane (IM). Since PT description, major efforts have been accomplished to decipher its physiological role and, more recently, its role in cell death and pathology. This allowed its recognition as a potent pharmacological target for diseases associated with mitochondrial dysfunction and excessive cell death (eg, ischemia/reperfusion [I/R], heart failure, cardiotoxicity, cancer, and neurodegeneration.		
29	DNA damage-inducible transcript 3 protein	43	2	1.13	504437		diabetes, Alzheimer's disease, and Parkinson's disease, to hemophilia, lysosomal storage diseases, and alpha-1 antitrypsin deficiency.	DNA damage-inducible transcript 3 protein	
30	Cytotoxicity, MAGI-CCR5	43	2	1.13	504393		The parental MAGI cell line is a HeLa cell clone expressing human CD4 and HIV-LTR-ß gal. MAGI-		

							CCR-5 cells are a clone of MAGI cells that express the human chemokine receptor, CCR5		
31	ADMET, HIA	43	2	1.13	ADMET, HIA		Intestinal Absorption		
32	nuclear receptor subfamily 0 group B member 1	42	2	1.1	687017	act=3/3 inact=1/1		nuclear receptor subfamily 0 group B member 1	
33	Focal adhesion kinase 1	42	2	1.1	810		Non-receptor protein-tyrosine kinase that plays an essential role in regulating cell migration, adhesion, spreading, reorganization of the actin cytoskeleton, formation and disassembly of focal adhesions and cell protrusions, cell cycle progression, cell proliferation and apoptosis. Required for early embryonic development and placenta development. Required for embryonic angiogenesis, normal cardiomyocyte migration and proliferation, and normal heart development. Regulates axon growth and neuronal cell migration, axon branching and synapse formation; required for normal development of the nervous system. Plays a role in osteogenesis and differentiation of osteoblasts.		Cancer; Late-stage solid tumors; Mesothelioma; Solid tumours
34	Antimicrobial, E. coli	42	2	1.1	638		Antimicrobial		
35	ADP Fluorescence Polarization Displacement Assay (counterscreen)	42	2	1.1	2712		0		
36	Cytotoxicity, HepG2	159	29	1.09	720535		Organism: Homo sapiens, human / Tissue: liver / Disease: hepatocellular carcinoma. Toxicidade hepática. Drug-induced liver injury is a major health problem that impacts the pharmaceutical industry, drug regulatory agencies and doctors. Drug induced hepatotoxicity accounts for more than 50% of acute liver failure, and is the major cause of drug withdrawals. Because of the significant patient morbidity and mortality, many drugs have been withdrawn from the market, including bromfenac, ebrotidine, and troglitazone.		
37	Virus, Hepatitis C	72	6	1.09	2173			NS3, partial	
38	Cytotoxicity, NIH3T3	87	9	1.07	651742		Organism: Mus musculus, mouse / Cell Type: fibroblast / Tissue: embryo.		
39	SUMO1/sentrin specific peptidase 6	50	3	1.07	504492		Several studies implicated the role of various SENP isoforms in the development of various diseases, including prostate cancer, thyroid cancer, colon cancer, pancreatic cancer, atherosclerosis and heart diseases. Especially, numerous studies indicated the role of SENPs in prostate cancer development.	SUMO-1-specific protease	
40	Luciferase (counterscreen)	64	5	1.06	720522		Interfence with HTS	Photinus pyralis luciferase	

41	corticotropin releasing factor-binding protein	57	4	1.06	602180			corticotropin releasing factor-binding protein; corticotropin-releasing hormone receptor 2	
42	alkaline phosphatase, placental-like	57	4	1.06	493126		Alkaline phosphatase (EC 3.1.3.1) (APs) catalyze the hydrolysis of phosphomonoesters, releasing inorganic phosphate and alcohol. IAP expression is largely restricted to the gut, especially to the epithelial cells (enterocytes) of the small intestinal mucosa. While the biological implications of this inhibition are not known, these inhibitors have proven to be useful in the differential determination of AP isozymes as important diagnostic markers in many diseases.	alkaline phosphatase, placental-like preproprotein	
43	Cytotoxicity, isogenic chicken DT40 cell line	49	3	1.05	743015	act=1/6 inact=0/0	Identification of genotoxic compounds		
44	Microtubule-associated protein tau	40	2	1.05	1720		The microtubule-associated protein tau is an abundant protein in the axons of neurons that stabilizes microtubules. With its ability to modulate microtubule dynamics, tau contributes directly or indirectly, to key structural and regulatory cellular functions. Under pathological conditions, tau becomes sequestered into insoluble aggregates called neurofibrillary tangles. This phenomenon is believed to have pathological consequences by promoting axonal transport deficits that ultimately lead to synaptic dysfunction and neuronal loss.	Microtubule-associated protein tau	Alzheimer disease; Central nervous system disease; Cognitive disorders; Neurodegenerative disease
45	Mitochondria, disruptor membrane potential	63	5	1.04	Mitochondria, disruptor membrane potential	act=2/4 inact=0/0	Mitochondrial membrane potential (MMP), one of the parameters for mitochondrial function, is generated by mitochondrial electron transport chain that creates an electrochemical gradient by a series of redox reactions. This gradient drives the synthesis of ATP, a crucial molecule for various cellular processes. Measuring MMP in living cells is commonly used to assess the effect of chemicals on mitochondrial function. A homogenous cell-based MMP assay using a water soluble mitochondrial membrane potential sensor (Mito-MPS dye developed by BD Biosciences) was used to evaluate chemically induced mitochondrial toxicity.		
46	Plasmodium berghei	69	6	1.04	540269		Malaria	glucose-6-phosphate dehydrogenase-6-phospho-gluconolactonase	

47	Cystic Fibrosis	69	6	1.04	624344		Involved in the transport of chloride ions. May regulate bicarbonate secretion and salvage in epithelial cells by regulating the SLC4A7 transporter. Can inhibit the chloride channel activity of ANO1. Plays a role in the chloride and bicarbonate homeostasis during sperm epididymal maturation and capacitation.		Cystic fibrosis; Diarrhea; HIV-associated diarrhoea
48	Cytotoxicity, L929	39	2	1.02	463117		Organism: Mus musculus, mouse / Tissue: subcutaneous connective tissue; areolar and adipose /		
49	sentrin-specific protease 1	55	4	1.02	651690		A growing body of evidence indicates that, SUMOylation plays key homeostatic roles in the androgen mediated development and function of the prostate both by restraining the transcriptional activity of the androgen receptor (AR) and by inducing the normal process of senescence, which is a growth-arrest program that limits the lifespan of mammalian cells and prevents tumor progression. Notably, recent data indicates that advanced prostate cancer cells, which are notoriously resistant to current therapies, evade SUMO-mediated homeostatic mechanisms through the specific upregulation of the SUMO protease SENP1.	sentrin-specific protease 1	
50	Cytotoxicity, Vero Cells	106	15	1.01	1650		Organism: Cercopithecus aethiops / Tissue: kidney / Disease: normal		

Table S3: Best predict activity classes for lignans using both machine learning methods, SVM and Naïve Bayes. Activity classes were ordered according the best rank between the two methods and by the average rank. The columns Atives and Inactives concern the number of lignans that were experimentaly tested with some bioassay in the activity class. The EF (enrichment factor) column was calculated with the number of actives and inactives among classes selected and the total of actives and inactives among all classes.

RANK	Activity Class	SVM score	Bayes score	SVM rank	Bayes rank	Actives	Inactives	EF	Description
1	Anti-Inflammatory model	2.22	3.07	2	1	5	5	1.21	Several models targeting the following pathways: NFkappaB Translocation; NF-kB activation ; TNF inhibition ; STAT1 activation ; STAT3 activation ; Inhibition of TLR4 ; Inhibition of TLR4-MyD88 ; Inhibition of TLR2 ; Inhibition of TLR3-IRF3.
2	Cytotoxicity, Human Lymphoblastoid Cells	2.93	0.57	1	250	0	0	1.21	lymphoblastoid cell line
3	Bacteria, Salmonella Typhimurium	1.68	2.2	3	2	0	0	1.21	Bacteria, Salmonella Typhimurium
4	Mycobacterium tuberculosis	1.46	2	10	3	0	2	1.01	Mycobacterium tuberculosis
5	Plasmodium falciparum	1.49	1.92	8	4	4	3	1.14	Plasmodium falciparum
6	Ape1 Endonuclease	1.65	0.75	4	155	0	0	1.14	overexpression of APE1 in many cancers and resistance of these tumor cells to radio- and chemotherapy. Thus, targeting APE1 could improve the efficacy of current treatment paradigms by promoting selective sensitization or protection of diseased and normal cells, respectively.
7	Agonista, p53	1.6	1.35	5	15	2	2	1.15	p53, a tumor suppressor protein, is activated following cellular insult, including DNA damage and other cellular stresses. The activation of p53 regulates cell fate by inducing DNA repair, cell cycle arrest, apoptosis, or cellular senescence. The activation of p53, therefore, is a good indicator of DNA damage and other cellular stresses.
8	Herpes simplex virus Virion Protein 16 (counterscreen)	1.26	1.9	17	5	1	4	1.03	Interference with HTS
9	Virus, HIV-1	1.24	1.79	21	6	0	0	1.03	Virus, HIV-1
10	Genotoxicity, ATAD5	1.57	0.36	6	358	5	1	1.21	ATPase family AAA domain-containing protein 5. Involved in DNA damage response. Involved in a RAD9A-related damage checkpoint, a pathway that is important in determining whether DNA damage is compatible with cell survival or whether it requires cell elimination by apoptosis. Modulates the RAD9A interaction with BCL2 and thereby induces DNA damages-induced apoptosis.
11	GLI family zinc finger 1	1.54	1.31	7	17	0	0	1.21	Glioma, B-cell lymphoma, sarcoma. Medulloblastoma, the most common pediatric brain tumor. Hh pathway dysregulation has been linked to tumors of the brain, skin, pancreas, breast, ovaries, and blood, and small molecules targeting this pathway are now being pursued as anti-cancer therapies.
12	Fungus, Candida albicans	0.64	1.7	154	7	3	1	1.27	Fungus, Candida albicans
13	TOR pathway GFP-fusion proteins [Saccharomyces cerevisiae]	1.08	1.69	29	8	0	0	1.27	TORC1 is a central hub of a signaling network that couples cues from hormones and growth factors (in mammalian cells), energy and stresses, and the abundance of nutrients, to cell growth and proliferation. Most negative regulators of mammalian TORC1 (mTORC1) have been previously identified as tumor suppressor gene products, while many positive

									regulators of mTORC1 have been identified as proto-oncoproteins and/or are found at elevated levels in tumor-derived cell lines
14	Anticancer, RecQ-Like Dna Helicase 1 (RECQ1)	1.48	1.01	9	52	0	0	1.27	Human RECQ1 Is a DNA Damage Responsive Protein Required for Genotoxic Stress Resistance and Suppression of Sister Chromatid Exchanges. Developing drugs that interfere with DNA repair, which could sensitize cancer cells to conventional therapy.
15	Trypanosoma	0.66	1.61	145	9	0	0	1.27	Chagas' disease; Trypanolytic infections
16	Antiparasitic, Caenorhabditis elegans	0.97	1.58	59	10	0	0	1.27	Antiparasitic, Caenorhabditis elegans
17	Microphthalmia-associated transcription factor	1.4	0.94	11	73	0	0	1.27	MITF-related melanoma and renal cell carcinoma predisposition syndrome. nervous system disease, eye disease, genetic disorder, skin disease, metabolic disease
18	Bacteria, Pseudomonas aeruginosa	0.85	1.47	99	11	0	0	1.27	Bacteria, Pseudomonas aeruginosa
19	sphingosine 1-phosphate receptor 1	0.36	1.45	276	12	0	0	1.27	Agonists of S1P1 would be of interest in the enhancement of endothelial barriers and therefore potentially for the treatment of multiple sclerosis, transplant rejection and adult respiratory distress syndrome.
20	Angiogenesis model	1.37	0.39	12	345	2	1	1.3	Co-culture of endothelial colony forming cells (ECFC) with adipose-derived stromal cells (ADSC)
21	Cytotoxicity, isogenic chicken DT40 cell line	1.37	1.05	13	43	6	0	1.44	Identification of genotoxic compounds
22	Hsf1 protein	0.98	1.44	57	13	0	1	1.41	Function as a stress-inducible and DNA-binding transcription factor that plays a central role in the transcriptional activation of the heat shock response (HSR), leading to the expression of a large class of molecular chaperones heat shock proteins (HSPs) that protect cells from cellular insults' damage. Involved in stress-induced cancer cell proliferation in a IER5-dependent manner.
23	Bacteria, Staphylococcus aureus	0.86	1.41	95	14	0	0	1.41	Quorum sensing is a cell-to-cell communication system that permits members of a bacterial population to coordinate their behavior dependent on cell density. The mediators of this communication system are small, diffusible pheromones or autoinducers that are secreted by the bacteria and that accumulate extracellularly. At the appropriate concentration threshold that reflects a sufficient number or quorum of bacteria, the autoinducers signal gene expression programs that direct the coordinated action of the population. The list of bacterial pathogens that use this method of communication to regulate virulence is expanding and now includes some of the most common bacterial pathogens of humans including the medically important pathogen Staphylococcus aureus.
24	Cell Cycle Modulation (counterscreen)	1.34	0.26	14	428	2	0	1.45	Cell Cycle Modulation (counterscreen)
25	serine/threonine-protein kinase 33	1.28	1.28	15	20	0	0	1.45	The serine/threonine kinase 33, STK33, has been identified and shown to be required for the survival and proliferation of mutant KRAS-dependent cells involved in cancer.
26	muscleblind-like protein 1	1.28	1.23	16	22	0	0	1.45	Dystrophia myotonica 1 (DM1). A muscular disorder characterized by myotonia, muscle wasting in the distal extremities, cataract, hypogonadism, defective endocrine functions, male baldness and cardiac arrhythmias.
27	miR-21	0.65	1.34	150	16	0	0	1.45	miR-21-3p plays a crucial oncogenic role in cell metastasis during oral squamous cell carcinoma progression. High miR21 expression is associated with renal cell carcinoma. MIR21 promotes PTEN expression in colorectal cancer. mir-21 overexpression is associated with hepatocellular carcinoma. miR-21 overexpression promotes the migration and invasion of glioma cells. Sox2 is a crucial mediator in miR-21-induced migration and invasion in human glioma cells. miR-21/Sox2-induced migration/invasion of glioma cells is involved in the activation of the Wnt/beta-catenin signaling.

28	sentrin-specific protease 8	0.59	1.29	182	18	0	0	1.45	Potent and selective SENP8 chemical probes would provide invaluable tools to help elucidate the function of this therapeutically important enzyme, and may ultimately lead to the development of new anticancer therapies.
29	ADMET, Cytochrome P450 3A4	1.26	0.2	18	461	1	1	1.44	ADMET, Cytochrome P450 3A4
30	Cytotoxicity, HEK293	0.93	1.28	70	19	3	9	1.28	Organism: Homo sapiens, human / Tissue: embryonic kidney . Toxicidade renal
31	Osteoporosis model	1.26	0.43	19	315	3	0	1.33	Wnt potentiation module is to identify agents that may serve as novel medications for bone development and, specifically, osteoporosis
32	regulator of G-protein signaling 8	1.26	0.86	20	85	0	0	1.33	Since GPCRs control numerous physiologic processes in diverse tissues, including brain, heart, liver, and lung, modulation of the RGS/G protein interaction has become an attractive target for drug discovery.
33	regulator of G-protein signaling 4	0.91	1.23	84	21	0	0	1.33	RGS4 has potential involvement in the pathology of pain where RGS4 mRNA is upregulated in response to nerve injury models of neuropathic pain [8]. Additionally, RGS4 may be involved in negative regulation of insulin release from pancreatic beta cells, indicated by enhanced glucose tolerance and insulin release in pancreatic beta-cell-specific RGS4 k/o mice. Also, reduced expression of RGS4 has been correlated with schizophrenia.
34	Agonista, estrogen receptor alpha (ER-alpha)	1.24	0.15	22	506	0	2	1.29	Estrogen receptor alpha. Estrogen insensitivity syndrome is a very rare condition characterized by a defective ERα that is insensitive to estrogens. The clinical presentation of a female was observed to include absence of breast development and other female secondary sexual characteristics at puberty, hypoplastic uterus, primary amenorrhea, enlarged multicystic ovaries and associated lower abdominal pain, mild hyperandrogenism (manifested as cystic acne), and delayed bone maturation as well as an increased rate of bone turnover.
35	Anticancer, Acute myelogenous leukemia	0.48	1.21	216	23	0	0	1.29	Anticancer, Acute myelogenous leukemia
36	peripheral myelin protein 22	1.23	0	23	1000	2	0	1.33	Destabilization of myelin and neuropathic disorders, such as Charcot–Marie–Tooth type 1A (CMT1A), Dejerine–Sottas disease, and Hereditary Neuropathy with Liability to Pressure Palsy (HNPP)
37	Yeast Lifespan	0.5	1.21	208	24	0	0	1.33	Yeast Lifespan
38	Antagonista, estrogen receptor alpha (ER-alpha)	1.15	0.36	24	356	2	6	1.25	Estrogen receptor alpha. Estrogen insensitivity syndrome is a very rare condition characterized by a defective ERα that is insensitive to estrogens. The clinical presentation of a female was observed to include absence of breast development and other female secondary sexual characteristics at puberty, hypoplastic uterus, primary amenorrhea, enlarged multicystic ovaries and associated lower abdominal pain, mild hyperandrogenism (manifested as cystic acne), and delayed bone maturation as well as an increased rate of bone turnover.
39	Steroid Receptor Coactivator 3	0.75	1.2	118	25	2	0	1.28	Given the central role that SRC-3 plays in breast and other cancers, the search for small molecule agents that target SRC-1 and SRC-3 represent an innovative and potentially effective strategy to identify agents to treat hormone-refractory breast cancers and other cancers where these coactivators are overexpressed. Compounds that target the function of steroid receptor coactivator 3 (SRC-3) protein promise to be different because cancer cells are less likely to bypass the comprehensive disruption of multiple growth factor signaling systems that result from the loss of SRC-3 function.
40	1-acylglycerol-3-phosphate O-acyltransferase ABHD5	1.13	0.3	25	385	0	2	1.25	lipid disorders such as obesity, diabetes, and cardiovascular disease
41	ADP Fluorescence Polarization Displacement Assay (counterscreen)	1.13	1.1	26	35	0	0	1.25	ADP Fluorescence Polarization Displacement Assay (counterscreen)

42	Virus, Herpes	0.68	1.18	137	26	0	0	1.25	Virus, Herpes
43	dual specificity protein phosphatase 3	0.49	1.16	212	27	0	0	1.25	dual specificity protein phosphatase 3. The protein encoded by this gene is a member of the dual specificity protein phosphatase subfamily. These phosphatases inactivate their target kinases by dephosphorylating both the phosphoserine/threonine and phosphotyrosine residues. They negatively regulate members of the mitogen-activated protein (MAP) kinase superfamily (MAPK/ERK, SAPK/JNK, p38), which are associated with cellular proliferation and differentiation.
44	Giardia lamblia	1.09	0.64	27	220	0	0	1.25	Giardia lamblia
45	Anticancer, Leukemia	1.08	0.75	28	151	11	5	1.32	Anticancer, Leukemia
46	Mitochondria, permeability	0.09	1.15	443	28	0	0	1.32	Mitochondria permeability Transition. In 1976, Hunter et al3 discovered a phenomenon called permeability transition (PT) as a result of the opening of a channel, the permeability transition pore (PTP), which results in a modification in permeability properties of the mitochondrial inner membrane (IM). Since PT description, major efforts have been accomplished to decipher its physiological role and, more recently, its role in cell death and pathology. This allowed its recognition as a potent pharmacological target for diseases associated with mitochondrial dysfunction and excessive cell death (eg, ischemia/reperfusion [I/R], heart failure, cardiotoxicity, cancer, and neurodegeneration.
47	DNA damage-inducible transcript 3 protein	0.18	1.13	381	29	0	0	1.32	diabetes, Alzheimer's disease, and Parkinson's disease, to hemophilia, lysosomal storage diseases, and alpha-1 antitrypsin deficiency.
48	Antagonista, androgen receptor (AR)	1.08	0.67	30	212	4	4	1.31	Androgen receptor. The normal development and maintenance of the prostate is dependent on androgen acting through the androgen receptor (AR). AR remains important in the development and progression of prostate cancer. AR ligands are widely used in a variety of clinical applications (i.e., agonists are employed for hypogonadism, while antagonists are used for prostate cancer therapy).
49	Cytotoxicity, MAGI-CCR5	0	1.13	1000	30	0	0	1.31	The parental MAGI cell line is a HeLa cell clone expressing human CD4 and HIV-LTR-ß gal. MAGI-CCR-5 cells are a clone of MAGI cells that express the human chemokine receptor, CCR5
50	ADMET, HIA	0.24	1.13	335	31	0	0	1.31	Intestinal Absorption

Table S4: Bioassays used to validate the bioactivity predictions of lignans. All lignanas were excluded from the dataset before the model were built.

	PubChem Biossay					Modeling Information				
Entry	AID	Title	Source	Lignans		Activity Class	AUC SVM	AUC Bayes	Number of compounds	Number of actives
				Active	Inactive					
1	111	NCI human tumor cell line growth inhibition assay. Data for the P388 Leukemia cell line	DTP/NCI	3	0	Anticancer, Leukemia	0.78±0.025	0.687±0.029	620	153
2	117	NCI human tumor cell line growth inhibition assay. Data for the P388/ADR Leukemia cell line	DTP/NCI	3	0	Anticancer, Leukemia	0.745±0.028	0.648±0.032	614	138
3	127	NCI human tumor cell line growth inhibition assay. Data for the SN12K1 Renal cell line	DTP/NCI	3	0	Anticancer, Renal tumor	0.808±0.024	0.671±0.033	633	117
4	190	NCI In Vivo Anticancer Drug Screen. Data for tumor model Lymphoma AKR (Transplanted) (intraperitoneal) in AKR/Lw mice	DTP/NCI	0	3	Anticancer, Lymphoma	0.863±0.046	0.86±0.047	150	19

5	192	NCI In Vivo Anticancer Drug Screen. Data for tumor model B16 Melanoma (intraperitoneal) in B6D2F1 (BDF1) mice	DTP/NCI	0	5	Anticancer, Melanoma	0.846±0.013	0.719±0.02	3567	211
6	250	NCI In Vivo Anticancer Drug Screen. Data for tumor model L1210 Leukemia (subcutaneous) in B6D2F1 (BDF1) mice	DTP/NCI	0	1	Anticancer, Leukemia	0.723±0.046	0.731±0.045	199	87
7	258	NCI In Vivo Anticancer Drug Screen. Data for tumor model L1210 Leukemia (subcutaneous) in CD2F1 (CDF1) mice	DTP/NCI	0	3	Anticancer, Leukemia	0.737±0.062	0.714±0.064	113	62
8	264	NCI In Vivo Anticancer Drug Screen. Data for tumor model Human Lung LX-1 Xenograft (intrarenal inoculation) in NU/NU Swiss (nude) mice	DTP/NCI	2	0	Anticancer, Human Lung LX-1 Xenograft	0.45±0.044	0.53±0.042	446	97
9	270	NCI In Vivo Anticancer Drug Screen. Data for tumor model Lewis Lung Carcinoma (subcutaneous) in B6D2F1 (BDF1) mice	DTP/NCI	0	1	Anticancer, Lung Tumor	0.693±0.04	0.701±0.039	517	69
10	292	NCI In Vivo Anticancer Drug Screen. Data for tumor model Sarcoma M5076 (intraperitoneal) in B6C3F1 mice	DTP/NCI	2	0	Anticancer, Sarcoma	0.804±0.03	0.688±0.04	481	69
11	296	NCI In Vivo Anticancer Drug Screen. Data for tumor model Human Mammary Carcinoma MX-1 Xenograft (intrarenal inoculation) in NU/NU Swiss (nude) mice	DTP/NCI	0	2	Anticancer, Human Mammary Carcinoma	0.547±0.043	0.557±0.043	501	86
12	328	NCI In Vivo Anticancer Drug Screen. Data for tumor model P388 Leukemia (intraperitoneal) in B6D2F1 (BDF1) mice	DTP/NCI	5	0	Anticancer, Leukemia	0.839±0.008	0.722±0.011	7905	815
13	357	AP1 Signaling Pathway	NCGC	0	1	AP1 Signaling Pathway	0.757±0.038	0.539±0.056	8309	44
14	364	Cell Proliferation & Viability (Cytotoxicity) Assay	The Scripps Research Institute Molecular Screening Center	1	0	Cytotoxicity, Jurkat	0.751±0.041	0.522±0.059	2970	40
15	365	E. coli RNase H Inhibition	MTDP	1	0	ribonuclease H1, E. coli	0.722±0.049	0.74±0.047	183	92
16	366	human RNase H Inhibition	MTDP	1	0	ribonuclease H1, Human	0.66±0.068	0.825±0.056	183	145
17	367	HIV-2 RNase H Inhibition	MTDP	0	1	Virus, HIV-2	0.64±0.064	0.63±0.064	183	137
18	371	Human A549 Lung Tumor Cell Growth Inhibition Assay	SRMLSC	1	0	Cytotoxicity, A549	0.759±0.017	0.682±0.021	2972	239
19	438	Cellular assay for TNF alpha induced NFkappaB translocation	Columbia University Molecular Screening Center	1	0	Anti-Inflammatory model	0.582±0.047	0.462±0.051	11669	56
20	446	Stat Signaling Pathway	NCGC	0	1	Stat Signaling Pathway	0.752±0.038	0.491±0.056	8343	46
21	450	GR-GFP Redistribution	NCGC	0	1	glucocorticoid receptor (GR)	0.915±0.011	0.929±0.009	8883	102
22	457	VCAM-1 Imaging Assay in Pooled HUVECs: Augmentation of TNFa induced VCAM-1 cell surface expression.	Burnham Center for Chemical Genomics	0	1	Anti-Inflammatory model	0.66±0.071	0.695±0.067	9505	20
23	464	Cell Proliferation & Viability (Cytotoxicity) Dose Response Assay 60K MLSMR	The Scripps Research Institute	0	1	Cytotoxicity, Jurkat	0.638±0.027	0.673±0.026	683	317

			Molecular Screening Center							
24	483	Aggregation and Clearance of Mutant Huntingtin Protein	Columbia University Molecular Screening Center	0	1	Huntington disease protein	0.526±0.041	0.454±0.042	9489	83
25	575	Human Endothelial Cell Proliferation Assay	SRMLSC	0	1	Angiogenesis model	0.731±0.026	0.617±0.032	9346	106
26	599	Counterscreen for inhibitors of the nuclear receptor Steroidogenic Factor 1 (SF-1): A cell-based dose-response assay for inhibition of the RAR-related orphan receptor A (RORA)	The Scripps Research Institute Molecular Screening Center	1	0	Nuclear receptor ROR-alpha (counterscreen)	0.634±0.04	0.673±0.039	343	207
27	600	Dose-response cell-based assay for inhibitors of the nuclear receptor Steroidogenic Factor 1 (SF-1)	The Scripps Research Institute Molecular Screening Center	1	0	steroidogenic factor 1	0.62±0.04	0.592±0.04	345	203
28	608	NMR Based Screening Assay for FKBP12	Burnham Center for Chemical Genomics	0	4	peptidyl-prolyl cis-trans isomerase FKBP1A	0.867±0.024	0.628±0.051	3701	42
29	624	Measurement of GPCR-mediated thallium flux through GIRK channels: Primary Screen	Vanderbilt Screening Center for GPCRs, Ion Channels and Transporters	0	1	potassium voltage-gated channel subfamily J member 3	0.605±0.051	0.516±0.056	8078	44
30	636	Modulators of Post-Golgi Transport - 384-well pilot screen	SRMLSC	0	1	Post-Golgi Transport	0.714±0.052	0.447±0.072	9416	29
31	642	Allosteric Modulators of D1 Receptors: Confirmation Screen	Vanderbilt Screening Center for GPCRs, Ion Channels and Transporters	1	0	dopamine D1 receptor	0.744±0.024	0.854±0.02	2186	1929
32	647	Allosteric Modulators of D1 Receptors: Secondary Assay 2	Vanderbilt Screening Center for GPCRs, Ion Channels and Transporters	1	0	dopamine D1 receptor	0.649±0.016	0.715±0.015	2186	1389
33	771	Cell Growth High Content Screening Assay of Human HT29 Colon Tumor Cells (48 Hour Treatment Protocol)	SRMLSC	0	1	Anticancer, Colon Tumor	0.846±0.028	0.647±0.05	2972	41
34	884	qHTS Assay for Inhibitors and Substrates of Cytochrome P450 3A4	NCGC	1	0	ADMET, Cytochrome P450 3A4	0.921±0.004	0.87±0.005	8994	3112
35	885	qHTS Assay for Activators of Cytochrome P450 3A4	NCGC	0	1	ADMET, Cytochrome P450 3A4	0.907±0.009	0.823±0.016	11159	150
36	1013	Confirmatory Screen for chemical inhibitors of TNF alpha stimulated VCAM1 expression	Columbia University Molecular Screening Center	1	0	Anti-Inflammatory model	0.764±0.037	0.566±0.051	477	57
37	1249	Confirmatory Screen for chemical modifiers of cytoskeleton assembly	Columbia University	0	1	Anti-Inflammatory model	0.76±0.059	0.413±0.092	672	19

			Molecular Screening Center							
38	1267	Confirmation cell-based high throughput screening assay to measure STAT3 activation	The Scripps Research Institute Molecular Screening Center	0	1	Anti-Inflammatory model	0.572±0.023	0.591±0.023	1168	746
39	1309	Counterscreen assay for STAT3 activators: Cell-based high throughput assay to measure NF-kappaB activation	The Scripps Research Institute Molecular Screening Center	1	0	Anti-Inflammatory model	0.731±0.026	0.639±0.031	1168	127
40	1318	Counterscreen assay for STAT3 activators: Cell-based high throughput assay to measure STAT1 activation	The Scripps Research Institute Molecular Screening Center	0	1	Anti-Inflammatory model (counterscreen)	0.715±0.026	0.688±0.027	1168	150
41	1332	High Throughput Screen to Identify Inhibitors of Mycobacterium tuberculosis H37Rv	Southern Research Institute	0	2	Mycobacterium tuberculosis	0.873±0.016	0.773±0.023	1034	149
42	1650	A Cell Based Secondary Assay To Explore Cytotoxicity of West Nile Virus Anti-Viral Hit Compounds	Southern Research Specialized Biocontainment Screening Center	0	1	Cytotoxicity, Vero Cells	0.688±0.03	0.69±0.03	546	173
43	1834	Luminescence-based confirmation cell-based high throughput screening assay to identify inhibitors of kruppel-like factor 5 (KLF5)	The Scripps Research Institute Molecular Screening Center	1	0	Anticancer, colon	0.583±0.039	0.736±0.036	556	437
44	1905	Luminescence-based counterscreen assay for KLF5 inhibitors: cell-based high throughput screening assay to identify cytotoxic compounds using the IEC-6 intestinal epithelial cell line in triplicate.	The Scripps Research Institute Molecular Screening Center	1	0	Cytotoxicity, IEC-6 intestinal epithelial	0.826±0.023	0.799±0.025	556	297
45	1907	Luminescence-based confirmation cell-based assay for cytotoxic compounds using the IEC-6 intestinal epithelial cell line.	The Scripps Research Institute Molecular Screening Center	1	0	Cytotoxicity, IEC-6 intestinal epithelial	0.674±0.018	0.696±0.017	1744	1096
46	1990	Luminescence Cell-Based Dose Response HTS to Identify Inhibitors of Luciferase Translation or Activity in H4 Neuroglioblastoma Cells	Broad Institute	1	0	Counterscreen, Luciferase	0.586±0.019	0.559±0.019	1776	1131
47	2010	Luminescence Cell-Based Dose Response HTS Screen to Identify Cytotoxic Compounds of NIH3T3 cells.	Broad Institute	0	1	Cytotoxicity, NIH3T3	0.662±0.013	0.638±0.013	3177	881
48	2327	Fluorescence Cell-Based Secondary Assay for toxicity in mammalian fibroblasts	Broad Institute	1	0	Cytotoxicity, NIH3T3	0.607±0.022	0.56±0.023	1216	382
49	2423	Fluorescence Cell-Based Secondary Assay to Identify Inhibitors of Resistant C. albicans Growth in the Presence of Fluconazole	Broad Institute	1	0	Fungus, Candida albicans	0.679±0.024	0.697±0.023	1330	1010
50	2467	Fluorescence Cell-Based Retest of C. albicans Growth in the Presence of Fluconazole	Broad Institute	1	0	Fungus, Candida albicans	0.66±0.019	0.608±0.019	1529	963
51	434954	Luminescence Cell-Free Homogeneous Dose Retest to Identify Inhibitors of Glycogen Synthase Kinase-3 beta Activity	Broad Institute	1	0	glycogen synthase kinase-3 alpha	0.806±0.013	0.711±0.015	2089	564

52	435020	Single concentration confirmation of chemical inhibitors of T-cell specific antigen receptor-induced NF-kB activation	Burnham Center for Chemical Genomics	1	0	Anti-Inflammatory model	0.731±0.014	0.653±0.016	2511	601
53	449703	NOVARTIS: Inhibition of Plasmodium falciparum 3D7 (drug-susceptible) proliferation in erythrocyte-based infection assay	ChEMBL	1	0	Plasmodium falciparum	0.727±0.011	0.729±0.011	5046	3709
54	449704	NOVARTIS: Inhibition of Plasmodium falciparum W2 (drug-resistant) proliferation in erythrocyte-based infection assay	ChEMBL	1	0	Plasmodium falciparum	0.668±0.013	0.661±0.014	5035	4095
55	463189	96-well format Chlamydomonas reinhardtii Algae Gravitaxis Assay to measure the difference in the absorbance between the small compact plug of WT swimming algae versus the MUT algae lacking cilia.	University of Pittsburgh Molecular Library Screening Center	0	1	Chlamydomonas reinhardtii Algae Gravitaxis	0.667±0.027	0.647±0.028	3489	143
56	489033	Dose Response selectivity of uHTS chemical inhibitors of T-cell specific antigen receptor-induced NF-kB activation in a 697B cell line using a luminescence assay	Burnham Center for Chemical Genomics	0	1	Anti-Inflammatory model	0.794±0.023	0.715±0.027	603	203
57	489035	Dose response confirmation of uHTS chemical inhibitors of T-cell specific antigen receptor-induced NF-kB activation in a Jurkat cell line using a luminescence assay	Burnham Center for Chemical Genomics	0	1	Anti-Inflammatory model	0.703±0.027	0.647±0.029	603	250
58	489041	Dose response counterscreen of uHTS chemical inhibitors of T-cell specific antigen receptor-induced NF-kB activation in a HEK-293T cell line using a luminescence assay	Burnham Center for Chemical Genomics	1	0	NF-kB induction (counterscreen)	0.65±0.035	0.761±0.031	603	449
59	493027	Luminescence-based biochemical high throughput validation assay to identify inhibitors of the interaction of the lipase co-activator protein, abhydrolase domain containing 5 (ABHD5) with perilipin-1 (PLIN1)	The Scripps Research Institute Molecular Screening Center	0	1	1-acylglycerol-3-phosphate O-acyltransferase ABHD5	0.7±0.047	0.568±0.057	1629	40
60	493035	Luminescence-based biochemical high throughput validation assay to identify inhibitors of the interaction of the lipase co-activator protein, abhydrolase domain containing 5 (ABHD5) with perilipin-5 (MLDP; PLIN5)	The Scripps Research Institute Molecular Screening Center	0	1	1-acylglycerol-3-phosphate O-acyltransferase ABHD5	0.673±0.049	0.562±0.058	1629	40
61	493083	HSF-1 induced GFP reporter and Doxycycline induced RFP reporter Measured in Cell-Based System Using Plate Reader - 2038-03_Inhibitor_DoseNoFile_CherryPick_Activity_Set3	Broad Institute	0	1	Hsf1 protein	0.551±0.038	0.602±0.037	2265	91
62	504459	HTS for Beta-2AR agonists via FAP method from Validation Set	NMMLSC	0	1	beta-2 adrenergic receptor	0.552±0.072	0.552±0.073	1374	26
63	504592	Inhibitors of Prion Protein 5' UTR mRNA Measured in Cell-Based System Using Plate Reader - 2078-01_Inhibitor_SinglePoint_CherryPick_Activity	Broad Institute	1	0	Prion Protein 5' UTR mRNA	0.773±0.02	0.732±0.021	924	448
64	504848	Confirmation screen for delayed death inhibitors of the malarial parasite plastid, 96 hour incubation	NCGC	1	0	Plasmodium falciparum	0.73±0.025	0.761±0.025	1492	1253
65	504850	Confirmation screen for delayed death inhibitors of the malarial parasite plastid, 48 hour incubation	NCGC	1	0	Plasmodium falciparum	0.768±0.021	0.773±0.021	1386	1052
66	504861	Viability counterscreen of potential LMP-1 inhibitors in HEK293 cell background Measured in Cell-Based System Using Plate Reader - 2122-02_Inhibitor_Dose_CherryPick_Activity	Broad Institute	1	0	Cytotoxicity, HEK293	0.75±0.02	0.66±0.023	1113	257

67	504865	Inhibitors of USP1/UAF1: Pilot qHTS	NCGC	0	1	USP1 protein	0.86±0.011	0.687±0.02	5332	247
68	504882	Lymphoblastoid Cells (LCL) Cytotoxicity Secondary Assay Measured in Cell-Based System Using Plate Reader - 2122-03_Inhibitor_Dose_CherryPick_Activity_Set2	Broad Institute	1	0	Virus, Epstein-Barr	0.771±0.019	0.687±0.022	1039	268
69	521220	Inhibition of neurosphere proliferation of mouse neural precursor cells by MTT assay	ChEMBL	1	1	Citotoxidade, mouse neural precursor cells	0.624±0.043	0.515±0.048	644	67
70	537733	Binding affinity to Candida albicans CaCdr1p expressed in yeast AD1-8u	ChEMBL	1	0	Fungus, Candida albicans	0.535±0.102	0.776±0.084	56	31
71	588343	Inhibitors of Epstein-Barr LMP1 inducible NF-kappaB luciferase reporter Measured in Cell-Based System Using Plate Reader - 2122-01_Inhibitor_Dose_CherryPick_Activity	Broad Institute	1	0	Virus, Epstein-Barr	0.768±0.019	0.721±0.02	1084	604
72	588350	Counterscreen for activators of the GAA850 frataxin promoter: luminescence-based cell-based high throughput screening assay to identify activators of the GAA30 frataxin promoter	The Scripps Research Institute Molecular Screening Center	0	1	GAA30 promoter (counterscreen)	0.769±0.017	0.755±0.017	1742	1157
73	588398	TES1 - eGFP vs TES2 -dsRED Pathway Differentiation Measured in Cell-Based System Using Imaging - 2122-04_Inhibitor_Dose_CherryPick_Activity	Broad Institute	1	0	Virus, Epstein-Barr	0.621±0.036	0.537±0.039	1054	99
74	588506	Phenotypic HTS multiplex for antifungal efflux pump inhibitors with Validation compound Set	NMMLSC	0	1	Fungus, Candida albicans	0.46±0.04	0.5±0.038	1638	100
75	588792	Luminescence-based cell-based high throughput confirmation assay for inhibitors of the Steroid Receptor Coactivator 3 (SRC3; NCOA3)	The Scripps Research Institute Molecular Screening Center	1	0	Steroid Receptor Coactivator 3	0.728±0.015	0.673±0.016	1923	687
76	588794	Counterscreen for inhibitors of the Steroid Receptor Coactivator 3 (SRC3; NCOA3): Luminescence-based cell-based high throughput assay to identify inhibitors of the Herpes Virus Virion Protein 16 (VP16)	The Scripps Research Institute Molecular Screening Center	0	1	Herpes simplex virus Virion Protein 16 (counterscreen)	0.667±0.016	0.616±0.017	1923	926
77	588820	Luminescence-based cell-based high throughput confirmation assay for inhibitors of the Steroid Receptor Coactivator 1 (SRC1; NCOA1).	The Scripps Research Institute Molecular Screening Center	1	0	Steroid Receptor Coactivator 1	0.727±0.017	0.709±0.017	1996	373
78	588824	Counterscreen for inhibitors of the Steroid Receptor Coactivator 1 (SRC1; NCOA1): Luminescence-based cell-based high throughput assay to identify inhibitors of the Herpes Virus Virion Protein 16 (VP16).	The Scripps Research Institute Molecular Screening Center	0	1	Herpes simplex virus Virion Protein 16 (counterscreen)	0.678±0.016	0.632±0.016	1996	878
79	602156	Novartis GNF Liver Stage Dataset: Malariabox Annotation	GNF / Scripps Winzeler lab	0	1	Plasmodium falciparum	0.749±0.016	0.65±0.02	5543	267
80	602166	Luminescence-based cell-based high throughput dose response assay for inhibitors of the Steroid Receptor Coactivator 3 (SRC3; NCOA3)	The Scripps Research Institute Molecular Screening Center	1	0	Steroid Receptor Coactivator 3	0.636±0.048	0.652±0.048	218	111
81	602168	Counterscreen for inhibitors of the Steroid Receptor Coactivator 3 (SRC3; NCOA3): Luminescence-based cell-based high throughput dose response assay to identify	The Scripps Research Institute	0	1	Steroid Receptor Coactivator 1 (counterscreen)	0.55±0.064	0.6±0.061	218	41

		inhibitors of the Steroid Receptor Coactivator 1 (SRC1; NCOA1)	Molecular Screening Center							
82	602234	Counterscreen for inhibitors of the Steroid Receptor Coactivator 1 (SRC1; NCOA1): Luminescence-based cell-based high throughput dose response assay to identify inhibitors of the Steroid Receptor Coactivator 3 (SRC3; NCOA3)	The Scripps Research Institute Molecular Screening Center	1	0	Steroid Receptor Coactivator 3 (counterscreen)	0.568±0.058	0.477±0.057	222	164
83	602235	Luminescence-based cell-based high throughput dose response assay for inhibitors of the Steroid Receptor Coactivator 1 (SRC1; NCOA1)	The Scripps Research Institute Molecular Screening Center	1	0	Steroid Receptor Coactivator 1	0.659±0.052	0.638±0.053	222	53
84	602277	Novel Modifiers of Toll-like and RIG-like Receptor Signaling-Poly IC Stimulus	University of Pittsburgh Molecular Library Screening Center	1	0	Anti-Inflammatory model	0.608±0.022	0.516±0.024	9512	239
85	624002	qHTS for Inhibitors of mutant isocitrate dehydrogenase 1 (IDH1): Confirmation of Cherrypicks	NCGC	1	0	Anticancer, glioblastoma	0.706±0.063	0.893±0.045	634	596
86	624030	Biochemical firefly luciferase enzyme assay for NPC	NCGC	0	1	Counterscreen, Luciferase	0.859±0.019	0.765±0.028	2688	83
87	624031	S16 Schwann cell viability assay (CellTiter-Glo assay)	NCGC	0	1	Cytotoxicity, S16	0.8±0.028	0.715±0.036	1901	65
88	624032	S16 Schwann cell PMP22 intronic element firefly luciferase assay	NCGC	1	0	peripheral myelin protein 22	0.686±0.023	0.687±0.022	1830	208
89	624044	S16 Schwann cell PMP22 intronic element beta-lactamase assay	NCGC	1	0	peripheral myelin protein 22	0.687±0.035	0.495±0.044	1899	76
90	624378	Luminescence-based cell-based high throughput confirmation assay for agonists of nuclear receptor subfamily 2, group E, member 3 (NR2E3)	The Scripps Research Institute Molecular Screening Center	1	0	photoreceptor-specific nuclear receptor	0.674±0.015	0.628±0.015	2153	1058
91	624379	Counterscreen for agonists of nuclear receptor subfamily 2, group E, member 3 (NR2E3): Luminescence-based cell-based high throughput screening assay to identify agonists of the Herpes Virus Virion Protein 16 (VP16)	The Scripps Research Institute Molecular Screening Center	0	1	Herpes simplex virus Virion Protein 16 (counterscreen)	0.681±0.016	0.605±0.017	2153	595
92	624394	Luminescence-based cell-based high throughput dose response assay for agonists of nuclear receptor subfamily 2, group E, member 3 (NR2E3)	The Scripps Research Institute Molecular Screening Center	1	0	photoreceptor-specific nuclear receptor	0.453±0.085	0.786±0.08	207	186
93	624395	Counterscreen for agonists of nuclear receptor subfamily 2, group E, member 3 (NR2E3):Luminescence-based cell-based high throughput dose response assay to identify inhibitors of the Herpes Virus Virion Protein 16 (VP16)	The Scripps Research Institute Molecular Screening Center	1	0	Herpes simplex virus Virion Protein 16 (counterscreen)	0.592±0.055	0.625±0.054	207	136
94	624476	Cytotoxicity counterscreen for NFkB agonists and antagonists	NCGC	0	1	Cytotoxicity	0.655±0.04	0.657±0.04	324	102
95	624479	qHTS Assay for Identification of Small Molecule Antagonists for NFkB Signaling Pathway.	NCGC	0	1	Antagonist, NFkB Signaling Pathway	0.685±0.037	0.695±0.037	334	153
96	651558	qHTS Assay to Find Inhibitors of Chronic Active B-Cell Receptor Signaling: Hit Validation in Primary Screen	NCGC	1	0	Anticancer, non-Hodgkin lymphoma	0.794±0.021	0.784±0.021	837	460

97	651568	qHTS Assay to Find Inhibitors of Chronic Active B-Cell Receptor Signaling: Hit Validation in Renilla Reporter Assay	NCGC	0	1	Anticancer, non-Hodgkin lymphoma	0.791±0.033	0.635±0.048	1001	50
98	651611	Counterscreen for inverse agonists of the liver receptor homolog-1 (LRH-1; NR5A2): Luminescence-based cell-based high throughput assay to identify nonselective inhibitors of the Steroidogenic acute regulatory protein (StAR) promoter or luminescence assay artifacts	The Scripps Research Institute Molecular Screening Center	1	0	Steroidogenic acute regulatory protein (StAR) promoter (counterscreen)	0.786±0.013	0.713±0.015	1998	758
99	651613	Luminescence-based cell-based high throughput confirmation assay for inverse agonists of the liver receptor homolog-1 (LRH-1; NR5A2)	The Scripps Research Institute Molecular Screening Center	1	0	nuclear receptor subfamily 5 group A member 2	0.608±0.022	0.629±0.022	1998	1623
100	651615	Counterscreen for inverse agonists of the liver receptor homolog-1 (LRH-1; NR5A2): Luminescence-based cell-based high throughput assay to identify inhibitors of the Herpes Virus Virion Protein 16 (VP16)	The Scripps Research Institute Molecular Screening Center	0	1	Herpes simplex virus Virion Protein 16 (counterscreen)	0.805±0.012	0.727±0.015	1998	699
101	651629	Single concentration confirmation of small molecule Triacylglycerol inhbitors in a fluoresence assay	Burnham Center for Chemical Genomics	1	0	Triacylglycerol inhbitors	0.618±0.027	0.613±0.027	876	611
102	651630	Dose Response confirmation of small molecule Triacylglycerol inhbitors in a panel assay	Burnham Center for Chemical Genomics	0	1	Triacylglycerol inhbitors	0.636±0.03	0.582±0.031	611	193
103	651631	qHTS assay for small molecule agonists of the p53 signaling pathway	Tox21	1	1	Agonist, p53	0.779±0.014	0.642±0.019	4451	335
104	651632	qHTS assay for small molecules that induce genotoxicity in human embryonic kidney cells expressing luciferase-tagged ATAD5	Tox21	2	0	Genotoxicity, ATAD5	0.789±0.02	0.625±0.029	5619	131
105	651633	qHTS assay for small molecule agonists of the p53 signaling pathway - cell viability	Tox21	0	2	Cytotoxicity, Agonist, p53	0.802±0.012	0.679±0.017	5510	335
106	651634	qHTS assay for small molecules that induce genotoxicity in human embryonic kidney cells expressing luciferase-tagged ATAD5 - cell viability	Tox21	1	1	Genotoxicity, ATAD5	0.784±0.015	0.637±0.021	5500	254
107	651712	Cell Proliferation Assay against the TMD8 Cell Line (Caspase readout at 16 hrs)	NCGC	1	0	Cytotoxicity, TMD8	0.644±0.047	0.659±0.046	249	77
108	652134	Luminescence-based cell-based primary high throughput confirmation assay for inhibitors of the orphan nuclear receptor subfamily 0, group B, member 1 (DAX1; NR0B1): repression of SF-1 (NR5A1) activated StAR promoter by full-length DAX-1	The Scripps Research Institute Molecular Screening Center	2	0	nuclear receptor subfamily 0 group B member 1	0.766±0.021	0.865±0.017	2378	2063
109	652136	Counterscreen for inhibitors of the orphan nuclear receptor subfamily 0, group B, member 1 (DAX1; NR0B1): Luminescence-based cell-based high throughput assay for nonselective inhibitors/assay artifacts using AP2 mutant SF-1 (NR5A1) Transactivation Assay	The Scripps Research Institute Molecular Screening Center	0	2	steroidogenic factor 1 (counterscreen)	0.727±0.018	0.821±0.016	2378	1857
110	686947	qHTS for small molecule inhibitors of Yes1 kinase: Primary Screen	NCGC	0	1	Anticancer, Yes1 kinase	0.83±0.02	0.793±0.022	682	337
111	687017	Luminescence-based cell-based high throughput dose response assay for inhibitors of the orphan nuclear	The Scripps Research Institute	1	1	nuclear receptor subfamily 0 group B member 1	0.554±0.058	0.575±0.057	234	51

		receptor subfamily 0, group B, member 1 (DAX1; NR0B1): repression of SF-1 (NR5A1) activated StAR promoter by full-length DAX-1	Molecular Screening Center							
112	687029	PAX8: PAX8-dependent cytoxicity Measured in Cell-Based System Using Plate Reader - 7054-06_Inhibitor_Dose_CherryPick_Activity	Broad Institute	0	1	Cytotoxicity, RMG-1 (PAX8)	0.615±0.031	0.755±0.024	1494	131
113	720516	qHTS assay for small molecules that induce genotoxicity in human embryonic kidney cells expressing luciferase-tagged ATAD5: Summary	Tox21	2	0	Genotoxicity, ATAD5	0.805±0.015	0.637±0.023	5612	203
114	720552	qHTS assay for small molecule agonists of the p53 signaling pathway: Summary	Tox21	1	1	Agonist, p53	0.736±0.014	0.621±0.018	5393	367
115	720634	qHTS assay for small molecule disruptors of the mitochondrial membrane potential - cell viability	Tox21	0	2	Cytotoxicity, Mitochondria, membrane potential	0.778±0.017	0.713±0.02	5381	207
116	720635	qHTS assay for small molecule disruptors of the mitochondrial membrane potential	Tox21	2	0	Mitochondria, disruptor membrane potential	0.877±0.007	0.82±0.008	3793	1059
117	720637	qHTS assay for small molecule disruptors of the mitochondrial membrane potential: Summary	Tox21	2	0	Mitochondria, disruptor membrane potential	0.869±0.007	0.813±0.009	4637	741
118	720678	qHTS assay to test for compound auto fluorescence at 460 nm (blue) in HEK293 cells	Tox21	0	2	Interference, Auto Fluorescence in HEK293	0.729±0.058	0.49±0.082	5775	22
119	720681	qHTS assay to test for compound auto fluorescence at 460 nm (blue) in HEK293 cell free culture	Tox21	0	2	Interference, Auto Fluorescence in HEK293	0.776±0.048	0.535±0.075	5782	25
120	720685	qHTS assay to test for compound auto fluorescence at 460 nm (blue) in HepG2 cell free culture	Tox21	0	2	Interference, Auto Fluorescence in HepG2	0.768±0.052	0.562±0.078	5777	22
121	720687	qHTS assay to test for compound auto fluorescence at 460 nm (blue) in HepG2 cells	Tox21	0	2	Interference, Auto Fluorescence in HepG2	0.706±0.063	0.509±0.083	5778	21
122	720691	qHTS assay to identify small molecule agonists of the glucocorticoid receptor (GR) signaling pathway	Tox21	0	2	Agonist, glucocorticoid receptor (GR)	0.85±0.016	0.73±0.025	5349	119
123	720693	qHTS assay to identify small molecule antagonists of the glucocorticoid receptor (GR) signaling pathway - cell viability counter screen	Tox21	0	2	Cytotoxicity, Antagonist, glucocorticoid receptor (GR)	0.759±0.019	0.59±0.026	5384	182
124	720719	qHTS assay to identify small molecule agonists of the glucocorticoid receptor (GR) signaling pathway: Summary	Tox21	0	2	Agonist, glucocorticoid receptor (GR)	0.849±0.016	0.734±0.025	5349	119
125	743012	qHTS assay for identifying genotoxic compounds that show differential cytotoxicity against isogenic chicken DT40 cell lines with known DNA damage response pathways - wild type cell line	Tox21	2	0	Cytotoxicity, isogenic chicken DT40 cell line	0.833±0.007	0.729±0.01	4956	1211
126	743014	qHTS assay for identifying genotoxic compounds that show differential cytotoxicity against isogenic chicken DT40 cell lines with known DNA damage response pathways - Rev3 mutant cell line	Tox21	2	0	Cytotoxicity, isogenic chicken DT40 cell line	0.802±0.008	0.716±0.01	4842	1278
127	743015	qHTS assay for identifying genotoxic compounds that show differential cytotoxicity against isogenic chicken DT40 cell lines with known DNA damage response pathways - Rad54/Ku70 mutant cell line	Tox21	2	0	Cytotoxicity, isogenic chicken DT40 cell line	0.832±0.008	0.725±0.01	5044	1295

128	743021	PAX8: non-specific cytotoxicity Measured in Cell-Based System Using Plate Reader - 7054-05_Inhibitor_Dose_DryPowder_Activity	Broad Institute	0	1	Cytotoxicity, RMG-1 (PAX8)	0.693±0.02	0.739±0.018	1546	295
129	743033	qHTS assay to identify small molecule antagonists of the androgen receptor (AR) signaling pathway - cell viability counter screen	Tox21	0	1	Cytotoxicity, Antagonist, androgen receptor (AR)	0.734±0.018	0.636±0.022	5259	228
130	743035	qHTS assay to identify small molecule antagonists of the androgen receptor (AR) signaling pathway	Tox21	2	0	Antagonist, androgen receptor (AR)	0.803±0.012	0.715±0.015	4304	402
131	743040	qHTS assay to identify small molecule agonists of the androgen receptor (AR) signaling pathway using the MDA cell line	Tox21	0	2	Agonist, androgen receptor (AR)	0.838±0.013	0.745±0.018	5663	228
132	743041	qHTS assay to identify small molecule antagonists of the androgen receptor (AR) signaling pathway using the MDA cell line - cell viability counter screen	Tox21	0	1	Cytotoxicity, Antagonist, androgen receptor (AR)	0.754±0.02	0.61±0.027	5379	165
133	743042	qHTS assay to identify small molecule antagonists of the androgen receptor (AR) signaling pathway using the MDA cell line	Tox21	0	2	Antagonist, androgen receptor (AR)	0.748±0.015	0.67±0.017	5383	342
134	743063	qHTS assay to identify small molecule antagonists of the androgen receptor (AR) signaling pathway: Summary	Tox21	2	0	Antagonist, androgen receptor (AR)	0.857±0.01	0.712±0.016	4826	365
135	743064	qHTS assay to identify small molecule antagonists of the thyroid receptor (TR) signaling pathway - cell viability counter screen	Tox21	2	0	Cytotoxicity, Antagonist, thyroid receptor (TR)	0.816±0.009	0.714±0.012	4932	668
136	743065	qHTS assay to identify small molecule antagonists of the thyroid receptor (TR) signaling pathway	Tox21	2	0	Antagonist, thyroid receptor (TR)	0.83±0.008	0.707±0.011	5278	935
137	743066	qHTS assay to identify small molecule agonists of the thyroid receptor (TR) signaling pathway	Tox21	0	1	Agonist, thyroid receptor (TR)	0.707±0.052	0.568±0.065	5422	30
138	743067	qHTS assay to identify small molecule antagonists of the thyroid receptor (TR) signaling pathway: Summary	Tox21	1	0	Antagonist, thyroid receptor (TR)	0.774±0.016	0.594±0.023	4385	248
139	743069	qHTS assay to identify small molecule antagonists of the estrogen receptor alpha (ER-alpha) signaling pathway	Tox21	0	2	Antagonist, estrogen receptor alpha (ER-alpha)	0.757±0.017	0.648±0.022	4819	229
140	743074	qHTS assay to identify small molecule antagonists of the estrogen receptor alpha (ER-alpha) signaling pathway - cell viability counter screen	Tox21	0	1	Cytotoxicity, Antagonist, estrogen receptor alpha (ER-alpha)	0.767±0.022	0.568±0.033	5118	123
141	743078	qHTS assay to identify small molecule antagonists of the estrogen receptor alpha (ER-alpha) signaling pathway: Summary	Tox21	0	2	Antagonist, estrogen receptor alpha (ER-alpha)	0.767±0.017	0.658±0.021	4819	229
142	743079	qHTS assay to identify small molecule agonists of the estrogen receptor alpha (ER-alpha) signaling pathway using the BG1 cell line	Tox21	0	2	Agonist, estrogen receptor alpha (ER-alpha)	0.781±0.011	0.727±0.013	4835	567
143	743080	qHTS assay to identify small molecule antagonists of the estrogen receptor alpha (ER-alpha) signaling pathway using the BG1 cell line	Tox21	1	0	Antagonist, estrogen receptor alpha (ER-alpha)	0.78±0.016	0.651±0.022	4945	232
144	743081	qHTS assay to identify small molecule antagonists of the estrogen receptor alpha (ER-alpha) signaling pathway using the BG1 cell line - cell viability counter screen	Tox21	0	1	Cytotoxicity, Antagonist, estrogen receptor alpha (ER-alpha)	0.869±0.015	0.556±0.035	5320	110
145	743083	qHTS assay to identify aromatase inhibitors	Tox21	0	2	Inhibitors, Aromatase	0.78±0.012	0.709±0.015	5407	405

146	743084	qHTS assay to identify aromatase inhibitors - cell viability counter screen	Tox21	0	2	Cytotoxicity, inhibitors, aromatase	0.779±0.015	0.649±0.02	5329	286
147	743086	qHTS assay to identify small molecule that activate the aryl hydrocarbon receptor (AhR) signaling pathway - cell viability counter screen	Tox21	1	1	Cytotoxicity, Activate, aryl hydrocarbon receptor (AhR)	0.703±0.023	0.567±0.029	5406	156
148	743091	qHTS assay to identify small molecule antagonists of the estrogen receptor alpha (ER-alpha) signaling pathway using the BG1 cell line: Summary	Tox21	1	0	Antagonist, estrogen receptor alpha (ER-alpha)	0.778±0.016	0.643±0.022	4945	232
149	743122	qHTS assay to identify small molecule that activate the aryl hydrocarbon receptor (AhR) signaling pathway: Summary	Tox21	2	0	Activate, aryl hydrocarbon receptor (AhR)	0.842±0.009	0.816±0.009	5134	666
150	743139	qHTS assay to identify aromatase inhibitors: Summary	Tox21	0	2	Inhibitors, Aromatase	0.795±0.017	0.66±0.024	4539	187
151	743191	qHTS assay to identify small molecule antagonists of the peroxisome proliferator-activated receptor gamma (PPARg) signaling pathway	Tox21	0	1	Antagonist, peroxisome proliferator-activated receptor gamma (PPARg)	0.77±0.022	0.537±0.034	3473	123
152	743194	qHTS assay to identify small molecule antagonists of the peroxisome proliferator-activated receptor gamma (PPARg) signaling pathway - cell viability counter screen	Tox21	0	1	Cytotoxicity, Antagonist, peroxisome proliferator-activated receptor gamma (PPARg)	0.791±0.015	0.649±0.021	4930	238
153	743203	qHTS assay for small molecule agonists of the antioxidant response element (ARE) signaling pathway - cell viability counter screen	Tox21	0	1	Cytotoxicity, Agonist, antioxidant response element (ARE)	0.777±0.015	0.646±0.02	5155	264
154	743209	qHTS assay for small molecule activators of the heat shock response signaling pathway - cell viability counter screen	Tox21	0	1	Cytotoxicity, Activators, heat shock response (counterscreen)	0.765±0.018	0.631±0.025	5097	186
155	743210	qHTS assay for small molecule activators of the heat shock response signaling pathway	Tox21	0	1	Activators, heat shock response	0.622±0.06	0.468±0.07	4682	31
156	743211	qHTS assay to identify small molecule agonists of the peroxisome proliferator-activated receptor delta (PPARd) signaling pathway - cell viability counter screen	Tox21	0	1	Cytotoxicity, Agonist, peroxisome proliferator-activated receptor delta (PPARd)	0.795±0.013	0.72±0.016	4962	331
157	743215	qHTS assay to identify small molecule antagonists of the peroxisome proliferator-activated receptor delta (PPARd) signaling pathway	Tox21	0	1	Antagonist, peroxisome proliferator-activated receptor delta (PPARd)	0.706±0.031	0.602±0.037	4419	86
158	743218	qHTS assay to identify small molecule agonists of the farnesoid-X-receptor (FXR) signaling pathway - cell viability counter screen	Tox21	0	1	Cytotoxicity, Agonist, farnesoid-X-receptor (FXR)	0.775±0.016	0.652±0.022	4713	227
159	743220	qHTS assay to identify small molecule agonists of the farnesoid-X-receptor (FXR) signaling pathway	Tox21	0	1	Agonist, farnesoid-X-receptor (FXR)	0.7±0.064	0.359±0.087	4897	21
160	743221	qHTS assay to identify small molecule antagonists of the farnesoid-X-receptor (FXR) signaling pathway - cell viability counter screen	Tox21	0	1	Cytotoxicity, Antagonist, farnesoid-X-receptor (FXR)	0.766±0.02	0.595±0.028	5137	152
161	743224	qHTS assay to identify small molecule agonists of the vitamin D receptor (VDR) signaling pathway - cell viability counter screen	Tox21	0	1	Cytotoxicity, Agonist, vitamin D receptor (VDR)	0.722±0.016	0.625±0.02	4992	304

162	743225	qHTS assay to identify small molecule antagonists of the vitamin D receptor (VDR) signaling pathway - cell viability counter screen	Tox21	0	1	Cytotoxicity, Antagonist, vitamin D receptor (VDR)	0.794±0.016	0.608±0.025	4838	195
163	743227	qHTS assay to identify small molecule agonists of the peroxisome proliferator-activated receptor delta (PPARd) signaling pathway: Summary	Tox21	0	1	Agonist, peroxisome proliferator-activated receptor delta (PPARd)	0.789±0.032	0.565±0.051	5107	50
164	743228	qHTS assay for small molecule activators of the heat shock response signaling pathway: Summary	Tox21	0	1	Activators, heat shock response	0.732±0.017	0.647±0.02	5097	279
165	743239	qHTS assay to identify small molecule agonists of the farnesoid-X-receptor (FXR) signaling pathway: Summary	Tox21	0	1	Agonist, farnesoid-X-receptor (FXR)	0.714±0.035	0.409±0.048	5308	66
166	743240	qHTS assay to identify small molecule antagonists of the farnesoid-X-receptor (FXR) signaling pathway: Summary	Tox21	1	0	Antagonist, farnesoid-X-receptor (FXR)	0.749±0.02	0.574±0.028	4874	165
167	743242	qHTS assay to identify small molecule antagonists of the vitamin D receptor (VDR) signaling pathway: Summary	Tox21	1	0	Antagonist, vitamin D receptor (VDR)	0.668±0.046	0.366±0.058	4835	45
168	743244	qHTS Assay for Identifying Gametocytocidal Compounds	NCGC	0	2	Plasmodium falciparum	0.827±0.014	0.719±0.019	3056	243
169	743471	HepG2 Cytotoxicity Assay Measured in Cell-Based System Using Plate Reader - 7071-02_Inhibitor_Dose_DryPowder_Activity_Set12	Broad Institute	0	1	Cytotoxicity, HepG2	0.584±0.086	0.7±0.077	76	31
170	743472	A549 Cytotoxicity Assay Measured in Cell-Based System Using Plate Reader - 7071-06_Inhibitor_Dose_DryPowder_Activity_Set12	Broad Institute	0	1	Cytotoxicity, A549	0.674±0.087	0.667±0.087	75	48
171	1053195	Cell Proliferation Assay Versus the ED40515 IL2 Dependent ATL cell lines	NCGC	0	1	Anticancer, Leukemia	0.575±0.038	0.597±0.037	428	136
172	1117304	qHTS Assay for Identifying Compounds that block Entry of Ebola Virus, Screen 2 blue channel	NCGC	1	0	Virus, Ebola	0.846±0.013	0.815±0.015	1390	348
173	1117305	qHTS Assay for Identifying Compounds that block Entry of Ebola Virus, Screen 2 ratio channel	NCGC	1	0	Virus, Ebola	0.82±0.016	0.81±0.016	1265	293
174	1117312	qHTS Assay for Identifying Compounds that block Entry of Ebola Virus: Screen2, ratio channel	NCGC	1	0	Virus, Ebola	0.836±0.013	0.807±0.014	1822	371
175	1117325	nuclear beta catenin stimulation in WNT3A conditioned C2C12 cells-IC50	Cheminformatics & Chemogenomics Research Group (CCRG), Indiana University School of Informatics	1	0	Osteoporosis model	0.627±0.093	0.66±0.09	61	30
176	1117326	nuclear beta catenin stimulation in WNT3A conditioned C2C12 cells-screen	Cheminformatics & Chemogenomics Research Group (CCRG), Indiana University School of Informatics	1	0	Osteoporosis model	0.724±0.036	0.56±0.047	1821	60
177	1117327	alkaline phosphatase stimulation in WNT3A conditioned C2C12 cells-IC50	Cheminformatics & Chemogenomics Research Group (CCRG), Indiana University School of Informatics	1	0	Osteoporosis model	0.616±0.093	0.68±0.089	61	30

178	1117329	insulin secretion from INS-1E cells in the presence of 5 mM glucose-screen	Cheminformatics & Chemogenomics Research Group (CCRG), Indiana University School of Informatics	0	1	Diabetes models	0.852±0.025	0.601±0.05	1791	48
179	1117336	GSK3B-pretreated HCT116 viability from Cell TiterGlo-screen	Cheminformatics & Chemogenomics Research Group (CCRG), Indiana University School of Informatics	1	0	Cytotoxicity, HCT116	0.757±0.017	0.715±0.019	1788	305
180	1117337	VEGF stimulated ADSC/ECFC co-culture CD31-stained tube area decrease-IC50	Cheminformatics & Chemogenomics Research Group (CCRG), Indiana University School of Informatics	1	0	Angiogenesis model	0.676±0.044	0.58±0.048	251	85
181	1117340	VEGF stimulated ADSC/ECFC co-culture CD31-stained tube area decrease-screen	Cheminformatics & Chemogenomics Research Group (CCRG), Indiana University School of Informatics	1	0	Angiogenesis model	0.728±0.02	0.673±0.022	1757	239
182	1117341	GLP1 secretion from NCI-H716 cells-screen	Cheminformatics & Chemogenomics Research Group (CCRG), Indiana University School of Informatics	0	1	Diabetes models	0.793±0.03	0.644±0.043	1788	57
183	1117342	HCT116 viability from Cell TiterGlo-screen	Cheminformatics & Chemogenomics Research Group (CCRG), Indiana University School of Informatics	1	0	Cytotoxicity, HCT116	0.799±0.015	0.748±0.017	1788	340
184	1117343	SW480 viability from Cell TiterGlo-screen	Cheminformatics & Chemogenomics Research Group (CCRG), Indiana University School of Informatics	0	1	Cytotoxicity, SW480	0.732±0.016	0.699±0.016	1788	514
185	1117346	DLD-1 viability from Cell TiterGlo-screen	Cheminformatics & Chemogenomics Research Group (CCRG), Indiana University School of Informatics	1	0	Cytotoxicity, DLD-1	0.763±0.017	0.738±0.018	1788	315

186	1117349	Increased HeLa cells with 2N DNA content-IC50	Cheminformatics & Chemogenomics Research Group (CCRG), Indiana University School of Informatics	1	0	Cell Cycle Modulation (counterscreen)	0.708±0.078	0.77±0.068	119	18
187	1117350	Increased chromatin condensation in HeLa cells-IC50	Cheminformatics & Chemogenomics Research Group (CCRG), Indiana University School of Informatics	1	0	Cell Cycle Modulation (counterscreen)	0.685±0.084	0.581±0.095	120	17
188	1159510	Immunotoxin (HA22) sensitization/mitigation study. Vehicle arm (PBS)	NCGC	1	0	Immunotoxin (HA22)	0.673±0.047	0.76±0.043	357	276
189	1159511	Immunotoxin (SS1P) sensitization/mitigation study - treatment arm	NCGC	1	0	Immunotoxin (SS1P)	0.8±0.048	0.837±0.045	374	319
190	1159512	Immunotoxin (HA22) sensitization/mitigation study - treatment arm (low dose)	NCGC	1	0	Immunotoxin (HA22)	0.705±0.056	0.816±0.049	378	328
191	1159513	Immunotoxin (SS1P) sensitization/mitigation study. Vehicle arm (DMEM)	NCGC	1	0	Immunotoxin (SS1P)	0.699±0.042	0.762±0.039	307	201
192	1159515	qHTS assay to identify small molecule agonists of the NFkB signaling pathway - cell viability counter screen	Tox21	0	1	Cytotoxicity, Agonist, nuclear factor kappa B subunit 1	0.767±0.021	0.633±0.029	5014	132
193	1159517	qHTS assay to identify small molecule agonists of the endoplasmic reticulum stress response signaling pathway - cell viability counter screen	Tox21	0	1	Cytotoxicity, Agonist, endoplasmic reticulum stress response	0.745±0.021	0.664±0.025	4923	158
194	1159519	qHTS assay to identify small molecule agonists of the endoplasmic reticulum stress response signaling pathway: Summary	Tox21	0	1	Agonist, endoplasmic reticulum stress response	0.707±0.041	0.546±0.053	5098	48
195	1159520	qHTS assay to identify small molecule antagonists of the retinoid-related orphan receptor gamma (ROR-gamma) signaling pathway - cell viability counter screen	Tox21	0	1	Cytotoxicity, Antagonist, retinoid-related orphan receptor gamma (ROR-gamma)	0.771±0.016	0.597±0.023	5426	243
196	1159521	qHTS assay to identify small molecule antagonists of the retinoid-related orphan receptor gamma (ROR-gamma) signaling pathway	Tox21	1	0	Antagonist, retinoid-related orphan receptor gamma (ROR-gamma)	0.778±0.011	0.661±0.015	5232	533
197	1159523	qHTS assay to identify small molecule antagonists of the retinoid-related orphan receptor gamma (ROR-gamma) signaling pathway: Summary	Tox21	1	0	Antagonist, retinoid-related orphan receptor gamma (ROR-gamma)	0.798±0.013	0.627±0.02	4140	324
198	1159525	qHTS assay to identify small molecule agonists of the AP-1 signaling pathway - cell viability counter screen	Tox21	0	1	Cytotoxicity, Agonist, AP-1	0.765±0.02	0.663±0.026	5494	156
199	1159528	qHTS assay to identify small molecule agonists of the AP-1 signaling pathway: Summary	Tox21	1	0	Agonist, AP-1	0.744±0.015	0.701±0.016	5299	361
200	1159529	qHTS assay to identify small molecule agonists of the RXR signaling pathway - cell viability counter screen	Tox21	0	1	Cytotoxicity, Agonist, RXR	0.778±0.014	0.711±0.017	5175	318
201	1159551	qHTS assay to identify small molecule antagonists of the retinoid acid receptor (RAR) signaling pathway - cell viability counter screen	Tox21	0	1	Cytotoxicity, Antagonist, retinoid acid receptor (RAR)	0.729±0.028	0.509±0.038	5522	95

202	1159553	qHTS assay to identify small molecule agonists of the retinoic acid receptor (RAR) signaling pathway	Tox21	0	1	Agonist, retinoic acid receptor (RAR)	0.818±0.012	0.722±0.017	4789	298
203	1159555	qHTS assay to identify small molecule antagonists of the retinoic acid receptor (RAR) signaling pathway: Summary	Tox21	1	0	Antagonist, retinoic acid receptor (RAR)	0.768±0.014	0.676±0.017	4219	362
204	1159580	The chemical genetic matrix (CGM) dataset as reported in Wildenhain et al. (2015) Prediction of Synergism from Chemical-Genetic Interactions by Machine Learning. Cell Systems Volume 1, Issue 6, p383-395	chemical genetic matrix	0	4	Synergism from Chemical-Genetic Interactions	0.725±0.014	0.693±0.015	4766	486
205	1224835	qHTS assay to identify small molecule inhibitors of firefly luciferase	Tox21	0	1	Interference, Luciferase	0.848±0.009	0.736±0.013	5461	515
206	1224836	qHTS assay to identify small molecule agonists of the constitutive androstane receptor (CAR) signaling pathway - cell viability counter screen	Tox21	0	1	Cytotoxicity, Agonist, constitutive androstane receptor (CAR)	0.758±0.018	0.586±0.025	5453	202
207	1224838	qHTS assay to identify small molecule antagonists of the constitutive androstane receptor (CAR) signaling pathway	Tox21	1	0	Antagonist, constitutive androstane receptor (CAR)	0.698±0.019	0.566±0.023	5524	253
208	1224839	qHTS assay to identify small molecule agonists of the constitutive androstane receptor (CAR) signaling pathway	Tox21	0	1	Agonist, constitutive androstane receptor (CAR)	0.841±0.009	0.803±0.01	5367	612
209	1224843	qHTS assay to identify small molecule agonists of the thyroid stimulating hormone receptor (TSHR) signaling pathway	Tox21	0	1	Agonist, thyroid stimulating hormone receptor (TSHR)	0.773±0.018	0.649±0.024	5189	180
210	1224844	qHTS assay to identify small molecule agonists of the hypoxia (HIF-1) signaling pathway - cell viability counter screen	Tox21	0	1	Cytotoxicity, Agonist, hypoxia	0.744±0.016	0.613±0.02	5303	313
211	1224845	qHTS assay to identify small molecule agonists of H2AX	Tox21	0	1	Agonist, Histone H2A.x	0.717±0.026	0.551±0.033	5032	121
212	1224847	qHTS assay to identify small molecule agonists of H2AX - cell viability counter screen	Tox21	0	1	Cytotoxicity, Agonist, Histone H2A.x	0.835±0.011	0.745±0.014	5175	363
213	1224867	qHTS RealTime-Glo MT Cell Viability Assay in HepG2 cells - 24 hour	Tox21	0	1	Cytotoxicity, HepG2	0.805±0.017	0.598±0.027	5540	162
214	1224868	qHTS RealTime-Glo MT Cell Viability Assay in HEK293 cells - 32 hour	Tox21	1	0	Cytotoxicity, HEK293	0.826±0.009	0.725±0.013	5130	589
215	1224869	A CellTox Green Cytotoxicity Assay to monitor cytotoxicity in HEK293 cells - 0 hour	Tox21	0	1	Cytotoxicity, HEK293	0.582±0.056	0.433±0.063	5731	39
216	1224870	qHTS RealTime-Glo MT Cell Viability Assay in HepG2 cells - 40 hour	Tox21	0	1	Cytotoxicity, HepG2	0.763±0.02	0.578±0.028	5579	158
217	1224871	A CellTox Green Cytotoxicity Assay to monitor cytotoxicity in HEK293 cells - 40 hour	Tox21	0	1	Cytotoxicity, HEK293	0.73±0.015	0.675±0.016	5298	385
218	1224872	qHTS RealTime-Glo MT Cell Viability Assay in HEK293 cells - 16 hour	Tox21	0	1	Cytotoxicity, HEK293	0.829±0.01	0.724±0.014	5155	430
219	1224873	qHTS RealTime-Glo MT Cell Viability Assay in HepG2 cells - 8 hour	Tox21	0	1	Cytotoxicity, HepG2	0.79±0.021	0.559±0.033	5476	115
220	1224874	qHTS RealTime-Glo MT Cell Viability Assay in HEK293 cells - 40 hour	Tox21	1	0	Cytotoxicity, HEK293	0.818±0.009	0.723±0.012	5146	616
221	1224875	A CellTox Green Cytotoxicity Assay to monitor cytotoxicity in HEK293 cells - 24 hour	Tox21	0	1	Cytotoxicity, HEK293	0.723±0.018	0.623±0.022	5412	239

222	1224876	A CellTox Green Cytotoxicity Assay to monitor cytotoxicity in HepG2 cells - 16 hour	Tox21	0	1	Cytotoxicity, HepG2	0.733±0.018	0.617±0.022	5451	237
223	1224877	qHTS RealTime-Glo MT Cell Viability Assay in HepG2 cells - 32 hour	Tox21	0	1	Cytotoxicity, HepG2	0.777±0.019	0.594±0.027	5555	163
224	1224878	A CellTox Green Cytotoxicity Assay to monitor cytotoxicity in HepG2 cells - 24 hour	Tox21	0	1	Cytotoxicity, HepG2	0.747±0.015	0.676±0.017	5341	342
225	1224879	A CellTox Green Cytotoxicity Assay to monitor cytotoxicity in HepG2 cells - 40 hour	Tox21	0	1	Cytotoxicity, HepG2	0.779±0.012	0.693±0.014	5275	483
226	1224880	qHTS RealTime-Glo MT Cell Viability Assay in HEK293 cells - 0 hour	Tox21	0	1	Cytotoxicity, HEK293	0.824±0.023	0.567±0.042	5565	72
227	1224881	A CellTox Green Cytotoxicity Assay to monitor cytotoxicity in HEK293 cells - 32 hour	Tox21	0	1	Cytotoxicity, HEK293	0.717±0.016	0.65±0.019	5343	309
228	1224883	A CellTox Green Cytotoxicity Assay to monitor cytotoxicity in HepG2 cells - 32 hour	Tox21	0	1	Cytotoxicity, HepG2	0.759±0.013	0.691±0.016	5304	420
229	1224884	A CellTox Green Cytotoxicity Assay to monitor cytotoxicity in HEK293 cells - 8 hour	Tox21	0	1	Cytotoxicity, HEK293	0.603±0.032	0.496±0.036	5513	115
230	1224885	qHTS RealTime-Glo MT Cell Viability Assay in HepG2 cells - 16 hour	Tox21	0	1	Cytotoxicity, HepG2	0.787±0.019	0.593±0.029	5523	150
231	1224887	qHTS RealTime-Glo MT Cell Viability Assay in HEK293 cells - 8 hour	Tox21	0	1	Cytotoxicity, HEK293	0.804±0.012	0.724±0.016	5232	338
232	1224888	A CellTox Green Cytotoxicity Assay to monitor cytotoxicity in HEK293 cells - 16 hour	Tox21	0	1	Cytotoxicity, HEK293	0.696±0.024	0.542±0.03	5479	150
233	1224889	qHTS RealTime-Glo MT Cell Viability Assay in HepG2 cells - 0 hour	Tox21	0	1	Cytotoxicity, HepG2	0.893±0.013	0.71±0.028	5579	106
234	1224890	A CellTox Green Cytotoxicity Assay to monitor cytotoxicity in HepG2 cells - 8 hour	Tox21	0	1	Cytotoxicity, HepG2	0.7±0.024	0.542±0.03	5545	153
235	1224894	qHTS assay to identify small molecule agonists of the hypoxia (HIF-1) signaling pathway: Summary	Tox21	0	1	Agonist, hypoxia	0.622±0.065	0.349±0.076	5490	27
236	1224895	qHTS assay to identify small molecule agonists of the thyroid stimulating hormone receptor (TSHR) signaling pathway: Summary	Tox21	0	1	Agonist, thyroid stimulating hormone receptor (TSHR)	0.77±0.016	0.653±0.021	5427	228
237	1224896	qHTS assay to identify small molecule agonists of H2AX: Summary	Tox21	0	1	Agonist, Histone H2A.x	0.754±0.016	0.589±0.022	5462	269
238	1259241	qHTS assay to identify small molecule antagonists of the estrogen receptor alpha (ER-alpha) signaling pathway using the BG1 cell line in the presence of 0.1 nM 17-beta-estradiol - cell viability counter screen	Tox21	0	1	Cytotoxicity, Antagonist, estrogen receptor alpha (ER-alpha)	0.813±0.019	0.622±0.03	5531	124
239	1259242	qHTS assay to identify small molecule antagonists of the androgen receptor (AR) signaling pathway using the MDA cell line in the presence of 0.5 nM R1881 - cell viability counter screen	Tox21	0	1	Cytotoxicity, Antagonist, androgen receptor (AR)	0.767±0.021	0.586±0.03	5551	141
240	1259243	qHTS assay to identify small molecule antagonists of the androgen receptor (AR) signaling pathway using the MDA cell line in the presence of 0.5 nM R1881	Tox21	0	1	Antagonist, androgen receptor (AR)	0.846±0.008	0.787±0.009	5344	797
241	1259244	qHTS assay to identify small molecule antagonists of the estrogen receptor alpha (ER-alpha) signaling pathway using the BG1 cell line in the presence of 0.1 nM 17-beta-estradiol	Tox21	0	1	Antagonist, estrogen receptor alpha (ER-alpha)	0.763±0.014	0.674±0.018	5416	333

242	1259247	qHTS assay to identify small molecule antagonists of the androgen receptor (AR) signaling pathway using the MDA cell line in the presence of 0.5 nM R1881: Summary	Tox21	0	1	Antagonist, androgen receptor (AR)	0.846±0.008	0.79±0.01	4822	721
243	1259248	qHTS assay to identify small molecule antagonists of the estrogen receptor alpha (ER-alpha) signaling pathway using the BG1 cell line in the presence of 0.1 nM 17-beta-estradiol: Summary	Tox21	0	1	Antagonist, estrogen receptor alpha (ER-alpha)	0.81±0.013	0.683±0.018	5059	299

Table S5: Validation of lignans bioactivity prediction with new models built without lignans. For SVM and Naïve Bayes prediction the lignans were considered active if Pa-pi score was better than 0.15 and Pa-Pi minimum limit was positive. The final prediction takes both methods, if one of the methods points that compound is active it is predicted as active.

CID	Name	AID	Activity Class	Exper. Activity	Pa-Pi SVM	SVM Prediction	Pa-Pi Bayes	Bayes Prediction	Final Prediction	Correct?
10607	podophyllotoxin	493027	1-acylglycerol-3-phosphate O-acyltransferase ABHD5	Inactive	0.48±0.284	Active	0.136±0.843	Inactive	Active	
10607	podophyllotoxin	493035	1-acylglycerol-3-phosphate O-acyltransferase ABHD5	Inactive	0.571±0.307	Active	0.107±0.884	Inactive	Active	
10607	podophyllotoxin	743122	Activate, aryl hydrocarbon receptor (AhR)	Active	0.157±0.175	Inactive	-0.02±0.042	Inactive	Inactive	
10607	podophyllotoxin	743210	Activators, heat shock response	Inactive	-0.082±0.183	Inactive	-0.835±0.19	NA	Inactive	**OK**
10607	podophyllotoxin	743228	Activators, heat shock response	Inactive	-0.984±-0.003	Inactive	-0.866±0.488	Inactive	Inactive	**OK**
10607	podophyllotoxin	884	ADMET, Cytochrome P450 3A4	Active	0.479±0.012	Active	-0.453±0.309	Inactive	Active	**OK**
10607	podophyllotoxin	885	ADMET, Cytochrome P450 3A4	Inactive	-0.391±0.016	Inactive	-0.714±0.187	Inactive	Inactive	**OK**
10607	podophyllotoxin	743040	Agonist, androgen receptor (AR)	Inactive	-0.861±-0.029	Inactive	-0.831±0.385	Inactive	Inactive	**OK**
10607	podophyllotoxin	1159528	Agonist, AP-1	Active	-0.968±-0.01	Inactive	-0.851±0.388	Inactive	Inactive	
10607	podophyllotoxin	1224839	Agonist, constitutive androstane receptor (CAR)	Inactive	-0.649±0.194	Inactive	-0.667±0.268	Inactive	Inactive	**OK**
10607	podophyllotoxin	1159519	Agonist, endoplasmic reticulum stress response	Inactive	-0.589±0.025	Inactive	-0.639±0.641	Inactive	Inactive	**OK**
10607	podophyllotoxin	743079	Agonist, estrogen receptor alpha (ER-alpha)	Inactive	-0.334±0.227	Inactive	-0.708±0.334	Inactive	Inactive	**OK**
10607	podophyllotoxin	743220	Agonist, farnesoid-X-receptor (FXR)	Inactive	-0.573±0.068	Inactive	-0.695±0.217	NA	Inactive	**OK**
10607	podophyllotoxin	743239	Agonist, farnesoid-X-receptor (FXR)	Inactive	-0.952±-0.091	Inactive	-0.784±0.688	NA	Inactive	**OK**
10607	podophyllotoxin	720691	Agonist, glucocorticoid receptor (GR)	Inactive	0.081±0.142	Inactive	-0.732±0.061	Inactive	Inactive	**OK**
10607	podophyllotoxin	720719	Agonist, glucocorticoid receptor (GR)	Inactive	0.123±0.132	Inactive	-0.722±0.412	Inactive	Inactive	**OK**
10607	podophyllotoxin	1224845	Agonist, Histone H2A.x	Inactive	-0.399±0.177	Inactive	-0.81±0.333	Inactive	Inactive	**OK**
10607	podophyllotoxin	1224896	Agonist, Histone H2A.x	Inactive	-0.947±0.011	Inactive	-0.725±0.55	Inactive	Inactive	**OK**
10607	podophyllotoxin	1224894	Agonist, hypoxia	Inactive	-0.743±0.077	Inactive	-0.576±0.235	NA	Inactive	**OK**
10607	podophyllotoxin	651631	Agonist, p53	Inactive	-0.506±0.186	Inactive	0.295±0.705	Inactive	Inactive	**OK**
10607	podophyllotoxin	720552	Agonist, p53	Inactive	-0.579±0.196	Inactive	0.507±0.813	Inactive	Inactive	**OK**
10607	podophyllotoxin	743227	Agonist, peroxisome proliferator-activated receptor delta (PPARd)	Inactive	-0.856±-0.15	Inactive	-0.788±0.202	Inactive	Inactive	**OK**
10607	podophyllotoxin	1159553	Agonist, retinoic acid receptor (RAR)	Inactive	-0.643±0.038	Inactive	-0.673±0.407	Inactive	Inactive	**OK**

10607	podophyllotoxin	743066	Agonist, thyroid receptor (TR)	Inactive	-0.973±-0.008	Inactive	-0.836±0.22	Inactive	Inactive	**OK**
10607	podophyllotoxin	1224843	Agonist, thyroid stimulating hormone receptor (TSHR)	Inactive	-0.974±-0.017	Inactive	-0.9±0.484	Inactive	Inactive	**OK**
10607	podophyllotoxin	1224895	Agonist, thyroid stimulating hormone receptor (TSHR)	Inactive	-0.778±0.037	Inactive	-0.923±0.271	Inactive	Inactive	**OK**
10607	podophyllotoxin	575	Angiogenesis model	Inactive	0.209±0.223	Inactive	0.064±0.821	Inactive	Inactive	**OK**
10607	podophyllotoxin	1117337	Angiogenesis model	Active	0.729±0.153	Active	0.324±0.281	Active	Active	**OK**
10607	podophyllotoxin	1117340	Angiogenesis model	Active	0.298±0.195	Active	-0.509±0.583	Inactive	Active	**OK**
10607	podophyllotoxin	743035	Antagonist, androgen receptor (AR)	Active	-0.853±0.099	Inactive	-0.244±0.494	Inactive	Inactive	
10607	podophyllotoxin	743042	Antagonist, androgen receptor (AR)	Inactive	-0.903±0.071	Inactive	-0.637±0.442	Inactive	Inactive	**OK**
10607	podophyllotoxin	743063	Antagonist, androgen receptor (AR)	Active	-0.982±-0.001	Inactive	-0.621±0.376	Inactive	Inactive	
10607	podophyllotoxin	1259243	Antagonist, androgen receptor (AR)	Inactive	-0.755±0.117	Inactive	-0.302±0.397	Inactive	Inactive	**OK**
10607	podophyllotoxin	1259247	Antagonist, androgen receptor (AR)	Inactive	0.085±0.14	Inactive	0.13±0.065	Inactive	Inactive	**OK**
10607	podophyllotoxin	1224838	Antagonist, constitutive androstane receptor (CAR)	Active	-0.785±0.069	Inactive	-0.891±0.544	Inactive	Inactive	
10607	podophyllotoxin	743069	Antagonist, estrogen receptor alpha (ER-alpha)	Inactive	-0.852±0.025	Inactive	-0.659±0.471	Inactive	Inactive	**OK**
10607	podophyllotoxin	743078	Antagonist, estrogen receptor alpha (ER-alpha)	Inactive	-0.876±0.022	Inactive	-0.558±0.117	Inactive	Inactive	**OK**
10607	podophyllotoxin	743080	Antagonist, estrogen receptor alpha (ER-alpha)	Active	-0.835±0.079	Inactive	-0.577±0.464	Inactive	Inactive	
10607	podophyllotoxin	743091	Antagonist, estrogen receptor alpha (ER-alpha)	Active	-0.805±0.107	Inactive	-0.52±0.448	Inactive	Inactive	
10607	podophyllotoxin	1259244	Antagonist, estrogen receptor alpha (ER-alpha)	Inactive	-0.988±0.001	Inactive	-0.796±0.11	Inactive	Inactive	**OK**
10607	podophyllotoxin	1259248	Antagonist, estrogen receptor alpha (ER-alpha)	Inactive	-0.973±0.007	Inactive	-0.718±0.425	Inactive	Inactive	**OK**
10607	podophyllotoxin	743240	Antagonist, farnesoid-X-receptor (FXR)	Active	-0.777±0.072	Inactive	-0.731±0.561	Inactive	Inactive	
10607	podophyllotoxin	624479	Antagonist, NFkB Signaling Pathway	Inactive	-0.976±0.044	Inactive	-0.73±0.462	Inactive	Inactive	**OK**
10607	podophyllotoxin	743215	Antagonist, peroxisome proliferator-activated receptor delta (PPARd)	Inactive	-0.958±-0.091	Inactive	-0.87±0.611	Inactive	Inactive	**OK**
10607	podophyllotoxin	743191	Antagonist, peroxisome proliferator-activated receptor gamma (PPARg)	Inactive	0.23±0.258	Inactive	-0.384±0.593	Inactive	Inactive	**OK**
10607	podophyllotoxin	1159555	Antagonist, retinoic acid receptor (RAR)	Active	-0.762±0.105	Inactive	-0.742±0.415	Inactive	Inactive	
10607	podophyllotoxin	1159521	Antagonist, retinoid-related orphan receptor gamma (ROR-gamma)	Active	-0.978±0.011	Inactive	-0.657±0.388	Inactive	Inactive	

10607	podophyllotoxin	1159523	Antagonist, retinoid-related orphan receptor gamma (ROR-gamma)	Active	-0.958±0.036	Inactive	-0.01±0.568	Inactive	Inactive	
10607	podophyllotoxin	743065	Antagonist, thyroid receptor (TR)	Active	-0.934±0.033	Inactive	-0.054±0.068	Inactive	Inactive	
10607	podophyllotoxin	743067	Antagonist, thyroid receptor (TR)	Active	-0.141±0.327	Inactive	0.05±0.646	Inactive	Inactive	
10607	podophyllotoxin	743242	Antagonist, vitamin D receptor (VDR)	Active	-0.869±-0.168	Inactive	-0.707±0.696	NA	Inactive	
10607	podophyllotoxin	1834	Anticancer, colon	Active	-0.482±0.321	Inactive	-0.677±0.088	Inactive	Inactive	
10607	podophyllotoxin	771	Anticancer, Colon Tumor	Inactive	-0.886±-0.169	Inactive	-0.915±0.482	Inactive	Inactive	**OK**
10607	podophyllotoxin	250	Anticancer, Leukemia	Inactive	-0.935±0.11	Inactive	-0.897±0.631	Inactive	Inactive	**OK**
10607	podophyllotoxin	258	Anticancer, Leukemia	Inactive	-0.459±0.231	Inactive	-0.827±0.527	Inactive	Inactive	**OK**
10607	podophyllotoxin	328	Anticancer, Leukemia	Active	0.591±0.058	Active	-0.249±0.5	Inactive	Active	**OK**
10607	podophyllotoxin	1053195	Anticancer, Leukemia	Inactive	0.435±0.304	Active	0.863±0.053	Active	Active	
10607	podophyllotoxin	270	Anticancer, Lung Tumor	Inactive	-0.023±0.282	Inactive	-0.833±0.491	Inactive	Inactive	**OK**
10607	podophyllotoxin	192	Anticancer, Melanoma	Inactive	0.069±0.165	Inactive	-0.065±0.583	Inactive	Inactive	**OK**
10607	podophyllotoxin	651558	Anticancer, non-Hodgkin lymphoma	Active	-0.213±0.26	Inactive	-0.485±0.091	Inactive	Inactive	
10607	podophyllotoxin	651568	Anticancer, non-Hodgkin lymphoma	Inactive	-0.555±-0.009	Inactive	0.775±0.252	Active	Active	
10607	podophyllotoxin	292	Anticancer, Sarcoma	Active	0.294±0.242	Active	-0.372±0.547	Inactive	Active	**OK**
10607	podophyllotoxin	686947	Anticancer, Yes1 kinase	Inactive	-0.573±0.128	Inactive	-0.674±0.285	Inactive	Inactive	**OK**
10607	podophyllotoxin	438	Anti-Inflammatory model	Active	-0.51±0.033	Inactive	0.327±0.863	NA	Inactive	
10607	podophyllotoxin	457	Anti-Inflammatory model	Inactive	0.349±0.378	Inactive	0.482±0.873	Inactive	Inactive	**OK**
10607	podophyllotoxin	1267	Anti-Inflammatory model	Inactive	-0.823±0.161	Inactive	0.855±0.061	Active	Active	
10607	podophyllotoxin	1309	Anti-Inflammatory model	Active	0.654±0.22	Active	0.324±0.739	Inactive	Active	**OK**
10607	podophyllotoxin	435020	Anti-Inflammatory model	Active	0.852±0.061	Active	0.921±0.058	Active	Active	**OK**
10607	podophyllotoxin	489033	Anti-Inflammatory model	Inactive	-0.223±0.14	Inactive	0.925±0.026	Active	Active	
10607	podophyllotoxin	489035	Anti-Inflammatory model	Inactive	-0.204±0.239	Inactive	0.924±0.039	Active	Active	
10607	podophyllotoxin	602277	Anti-Inflammatory model	Active	0.589±0.347	Active	-0.019±0.765	Inactive	Active	**OK**
10607	podophyllotoxin	1318	Anti-Inflammatory model (counterscreen)	Inactive	-0.787±0.052	Inactive	0.129±0.114	Inactive	Inactive	**OK**
10607	podophyllotoxin	357	AP1 Signaling Pathway	Inactive	0.031±0.173	Inactive	-0.35±0.302	Inactive	Inactive	**OK**
10607	podophyllotoxin	504459	beta-2 adrenergic receptor	Inactive	0.179±0.485	Inactive	-0.26±0.857	Inactive	Inactive	**OK**
10607	podophyllotoxin	1117349	Cell Cycle Modulation (counterscreen)	Active	0.306±0.111	Active	-0.295±0.305	Inactive	Active	**OK**
10607	podophyllotoxin	1117350	Cell Cycle Modulation (counterscreen)	Active	0.179±0.177	Active	-0.336±0.594	Inactive	Active	**OK**

10607	podophyllotoxin	463189	Chlamydomonas reinhardtii Algae Gravitaxis	Inactive	-0.992±0.019	Inactive	-0.923±0.775	Inactive	Inactive	**OK**
10607	podophyllotoxin	624476	Cytotoxicity	Inactive	-0.753±0.208	Inactive	-0.725±0.561	Inactive	Inactive	**OK**
10607	podophyllotoxin	371	Cytotoxicity, A549	Active	-0.815±0.007	Inactive	-0.914±0.429	Inactive	Inactive	
10607	podophyllotoxin	743086	Cytotoxicity, Activate, aryl hydrocarbon receptor (AhR)	Inactive	0.717±0.195	Active	-0.528±0.587	Inactive	Active	
10607	podophyllotoxin	743209	Cytotoxicity, Activators, heat shock response (counterscreen)	Inactive	-0.967±-0.015	Inactive	-0.911±0.506	Inactive	Inactive	**OK**
10607	podophyllotoxin	743203	Cytotoxicity, Agonist, antioxidant response element (ARE)	Inactive	-0.978±0.001	Inactive	-0.933±0.464	Inactive	Inactive	**OK**
10607	podophyllotoxin	1159525	Cytotoxicity, Agonist, AP-1	Inactive	-0.971±-0.025	Inactive	-0.947±0.507	Inactive	Inactive	**OK**
10607	podophyllotoxin	1224836	Cytotoxicity, Agonist, constitutive androstane receptor (CAR)	Inactive	-0.064±0.295	Inactive	-0.796±0.527	Inactive	Inactive	**OK**
10607	podophyllotoxin	1159517	Cytotoxicity, Agonist, endoplasmic reticulum stress response	Inactive	-0.971±0.01	Inactive	-0.933±0.525	Inactive	Inactive	**OK**
10607	podophyllotoxin	743218	Cytotoxicity, Agonist, farnesoid-X-receptor (FXR)	Inactive	-0.984±-0.006	Inactive	-0.871±0.45	Inactive	Inactive	**OK**
10607	podophyllotoxin	1224847	Cytotoxicity, Agonist, Histone H2A.x	Inactive	-0.978±-0.007	Inactive	-0.789±0.359	Inactive	Inactive	**OK**
10607	podophyllotoxin	1224844	Cytotoxicity, Agonist, hypoxia	Inactive	-0.987±0.003	Inactive	-0.905±0.471	Inactive	Inactive	**OK**
10607	podophyllotoxin	1159515	Cytotoxicity, Agonist, nuclear factor kappa B subunit 1	Inactive	-0.986±0.019	Inactive	-0.937±0.549	Inactive	Inactive	**OK**
10607	podophyllotoxin	651633	Cytotoxicity, Agonist, p53	Inactive	-0.965±-0.001	Inactive	-0.799±0.455	Inactive	Inactive	**OK**
10607	podophyllotoxin	743211	Cytotoxicity, Agonist, peroxisome proliferator-activated receptor delta (PPARd)	Inactive	-0.983±-0.006	Inactive	-0.818±0.391	Inactive	Inactive	**OK**
10607	podophyllotoxin	1159529	Cytotoxicity, Agonist, RXR	Inactive	-0.987±-0.004	Inactive	-0.903±0.204	Inactive	Inactive	**OK**
10607	podophyllotoxin	743224	Cytotoxicity, Agonist, vitamin D receptor (VDR)	Inactive	-0.98±0.012	Inactive	-0.905±0.491	Inactive	Inactive	**OK**
10607	podophyllotoxin	743033	Cytotoxicity, Antagonist, androgen receptor (AR)	Inactive	-0.957±0.003	Inactive	-0.742±0.533	Inactive	Inactive	**OK**
10607	podophyllotoxin	743041	Cytotoxicity, Antagonist, androgen receptor (AR)	Inactive	-0.649±0.123	Inactive	-0.672±0.204	Inactive	Inactive	**OK**
10607	podophyllotoxin	1259242	Cytotoxicity, Antagonist, androgen receptor (AR)	Inactive	-0.795±-0.046	Inactive	-0.864±0.576	Inactive	Inactive	**OK**
10607	podophyllotoxin	743081	Cytotoxicity, Antagonist, estrogen receptor alpha (ER-alpha)	Inactive	-0.643±-0.017	Inactive	-0.684±0.232	Inactive	Inactive	**OK**
10607	podophyllotoxin	1259241	Cytotoxicity, Antagonist, estrogen receptor alpha (ER-alpha)	Inactive	-0.957±-0.016	Inactive	-0.786±0.476	Inactive	Inactive	**OK**
10607	podophyllotoxin	743221	Cytotoxicity, Antagonist, farnesoid-X-receptor (FXR)	Inactive	-0.972±-0.007	Inactive	-0.866±0.567	Inactive	Inactive	**OK**

10607	podophyllotoxin	720693	Cytotoxicity, Antagonist, glucocorticoid receptor (GR)	Inactive	-0.892±-0.034	Inactive	-0.845±0.527	Inactive	Inactive	**OK**
10607	podophyllotoxin	743194	Cytotoxicity, Antagonist, peroxisome proliferator-activated receptor gamma (PPARg)	Inactive	-0.989±0.001	Inactive	-0.933±0.495	Inactive	Inactive	**OK**
10607	podophyllotoxin	1159551	Cytotoxicity, Antagonist, retinoid acid receptor (RAR)	Inactive	-0.948±-0.02	Inactive	-0.812±0.634	Inactive	Inactive	**OK**
10607	podophyllotoxin	1159520	Cytotoxicity, Antagonist, retinoid-related orphan receptor gamma (ROR-gamma)	Inactive	-0.985±0.008	Inactive	-0.898±0.493	Inactive	Inactive	**OK**
10607	podophyllotoxin	743064	Cytotoxicity, Antagonist, thyroid receptor (TR)	Active	-0.929±0.048	Inactive	0.109±0.59	Inactive	Inactive	
10607	podophyllotoxin	743225	Cytotoxicity, Antagonist, vitamin D receptor (VDR)	Inactive	-0.986±-0.004	Inactive	-0.926±0.534	Inactive	Inactive	**OK**
10607	podophyllotoxin	1117346	Cytotoxicity, DLD-1	Active	-0.691±0.136	Inactive	-0.378±0.504	Inactive	Inactive	
10607	podophyllotoxin	1117336	Cytotoxicity, HCT116	Active	-0.727±0.191	Inactive	-0.446±0.203	Inactive	Inactive	
10607	podophyllotoxin	1117342	Cytotoxicity, HCT116	Active	-0.723±0.167	Inactive	-0.271±0.125	Inactive	Inactive	
10607	podophyllotoxin	1224868	Cytotoxicity, HEK293	Active	-0.885±0.014	Inactive	-0.676±0.346	Inactive	Inactive	
10607	podophyllotoxin	1224869	Cytotoxicity, HEK293	Inactive	-0.489±0.03	Inactive	-0.69±0.217	NA	Inactive	**OK**
10607	podophyllotoxin	1224871	Cytotoxicity, HEK293	Inactive	-0.965±0.014	Inactive	-0.824±0.401	Inactive	Inactive	**OK**
10607	podophyllotoxin	1224872	Cytotoxicity, HEK293	Inactive	-0.979±0.006	Inactive	-0.798±0.35	Inactive	Inactive	**OK**
10607	podophyllotoxin	1224874	Cytotoxicity, HEK293	Active	-0.833±0.052	Inactive	-0.678±0.328	Inactive	Inactive	
10607	podophyllotoxin	1224875	Cytotoxicity, HEK293	Inactive	-0.894±-0.017	Inactive	-0.926±0.333	Inactive	Inactive	**OK**
10607	podophyllotoxin	1224880	Cytotoxicity, HEK293	Inactive	-0.555±-0.012	Inactive	-0.784±0.543	Inactive	Inactive	**OK**
10607	podophyllotoxin	1224881	Cytotoxicity, HEK293	Inactive	-0.966±0.006	Inactive	-0.882±0.468	Inactive	Inactive	**OK**
10607	podophyllotoxin	1224884	Cytotoxicity, HEK293	Inactive	-0.813±0.074	Inactive	-0.857±0.611	NA	Inactive	**OK**
10607	podophyllotoxin	1224887	Cytotoxicity, HEK293	Inactive	-0.961±-0.004	Inactive	-0.838±0.373	Inactive	Inactive	**OK**
10607	podophyllotoxin	1224888	Cytotoxicity, HEK293	Inactive	-0.949±-0.053	Inactive	-0.917±0.645	Inactive	Inactive	**OK**
10607	podophyllotoxin	1224867	Cytotoxicity, HepG2	Inactive	-0.975±-0.045	Inactive	-0.911±0.344	Inactive	Inactive	**OK**
10607	podophyllotoxin	1224870	Cytotoxicity, HepG2	Inactive	-0.977±-0.012	Inactive	-0.905±0.318	Inactive	Inactive	**OK**
10607	podophyllotoxin	1224873	Cytotoxicity, HepG2	Inactive	-0.985±-0.029	Inactive	-0.92±0.594	Inactive	Inactive	**OK**
10607	podophyllotoxin	1224876	Cytotoxicity, HepG2	Inactive	-0.785±0.034	Inactive	-0.806±0.483	Inactive	Inactive	**OK**
10607	podophyllotoxin	1224877	Cytotoxicity, HepG2	Inactive	-0.982±-0.026	Inactive	-0.908±0.564	Inactive	Inactive	**OK**
10607	podophyllotoxin	1224878	Cytotoxicity, HepG2	Inactive	-0.85±0.03	Inactive	-0.733±0.412	Inactive	Inactive	**OK**

10607	podophyllotoxin	1224879	Cytotoxicity, HepG2	Inactive	-0.917±0.007	Inactive	-0.621±0.388	Inactive	Inactive	**OK**
10607	podophyllotoxin	1224883	Cytotoxicity, HepG2	Inactive	-0.818±0.041	Inactive	-0.719±0.127	Inactive	Inactive	**OK**
10607	podophyllotoxin	1224885	Cytotoxicity, HepG2	Inactive	-0.986±-0.015	Inactive	-0.919±0.551	Inactive	Inactive	**OK**
10607	podophyllotoxin	1224889	Cytotoxicity, HepG2	Inactive	-0.711±-0.074	Inactive	-0.791±0.416	Inactive	Inactive	**OK**
10607	podophyllotoxin	1224890	Cytotoxicity, HepG2	Inactive	-0.747±0.025	Inactive	-0.846±0.592	Inactive	Inactive	**OK**
10607	podophyllotoxin	1905	Cytotoxicity, IEC-6 intestinal epithelial	Active	0.411±0.115	Active	-0.232±0.153	Inactive	Active	**OK**
10607	podophyllotoxin	1907	Cytotoxicity, IEC-6 intestinal epithelial	Active	0.354±0.258	Active	-0.773±0.075	Inactive	Active	**OK**
10607	podophyllotoxin	743084	Cytotoxicity, inhibitors, aromatase	Inactive	-0.873±0.014	Inactive	-0.477±0.481	Inactive	Inactive	**OK**
10607	podophyllotoxin	743012	Cytotoxicity, isogenic chicken DT40 cell line	Active	0.246±0.109	Active	0.315±0.042	Active	Active	**OK**
10607	podophyllotoxin	743014	Cytotoxicity, isogenic chicken DT40 cell line	Active	-0.482±0.285	Inactive	0.311±0.06	Active	Active	**OK**
10607	podophyllotoxin	743015	Cytotoxicity, isogenic chicken DT40 cell line	Active	0.196±0.116	Active	0.346±0.027	Active	Active	**OK**
10607	podophyllotoxin	364	Cytotoxicity, Jurkat	Active	-0.098±0.159	Inactive	-0.717±0.465	Inactive	Inactive	
10607	podophyllotoxin	720634	Cytotoxicity, Mitochondria, membrane potential	Inactive	-0.89±-0.047	Inactive	-0.825±0.142	Inactive	Inactive	**OK**
10607	podophyllotoxin	687029	Cytotoxicity, RMG-1 (PAX8)	Inactive	-0.598±0.118	Inactive	-0.238±0.384	Inactive	Inactive	**OK**
10607	podophyllotoxin	743021	Cytotoxicity, RMG-1 (PAX8)	Inactive	-0.941±0.049	Inactive	-0.022±0.034	Inactive	Inactive	**OK**
10607	podophyllotoxin	624031	Cytotoxicity, S16	Inactive	-0.883±-0.06	Inactive	-0.766±0.652	Inactive	Inactive	**OK**
10607	podophyllotoxin	1117343	Cytotoxicity, SW480	Inactive	-0.83±0.132	Inactive	-0.078±0.545	Inactive	Inactive	**OK**
10607	podophyllotoxin	651712	Cytotoxicity, TMD8	Active	-0.409±0.259	Inactive	-0.691±0.541	Inactive	Inactive	
10607	podophyllotoxin	521220	Citotoxidade, mouse neural precursor cells	Active	0.476±0.324	Active	-0.436±0.787	Inactive	Active	**OK**
10607	podophyllotoxin	1990	Counterscreen, Luciferase	Active	-0.014±0.385	Inactive	0.891±0.027	Active	Active	**OK**
10607	podophyllotoxin	624030	Counterscreen, Luciferase	Inactive	-0.812±-0.067	Inactive	-0.433±0.647	Inactive	Inactive	**OK**
10607	podophyllotoxin	1117329	Diabetes models	Inactive	-0.043±0.168	Inactive	-0.75±0.825	Inactive	Inactive	**OK**
10607	podophyllotoxin	1117341	Diabetes models	Inactive	-0.893±-0.057	Inactive	-0.635±0.745	Inactive	Inactive	**OK**
10607	podophyllotoxin	642	dopamine D1 receptor	Active	-0.34±0.186	Inactive	-0.667±0.036	Inactive	Inactive	
10607	podophyllotoxin	647	dopamine D1 receptor	Active	-0.112±0.206	Inactive	-0.771±0.2	Inactive	Inactive	
10607	podophyllotoxin	588506	Fungus, Candida albicans	Inactive	0.15±0.952	NA	-0.551±0.775	NA	Inactive	**OK**
10607	podophyllotoxin	651632	Genotoxicity, ATAD5	Active	-0.277±0.159	Inactive	-0.572±0.538	Inactive	Inactive	
10607	podophyllotoxin	651634	Genotoxicity, ATAD5	Inactive	-0.225±0.189	Inactive	-0.689±0.534	Inactive	Inactive	**OK**

10607	podophyllotoxin	720516	Genotoxicity, ATAD5	Active	-0.669±-0.013	Inactive	-0.535±0.514	Inactive	Inactive	
10607	podophyllotoxin	450	glucocorticoid receptor (GR)	Inactive	-0.87±-0.174	Inactive	-0.47±-0.039	Inactive	Inactive	**OK**
10607	podophyllotoxin	493083	Hsf1 protein	Inactive	0.306±0.396	Inactive	0.626±0.212	Active	Active	
10607	podophyllotoxin	483	Huntington disease protein	Inactive	-0.699±0.276	Inactive	0.705±0.9	NA	Inactive	**OK**
10607	podophyllotoxin	1159510	Immunotoxin (HA22)	Active	-0.791±0.191	Inactive	-0.615±0.066	Inactive	Inactive	
10607	podophyllotoxin	1159512	Immunotoxin (HA22)	Active	-0.837±0.128	Inactive	-0.598±0.228	Inactive	Inactive	
10607	podophyllotoxin	1159511	Immunotoxin (SS1P)	Active	-0.881±0.116	Inactive	-0.545±0.259	Inactive	Inactive	
10607	podophyllotoxin	1159513	Immunotoxin (SS1P)	Active	-0.757±0.176	Inactive	-0.652±0.282	Inactive	Inactive	
10607	podophyllotoxin	743083	Inhibitors, Aromatase	Inactive	-0.746±0.04	Inactive	-0.537±0.052	Inactive	Inactive	**OK**
10607	podophyllotoxin	743139	Inhibitors, Aromatase	Inactive	-0.825±0.048	Inactive	0.32±0.712	Inactive	Inactive	**OK**
10607	podophyllotoxin	720678	Interference, Auto Fluorescence in HEK293	Inactive	0.799±0.388	Active	-0.705±0.214	NA	Active	
10607	podophyllotoxin	720681	Interference, Auto Fluorescence in HEK293	Inactive	0.624±0.293	Active	-0.7±0.158	Inactive	Active	
10607	podophyllotoxin	720685	Interference, Auto Fluorescence in HepG2	Inactive	0.378±0.358	Active	-0.734±0.188	Inactive	Active	
10607	podophyllotoxin	720687	Interference, Auto Fluorescence in HepG2	Inactive	0.586±0.34	Active	-0.72±0.112	Inactive	Active	
10607	podophyllotoxin	1224835	Interference, Luciferase	Inactive	-0.894±-0.028	Inactive	-0.459±0.055	Inactive	Inactive	**OK**
10607	podophyllotoxin	720635	Mitochondria, disruptor membrane potential	Active	0.103±0.102	Inactive	0.198±0.386	Active	Active	**OK**
10607	podophyllotoxin	720637	Mitochondria, disruptor membrane potential	Active	-0.176±0.161	Inactive	0.225±0.048	Active	Active	**OK**
10607	podophyllotoxin	1332	Mycobacterium tuberculosis	Inactive	-0.962±0.011	Inactive	-0.689±0.592	Inactive	Inactive	**OK**
10607	podophyllotoxin	489041	NF-kB induction (counterscreen)	Active	-0.259±0.273	Inactive	-0.653±0.068	Inactive	Inactive	
10607	podophyllotoxin	599	Nuclear receptor ROR-alpha (counterscreen)	Active	-0.04±0.208	Inactive	0.068±0.197	Inactive	Inactive	
10607	podophyllotoxin	652134	nuclear receptor subfamily 0 group B member 1	Active	-0.419±0.169	Inactive	-0.369±0.421	Inactive	Inactive	
10607	podophyllotoxin	687017	nuclear receptor subfamily 0 group B member 1	Active	-0.224±0.454	Inactive	0.599±0.182	Active	Active	**OK**
10607	podophyllotoxin	1117325	Osteoporosis model	Active	0.012±0.36	Inactive	0.308±0.246	Inactive	Inactive	
10607	podophyllotoxin	1117326	Osteoporosis model	Active	0.465±0.241	Active	-0.496±0.767	Inactive	Active	**OK**
10607	podophyllotoxin	1117327	Osteoporosis model	Active	0.837±0.243	Active	0.835±0.21	Active	Active	**OK**
10607	podophyllotoxin	608	peptidyl-prolyl cis-trans isomerase FKBP1A	Inactive	-0.512±-0.006	Inactive	-0.92±0.41	Inactive	Inactive	**OK**

10607	podophyllotoxin	624032	peripheral myelin protein 22	Active	-0.229±0.324	Inactive	-0.548±0.55	Inactive	Inactive	
10607	podophyllotoxin	624044	peripheral myelin protein 22	Active	0.66±0.252	Active	-0.026±0.792	NA	Active	**OK**
10607	podophyllotoxin	743244	Plasmodium falciparum	Inactive	-0.908±0.003	Inactive	-0.683±0.516	Inactive	Inactive	**OK**
10607	podophyllotoxin	636	Post-Golgi Transport	Inactive	-0.211±0.056	Inactive	0.529±0.9	NA	Inactive	**OK**
10607	podophyllotoxin	624	potassium voltage-gated channel subfamily J member 3	Inactive	-0.96±-0.038	Inactive	0.454±0.89	Inactive	Inactive	**OK**
10607	podophyllotoxin	504592	Prion Protein 5' UTR mRNA	Active	0.475±0.172	Active	0.777±0.045	Active	Active	**OK**
10607	podophyllotoxin	446	Stat Signaling Pathway	Inactive	-0.283±0.086	Inactive	-0.346±0.909	NA	Inactive	**OK**
10607	podophyllotoxin	600	steroidogenic factor 1	Active	-0.732±0.228	Inactive	0.117±0.389	Inactive	Inactive	
10607	podophyllotoxin	652136	steroidogenic factor 1 (counterscreen)	Inactive	-0.84±0.085	Inactive	-0.247±0.056	Inactive	Inactive	**OK**
10607	podophyllotoxin	1159580	Synergism from Chemical-Genetic Interactions	Inactive	-0.497±0.158	Inactive	-0.602±0.416	Inactive	Inactive	**OK**
10607	podophyllotoxin	651629	Triacylglycerol inhbitors	Active	-0.287±0.235	Inactive	-0.057±0.246	Inactive	Inactive	
10607	podophyllotoxin	651630	Triacylglycerol inhbitors	Inactive	-0.441±0.291	Inactive	0.311±0.73	Inactive	Inactive	**OK**
10607	podophyllotoxin	504865	USP1 protein	Inactive	-0.785±-0.056	Inactive	-0.404±0.194	Inactive	Inactive	**OK**
10607	podophyllotoxin	1117304	Virus, Ebola	Active	0.156±0.114	Active	-0.4±0.43	Inactive	Active	**OK**
10607	podophyllotoxin	1117305	Virus, Ebola	Active	0.105±0.149	Inactive	-0.432±0.414	Inactive	Inactive	
10607	podophyllotoxin	1117312	Virus, Ebola	Active	-0.219±0.204	Inactive	-0.427±0.417	Inactive	Inactive	
122797	4'-Demethyl-epipodophyllotoxin	743122	Activate, aryl hydrocarbon receptor (AhR)	Active	0.134±0.171	Inactive	-0.306±0.379	Inactive	Inactive	
122797	4'-Demethyl-epipodophyllotoxin	743040	Agonist, androgen receptor (AR)	Inactive	-0.811±0.007	Inactive	-0.799±0.388	Inactive	Inactive	**OK**
122797	4'-Demethyl-epipodophyllotoxin	743079	Agonist, estrogen receptor alpha (ER-alpha)	Inactive	0.501±0.12	Active	-0.03±0.554	Inactive	Active	
122797	4'-Demethyl-epipodophyllotoxin	720691	Agonist, glucocorticoid receptor (GR)	Inactive	0.081±0.137	Inactive	-0.708±0.407	Inactive	Inactive	**OK**
122797	4'-Demethyl-epipodophyllotoxin	720719	Agonist, glucocorticoid receptor (GR)	Inactive	0.137±0.134	Inactive	-0.703±0.412	Inactive	Inactive	**OK**
122797	4'-Demethyl-epipodophyllotoxin	651631	Agonist, p53	Active	0.499±0.192	Active	0.464±0.164	Active	Active	**OK**
122797	4'-Demethyl-epipodophyllotoxin	720552	Agonist, p53	Active	0.445±0.251	Active	0.586±0.159	Active	Active	**OK**
122797	4'-Demethyl-epipodophyllotoxin	743035	Antagonist, androgen receptor (AR)	Active	-0.9±0.05	Inactive	-0.608±0.385	Inactive	Inactive	
122797	4'-Demethyl-epipodophyllotoxin	743042	Antagonist, androgen receptor (AR)	Inactive	-0.909±0.068	Inactive	-0.708±0.436	Inactive	Inactive	**OK**
122797	4'-Demethyl-epipodophyllotoxin	743063	Antagonist, androgen receptor (AR)	Active	-0.752±0.104	Inactive	-0.69±0.366	Inactive	Inactive	
122797	4'-Demethyl-epipodophyllotoxin	743069	Antagonist, estrogen receptor alpha (ER-alpha)	Inactive	0.462±0.198	Active	-0.528±0.481	Inactive	Active	
122797	4'-Demethyl-epipodophyllotoxin	743078	Antagonist, estrogen receptor alpha (ER-alpha)	Inactive	0.504±0.201	Active	-0.33±0.501	Inactive	Active	

122797	4'-Demethyl-epipodophyllotoxin	743065	Antagonist, thyroid receptor (TR)	Active	-0.078±0.196	Inactive	-0.411±0.069	Inactive	Inactive	
122797	4'-Demethyl-epipodophyllotoxin	743086	Cytotoxicity, Activate, aryl hydrocarbon receptor (AhR)	Active	0.75±0.161	Active	-0.454±0.595	Inactive	Active	**OK**
122797	4'-Demethyl-epipodophyllotoxin	651633	Cytotoxicity, Agonist, p53	Inactive	-0.961±0.001	Inactive	-0.773±0.457	Inactive	Inactive	**OK**
122797	4'-Demethyl-epipodophyllotoxin	743074	Cytotoxicity, Antagonist, estrogen receptor alpha (ER-alpha)	Inactive	-0.966±-0.014	Inactive	-0.85±0.617	Inactive	Inactive	**OK**
122797	4'-Demethyl-epipodophyllotoxin	720693	Cytotoxicity, Antagonist, glucocorticoid receptor (GR)	Inactive	-0.915±-0.033	Inactive	-0.821±0.528	Inactive	Inactive	**OK**
122797	4'-Demethyl-epipodophyllotoxin	743064	Cytotoxicity, Antagonist, thyroid receptor (TR)	Active	0.024±0.198	Inactive	0.049±0.562	Inactive	Inactive	
122797	4'-Demethyl-epipodophyllotoxin	743084	Cytotoxicity, inhibitors, aromatase	Inactive	-0.567±0.12	Inactive	-0.3±0.513	Inactive	Inactive	**OK**
122797	4'-Demethyl-epipodophyllotoxin	743012	Cytotoxicity, isogenic chicken DT40 cell line	Active	0.224±0.117	Active	-0.271±0.432	Inactive	Active	**OK**
122797	4'-Demethyl-epipodophyllotoxin	743014	Cytotoxicity, isogenic chicken DT40 cell line	Active	-0.2±0.224	Inactive	-0.226±0.444	Inactive	Inactive	
122797	4'-Demethyl-epipodophyllotoxin	743015	Cytotoxicity, isogenic chicken DT40 cell line	Active	0.253±0.109	Active	0.008±0.554	Inactive	Active	**OK**
122797	4'-Demethyl-epipodophyllotoxin	720634	Cytotoxicity, Mitochondria, membrane potential	Inactive	-0.814±-0.032	Inactive	-0.835±0.428	Inactive	Inactive	**OK**
122797	4'-Demethyl-epipodophyllotoxin	651632	Genotoxicity, ATAD5	Active	0.229±0.257	Inactive	-0.539±0.133	Inactive	Inactive	
122797	4'-Demethyl-epipodophyllotoxin	651634	Genotoxicity, ATAD5	Active	0.53±0.175	Active	-0.546±0.539	Inactive	Active	**OK**
122797	4'-Demethyl-epipodophyllotoxin	720516	Genotoxicity, ATAD5	Active	0.337±0.257	Active	-0.441±0.522	Inactive	Active	**OK**
122797	4'-Demethyl-epipodophyllotoxin	743083	Inhibitors, Aromatase	Inactive	-0.533±0.205	Inactive	-0.689±0.374	Inactive	Inactive	**OK**
122797	4'-Demethyl-epipodophyllotoxin	743139	Inhibitors, Aromatase	Inactive	-0.907±-0.018	Inactive	-0.438±0.479	Inactive	Inactive	**OK**
122797	4'-Demethyl-epipodophyllotoxin	720678	Interference, Auto Fluorescence in HEK293	Inactive	0.699±0.401	Active	-0.692±0.218	NA	Active	
122797	4'-Demethyl-epipodophyllotoxin	720681	Interference, Auto Fluorescence in HEK293	Inactive	0.561±0.346	Active	-0.693±0.16	Inactive	Active	
122797	4'-Demethyl-epipodophyllotoxin	720685	Interference, Auto Fluorescence in HepG2	Inactive	0.184±0.325	Inactive	-0.724±0.191	Inactive	Inactive	**OK**
122797	4'-Demethyl-epipodophyllotoxin	720687	Interference, Auto Fluorescence in HepG2	Inactive	0.528±0.344	Active	-0.713±0.114	Inactive	Active	
122797	4'-Demethyl-epipodophyllotoxin	720635	Mitochondria, disruptor membrane potential	Active	0.078±0.115	Inactive	0.054±0.049	Inactive	Inactive	
122797	4'-Demethyl-epipodophyllotoxin	720637	Mitochondria, disruptor membrane potential	Active	-0.023±0.13	Inactive	0.152±0.404	Active	Active	**OK**
122797	4'-Demethyl-epipodophyllotoxin	743244	Plasmodium falciparum	Inactive	-0.873±-0.009	Inactive	-0.715±0.23	Inactive	Inactive	**OK**
36462	Etoposideo	743472	Cytotoxicity, A549	Inactive	-0.384±0.367	Inactive	-0.47±0.319	Inactive	Inactive	**OK**

36462	Etoposideo	504861	Cytotoxicity, HEK293	Active	-0.697±0.152	Inactive	-0.356±0.532	Inactive	Inactive	
36462	Etoposideo	743471	Cytotoxicity, HepG2	Inactive	-0.035±0.749	Inactive	0.867±0.08	Active	Active	
36462	Etoposideo	521220	Citotoxidade, mouse neural precursor cells	Inactive	0.674±0.273	Active	-0.657±0.783	Inactive	Active	
36462	Etoposideo	537733	Fungus, Candida albicans	Active	0.164±0.505	Inactive	0.64±0.045	Active	Active	**OK**
36462	Etoposideo	588794	Herpes simplex virus Virion Protein 16 (counterscreen)	Inactive	0.439±0.234	Active	0.857±0.074	Active	Active	
36462	Etoposideo	588824	Herpes simplex virus Virion Protein 16 (counterscreen)	Inactive	0.402±0.289	Active	0.855±0.055	Active	Active	
36462	Etoposideo	651615	Herpes simplex virus Virion Protein 16 (counterscreen)	Inactive	0.95±0.029	Active	0.846±0.072	Active	Active	
36462	Etoposideo	1332	Mycobacterium tuberculosis	Inactive	-0.989±0.015	Inactive	-0.852±0.585	Inactive	Inactive	**OK**
36462	Etoposideo	651613	nuclear receptor subfamily 5 group A member 2	Active	0.858±0.255	Active	0.787±0.012	Active	Active	**OK**
36462	Etoposideo	588820	Steroid Receptor Coactivator 1	Active	0.442±0.241	Active	0.8±0.088	Active	Active	**OK**
36462	Etoposideo	602235	Steroid Receptor Coactivator 1	Active	-0.952±0.127	Inactive	-0.947±0.637	Inactive	Inactive	
36462	Etoposideo	602168	Steroid Receptor Coactivator 1 (counterscreen)	Inactive	0.542±0.26	Active	0.731±0.155	Active	Active	
36462	Etoposideo	588792	Steroid Receptor Coactivator 3	Active	0.579±0.161	Active	0.803±0.06	Active	Active	**OK**
36462	Etoposideo	602166	Steroid Receptor Coactivator 3	Active	0.114±0.442	Inactive	0.583±0.335	Active	Active	**OK**
36462	Etoposideo	602234	Steroid Receptor Coactivator 3 (counterscreen)	Active	-0.882±0.144	Inactive	-0.902±0.154	NA	Inactive	
36462	Etoposideo	651611	Steroidogenic acute regulatory protein (StAR) promoter (counterscreen)	Active	0.975±0.012	Active	0.844±0.064	Active	Active	**OK**
36462	Etoposideo	504882	Virus, Epstein-Barr	Active	-0.562±0.189	Inactive	-0.802±0.474	Inactive	Inactive	
36462	Etoposideo	588343	Virus, Epstein-Barr	Active	-0.93±0.063	Inactive	-0.881±0.302	Inactive	Inactive	
36462	Etoposideo	588398	Virus, Epstein-Barr	Active	-0.966±0.084	Inactive	0.788±0.175	Active	Active	**OK**
100492	Diphyllin	624002	Anticancer, glioblastoma	Active	0.523±0.296	Active	0.211±-0.088	Active	Active	**OK**
100492	Diphyllin	1013	Anti-Inflammatory model	Active	0.252±0.278	Inactive	0.819±0.159	Active	Active	**OK**
100492	Diphyllin	1249	Anti-Inflammatory model	Inactive	-0.244±0.131	Inactive	0.654±0.939	NA	Inactive	**OK**
100492	Diphyllin	464	Cytotoxicity, Jurkat	Inactive	0.237±0.225	Active	-0.478±0.151	Inactive	Active	
100492	Diphyllin	2010	Cytotoxicity, NIH3T3	Inactive	-0.428±0.254	Inactive	0.79±0.119	Active	Active	
100492	Diphyllin	2327	Cytotoxicity, NIH3T3	Active	-0.791±0.201	Inactive	0.684±0.127	Active	Active	**OK**
100492	Diphyllin	1650	Cytotoxicity, Vero Cells	Inactive	-0.731±0.111	Inactive	0.847±0.039	Active	Active	
100492	Diphyllin	2423	Fungus, Candida albicans	Active	0.003±0.289	Inactive	0.713±0.024	Active	Active	**OK**

100492	Diphyllin	2467	Fungus, Candida albicans	Active	-0.024±0.267	Inactive	-0.357±0.27	Inactive	Inactive	
100492	Diphyllin	588350	GAA30 promoter (counterscreen)	Inactive	-0.131±0.196	Inactive	0.694±0.008	Active	Active	
100492	Diphyllin	434954	glycogen synthase kinase-3 alpha	Active	-0.693±0.116	Inactive	0.11±0.643	Inactive	Inactive	
100492	Diphyllin	624379	Herpes simplex virus Virion Protein 16 (counterscreen)	Inactive	-0.011±0.212	Inactive	0.058±0.241	Inactive	Inactive	**OK**
100492	Diphyllin	624395	Herpes simplex virus Virion Protein 16 (counterscreen)	Active	0.495±0.381	Active	0.831±0.041	Active	Active	**OK**
100492	Diphyllin	652134	nuclear receptor subfamily 0 group B member 1	Active	-0.076±0.183	Inactive	0.498±-0.07	Active	Active	**OK**
100492	Diphyllin	687017	nuclear receptor subfamily 0 group B member 1	Inactive	-0.125±0.446	Inactive	0.599±0.177	Active	Active	
100492	Diphyllin	624378	photoreceptor-specific nuclear receptor	Active	0.166±0.281	Inactive	0±0.203	Inactive	Inactive	
100492	Diphyllin	624394	photoreceptor-specific nuclear receptor	Active	0.056±0.563	NA	-0.51±0.451	Inactive	Inactive	
100492	Diphyllin	504848	Plasmodium falciparum	Active	0.086±0.244	Inactive	-0.727±0.177	Inactive	Inactive	
100492	Diphyllin	504850	Plasmodium falciparum	Active	-0.243±0.242	Inactive	-0.646±0.228	Inactive	Inactive	
100492	Diphyllin	652136	steroidogenic factor 1 (counterscreen)	Inactive	-0.732±0.117	Inactive	-0.282±0.425	Inactive	Inactive	**OK**
92122	beta-Peltatin	264	Anticancer, Human Lung LX-1 Xenograft	Active	0.43±0.739	NA	-0.063±0.712	Inactive	Inactive	
92122	beta-Peltatin	296	Anticancer, Human Mammary Carcinoma	Inactive	-0.878±0.306	Inactive	-0.843±0.589	Inactive	Inactive	**OK**
92122	beta-Peltatin	111	Anticancer, Leukemia	Active	0.606±0.054	Active	0.488±0.806	Inactive	Active	**OK**
92122	beta-Peltatin	117	Anticancer, Leukemia	Active	0.358±0.137	Active	0.015±0.735	Inactive	Active	**OK**
92122	beta-Peltatin	258	Anticancer, Leukemia	Inactive	-0.756±0.155	Inactive	-0.905±0.517	Inactive	Inactive	**OK**
92122	beta-Peltatin	328	Anticancer, Leukemia	Active	0.486±0.087	Active	-0.41±0.095	Inactive	Active	**OK**
92122	beta-Peltatin	192	Anticancer, Melanoma	Inactive	-0.511±0.059	Inactive	-0.267±0.557	Inactive	Inactive	**OK**
92122	beta-Peltatin	127	Anticancer, Renal tumor	Active	0.531±0.064	Active	0.295±0.731	Inactive	Active	**OK**
92122	beta-Peltatin	292	Anticancer, Sarcoma	Active	0.352±0.252	Active	-0.368±0.547	Inactive	Active	**OK**
92122	beta-Peltatin	608	peptidyl-prolyl cis-trans isomerase FKBP1A	Inactive	-0.452±-0.012	Inactive	-0.923±0.413	Inactive	Inactive	**OK**
92122	beta-Peltatin	1159580	Synergism from Chemical-Genetic Interactions	Inactive	-0.729±0.072	Inactive	-0.446±0.434	Inactive	Inactive	**OK**
92129	alpha-Peltatin	264	Anticancer, Human Lung LX-1 Xenograft	Active	0.516±0.727	NA	-0.209±0.593	Inactive	Inactive	
92129	alpha-Peltatin	296	Anticancer, Human Mammary Carcinoma	Inactive	-0.506±0.496	Inactive	-0.763±0.591	Inactive	Inactive	**OK**
92129	alpha-Peltatin	111	Anticancer, Leukemia	Active	0.691±0.053	Active	0.609±0.092	Active	Active	**OK**

92129	alpha-Peltatin	117	Anticancer, Leukemia	Active	0.481±0.097	Active	0.381±0.752	Inactive	Active	**OK**
92129	alpha-Peltatin	258	Anticancer, Leukemia	Inactive	-0.834±0.142	Inactive	-0.945±0.517	Inactive	Inactive	**OK**
92129	alpha-Peltatin	190	Anticancer, Lymphoma	Inactive	0.095±0.166	Inactive	-0.558±0.427	Inactive	Inactive	**OK**
92129	alpha-Peltatin	192	Anticancer, Melanoma	Inactive	-0.587±0.069	Inactive	-0.087±0.579	Inactive	Inactive	**OK**
92129	alpha-Peltatin	127	Anticancer, Renal tumor	Active	0.562±0.019	Active	0.495±0.07	Active	Active	**OK**
345501	Deoxypodophyllotoxin	111	Anticancer, Leukemia	Active	0.435±0.115	Active	-0.166±0.275	Inactive	Active	**OK**
345501	Deoxypodophyllotoxin	117	Anticancer, Leukemia	Active	-0.18±0.255	Inactive	-0.328±0.718	Inactive	Inactive	
345501	Deoxypodophyllotoxin	328	Anticancer, Leukemia	Active	0.498±0.067	Active	-0.621±0.437	Inactive	Active	**OK**
345501	Deoxypodophyllotoxin	190	Anticancer, Lymphoma	Inactive	-0.657±0.144	Inactive	-0.81±0.415	Inactive	Inactive	**OK**
345501	Deoxypodophyllotoxin	192	Anticancer, Melanoma	Inactive	-0.289±0.113	Inactive	-0.53±0.538	Inactive	Inactive	**OK**
345501	Deoxypodophyllotoxin	127	Anticancer, Renal tumor	Active	0.315±0.123	Active	-0.073±0.715	Inactive	Active	**OK**
72435	Picropodophyllin	328	Anticancer, Leukemia	Active	0.685±0.045	Active	-0.221±0.506	Inactive	Active	**OK**
72435	Picropodophyllin	190	Anticancer, Lymphoma	Inactive	0.232±0.128	Active	-0.566±0.427	Inactive	Active	
72435	Picropodophyllin	192	Anticancer, Melanoma	Inactive	0.094±0.165	Inactive	-0.014±0.592	Inactive	Inactive	**OK**
72435	Picropodophyllin	608	peptidyl-prolyl cis-trans isomerase FKBP1A	Inactive	-0.293±0.075	Inactive	-0.977±0.472	Inactive	Inactive	**OK**
72435	Picropodophyllin	1159580	Synergism from Chemical-Genetic Interactions	Inactive	-0.518±0.142	Inactive	-0.655±0.411	Inactive	Inactive	**OK**
119458	cleistanthin (B)	449703	Plasmodium falciparum	Active	-0.32±0.23	Inactive	-0.22±0.511	Inactive	Inactive	
119458	cleistanthin (B)	449704	Plasmodium falciparum	Active	-0.352±0.215	Inactive	0.097±0.414	Inactive	Inactive	
119458	cleistanthin (B)	602156	Plasmodium falciparum	Inactive	-0.645±0.023	Inactive	0.678±0.204	Active	Active	
160705	4'-Demethyl-deoxypodophyllotoxin	365	ribonuclease H1, E. coli	Active	-0.496±0.204	Inactive	-0.332±0.13	Inactive	Inactive	
160705	4'-Demethyl-deoxypodophyllotoxin	366	ribonuclease H1, Human	Active	-0.686±0.191	Inactive	0.474±-0.055	Active	Active	**OK**
160705	4'-Demethyl-deoxypodophyllotoxin	367	Virus, HIV-2	Inactive	0.075±0.354	Inactive	0.652±0.033	Active	Active	
164791	Podophyllotoxin acetate	608	peptidyl-prolyl cis-trans isomerase FKBP1A	Inactive	0.177±0.146	Inactive	-0.745±0.779	Inactive	Inactive	**OK**
164791	Podophyllotoxin acetate	1159580	Synergism from Chemical-Genetic Interactions	Inactive	-0.064±0.313	Inactive	-0.177±0.485	Inactive	Inactive	**OK**
159962	beta-Peltatin A methyl ether	328	Anticancer, Leukemia	Active	0.376±0.112	Active	-0.602±0.44	Inactive	Active	**OK**
